# Identifying knowledge important to teach about the nervous system in the context of secondary biology and science education–A Delphi study

**DOI:** 10.1371/journal.pone.0260752

**Published:** 2021-12-21

**Authors:** Pål Kvello, Niklas Gericke

**Affiliations:** 1 Department of Teacher Education, Norwegian University of Science and Technology, Trondheim, Norway; 2 Department of Environmental and Life Sciences, Karlstad University, Karlstad, Sweden; University of Macau, MACAO

## Abstract

Teaching about the nervous system has become a challenging task in secondary biology and science education because of the fast development in the field of neuroscience. A major challenge is to determine what content to teach. Curricula goals are often too general to guide instruction, and information about the nervous system has become overwhelming and diverse with ubiquitous relevance in society. In addition, several misconceptions and myths are circulating in educational communities causing world-wide confusion as to what content is correct. To help teachers, textbook authors, and curricula developers in this challenging landscape of knowledge, the aim of the present study is to identify the expert view on what knowledge is important for understanding the nervous system in the context of secondary biology and science education. To accomplish this, we have conducted a thematic content analysis of textbooks followed by a Delphi study of 15 experts in diverse but relevant fields. The results demonstrate six curriculum themes including *gross anatomy and function*, *cell types and functional units*, *the nerve signal*, *connections between neurons*, *when nerve signals travel through networks of neurons*, and *plasticity in the nervous system*, *as well as* 26 content principles organized in a coherent curriculum progression from general content to more specific content. Whereas some of the principles clarify and elaborate on traditional school biology knowledge, others add new knowledge to the curriculum. Importantly, the new framework for teaching about the nervous system presented here, meets the needs of society, as expressed by recent international policy frameworks of OECD and WHO, and it addresses common misconceptions about the brain. The study suggests an update of the biology and science curriculum.

## Introduction

Teaching about the nervous system has become a challenging task, particularly in compulsory science education, where the common goal is to prepare students for societal discourse as responsible citizens, rather than for a specific career within the field of neuroscience. One major challenge is to determine what content to teach. Curricula goals are often too general to guide instruction, and information about the nervous system has become overwhelming and diverse with ubiquitous relevance in society. This is documented by a 40% increase (27157 to 37319) in the annual number of neuroscience articles published from 2006 to 2015 [1] and a recent growth of several “neuro”- inspired academic disciplines including neuro-philosophy, neuro-ethics, neuro-history, neuro-education, neuro-economics [2] and neurorobotics [3], to name a few. Computer scientists apply computer-relevant knowledge of the nervous system to push the development of computing and robotics technology [4, 5] economists apply economy-relevant knowledge to understand and predict human decision-making [6], and educators are eagerly trying to apply education-relevant knowledge to increase their students’ learning outcomes [7, 8]. Selecting content to teach from such a diverse and ubiquitously relevant topic is further complicated by the numerous misconceptions which have been implemented into educational material [7, 9, 10] causing a world-wide confusion among teachers as to what content is correct and incorrect [11–19]. It is, for example, commonly held by teachers around the world that we only use 10% of our brain [8]. In addition, many teachers believe that brain activity depends entirely on the external environment, and that our feelings of happiness, anger and fear is experienced with our heart and not our brain [12, 19]. Consequently, there is currently a high level of confusion concerning what content to teach on this topic in biology and science education at all levels of the school system including teacher education.

To help teachers determine what content to teach, textbook authors what to write and curricula developers to define goals of major importance in this overwhelming, diverse and confusing landscape of knowledge, the aim of the present study is to identify the expert view on what knowledge is important for understanding the nervous system in the context of secondary biology and science education. To accomplish this, we have conducted a thematic content analysis of textbooks followed by a Delphi-study including experts in a variety of relevant fields, including neurobiology, medicine, psychology, and different subfields of neuroscience like systems neuroscience, cognitive neuroscience and computational neuroscience to find out what knowledge they think is important for understanding the nervous system in the context of biology and science education in secondary school.

### Background

Knowledge about the nervous system is part of the broader concept termed neuroscience literacy which has received much attention during the last two decades because of the major advances in the field of neuroscience and because of the relevance of this knowledge in society [7, 8, 15, 16, 20–23]. Several projects have been initiated to increase this type of literacy among the public [24–26], indicating that current compulsory education is insufficient. So, what do students learn about the nervous system in school? A review of the curricula of several countries shows that English students, for example, should learn about the relationship between the structure and function of the human nervous system [27]. Swedish students should know how the nervous system regulates the organism [28], and Norwegian students should be able to compare the nervous system and the hormone system, and describe how drugs, toxins and doping affect the signaling systems [29]. Similar goals can be found in biology and science curricula all over the world, making this core curriculum in compulsory biology and science education. An apparent feature of these goals is that they are quite general and therefore not very suitable to guide instruction. Almost anything in the field can fit within the frame of these goals. Therefore, the goals need to be specified into core ideas and concepts before being realized in the classroom. This responsibility is given to teachers who commonly relies on general biology and science textbooks often written by people without a strong background in neuroscience. Some books are sparse on information, whereas others are rich. The challenge faced by the teacher, however, is to select the essentials without getting lost in the details.

#### Traditional content

**Fig 1:**
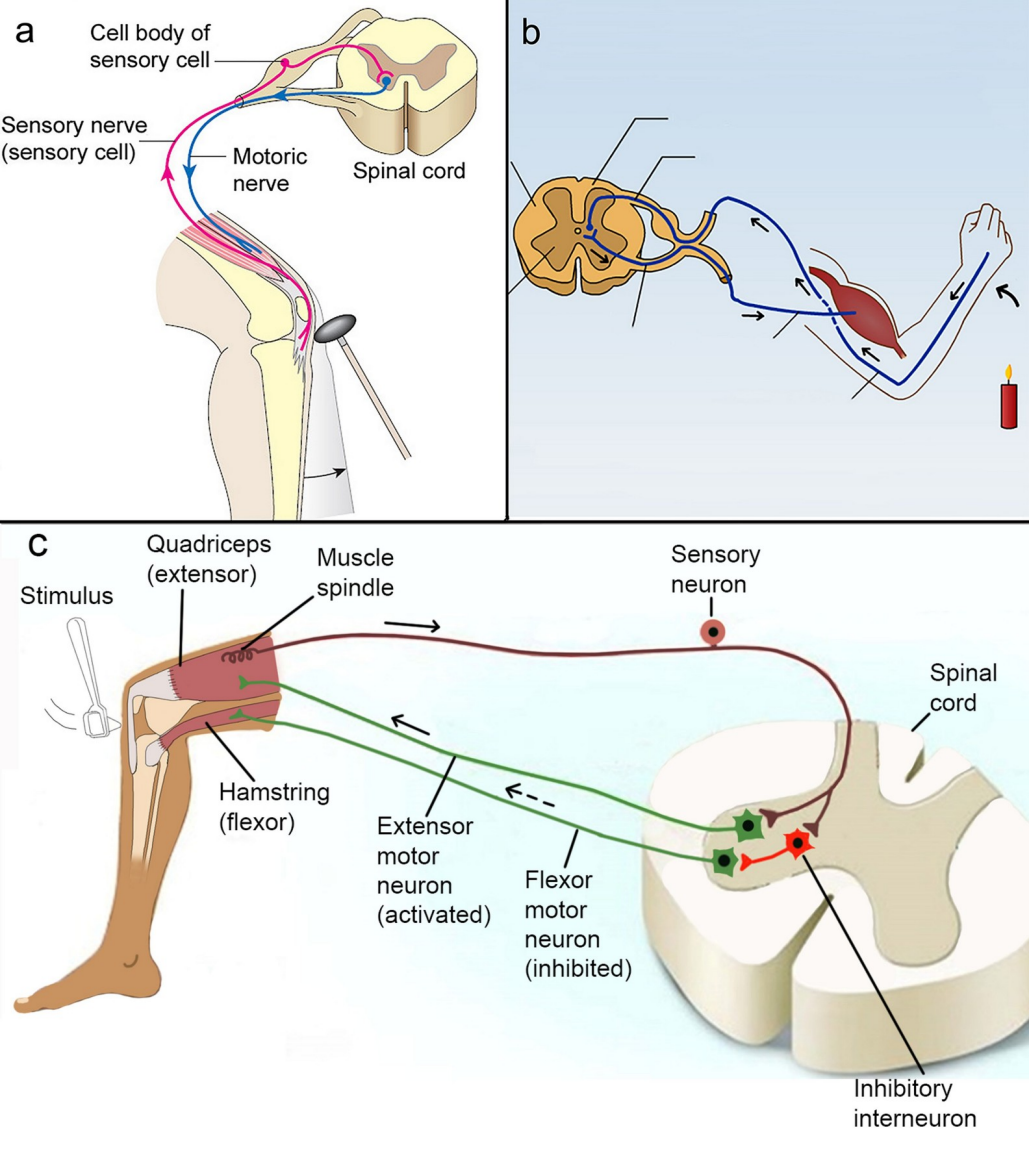
The knee-jerk- and withdrawal reflexes. (a) The knee-jerk reflex illustrated in a secondary school textbook (reproduced and translated with permission from [35]). (b) The withdrawal reflex as illustrated in a national digital resource for teaching material (reproduced under the Creative Common license). (c) The knee-jerk reflex with a neural network similar to figure 10–1 A in [37]. The reflex is elicited by tapping the tendon of the Quadriceps (extensor) muscle with a reflex hammer just below the knee cap. This pulls on the tendon and stretches the extensor muscle as well as the muscle spindle which is a sensory organ within the quadriceps muscle. This activates the sensory neuron which sends nerve signals into spinal cord. In the spinal cord the sensory neuron splits into two terminal branches. One branch conveys the nerve signal through an excitatory synapse to the extensor motor neuron which passes the signal on to the extensor muscle making it contract. The other branch conveys the nerve signal to the inhibitory interneuron which passes the signal on through an inhibitory synapse to the flexor motor neuron which becomes inhibited. This makes the flexor muscle relax while the extensor muscle contracts, and the result is an extension of the lower leg. In the illustration, each extensor and flexor motor neuron represent a population of many cells.

Traditionally, the curricula goals on the nervous system have been taught as any other organ system, like the respiratory- or the circulatory systems. Its anatomy and location within the body are commonly described, followed by relating its structure to function at the level of single cells, organs and organ system. However, progress in neuroscience during the last decades [26, 30–32] has reminded us that the nervous system is more than its physiological counterparts. The nervous system has several additional levels of operation, including neurons connected in networks. Neural networks neither belong to the cellular level, nor the organ- or organ system level. Understanding the nervous system through the operations of neural networks is described as a systems level understanding [33, 34], and it is at this level the logic of nervous system function is most apparent. Consequently, it is the focal level in the field of neuroscience. Although secondary school textbooks usually contain neural networks like the knee-jerk or withdrawal reflexes [35, 36], these elementary networks, as well as their rudimentary and often erroneous apparency in teaching material (Fig 1A and 1B), is no longer the optimal representation of how the nervous system works (Fig 1C demonstrates a more realistic version of the knee-jerk reflex as illustrated in [37]). As the prime investigator of these reflexes, the Noble laureate Charles Sherrington, himself pointed out when he saw his monograph on the topic 40 years after its first appearance, today more than seventy years ago:

“The volume here reprinted concerns itself predominantly with the type of motor behavior which is called “reflex”; it might give the impression that in reflex behavior it saw the most important and far-reaching of all types of “nerve” behavior. That is in fact not so [38].”

#### New content

Since reflexes were the first neural networks to be thoroughly studied at the anatomical, physiological and behavioral level, they represented our best understanding of nervous system function at the time. Today, however, there are several alternatives with more relevant applications in society. More than fifty regions of the nervous system of different animal species have been described in the format of neural networks down to the level of synapses, by numerous research groups around the world [39, 40]. Many of the regions are still fragmentary described, but they still provide important knowledge of how nerve signals traveling through networks of neurons may accomplish simple tasks such as sound location [41] and determining the direction of moving objects [42], but also more complex tasks like spatial navigation [43], learning and memory formation [44], as well as the process most closely associated with consciousness, namely working memory [45]. These abilities appear when nerve signals travel through networks of neurons [33], which apparently perform computations comparable to the logic of Bolean functions: AND, OR, NOT [46]. Consequently, knowledge about the nervous system at the network level may ultimately allow us to describe the most complex of human abilities at a synaptic resolution by the logic of mathematics. Therefore, in view of the recent progress in linking neural networks to complex human behavior which is progressively bridging the gaps between natural sciences, social sciences and humanities, it is surprising to see that the hundred-year-old knee-jerk reflex still is the systems understanding mediated by compulsory education.

#### Society’s current need for knowledge about the nervous system

Recent advances in linking neural networks to mental functions and behavior is progressively opening terrain to meet the diverse needs for knowledge about the nervous system in society. One of these needs, as pointed out by the WHO, is knowledge of mental, neurological and substance use disorders, which is important to reduce stigma and discrimination, and to improve public health [47]. Along the same line is the need for knowledge about the emotional and the physical aspects of the nervous system which has been emphasized by the OECD [26]. The emotional and the physical aspects have received little attention compared to the cognitive aspect. It is, however, important to recognize that the emotional, the physical and the cognitive aspects, often referred to as the “body and mind”, depend heavily on each other. It is particularly needed to understand these aspects in adolescents since this phase of life is more challenging than other phases. Adolescents struggle to focus attention, solve problems, and support relationships, and knowledge about the underlying neural mechanism may be important to provide effective teaching and learning strategies at this vulnerable developmental stage [26]. According to Blakemore [48] and MacNabb [49], this knowledge should be included in secondary school because adolescents might be interested in, and could benefit from, learning about the changes that are going on in their own brains. Another important need is knowledge of learning and memory, and particularly that learning is a lifelong activity with health benefits. This knowledge is important in order to understand that learning is not only granted the young, and it does not end with basic schooling [26]. Learning can and should take place throughout life. Other needs of knowledge include topics like the conception of man, self-conception, consciousness and free will, which are considered important in compulsory education because of the recent development of brain imaging techniques and brain-computer interfaces [20]. The presentation of such man-machine related issues in mass media seems to foster everyday myths about mind-reading and human manipulation, causing students to associate neuroscience with something dangerous and negative, instead of the positive outcomes as in the treatment of neurogenerative diseases [20]. Since neuroscience has become more important in societal discourse, a better understanding of the topics would be necessary in order to deal with neuroscientific information, and to participate successfully in society as responsible, reflective citizens. Myths about the nervous system “neuromyths” are a widespread problem, and since many of them have been developed as ideas about, or approaches to, how we learn, they must be dispelled in order to prevent education running into a series of dead ends [26]. It is therefore important to bring them into the classroom [13] for critical reflection [20]. One common neuromyth is that we only use 10% of our brain. This is a gold mine for marketers who claim to offer educational products that facilitate something they call “whole-brain” learning [9, 13, 50]. Unfortunately, it has a substantial cost for students and parents who risk wasting money and time on inefficient products [51]. Another myth is that all important aspects of brain function are determined by the age of three, and a third myth is that there are critical periods in life when certain matters must be taught and learnt. Other myths include, but are not limited to, the incorrect belief that brain activity depends entirely on the external environment, and that our feelings of happiness, anger and fear is experienced with our heart and not our brain [12, 19]. Including all these topics within the traditional class-time of a couple of hours is an impossible task, therefore a selection must be made.

#### Selecting content to teach about the nervous system

In general, selecting curriculum content for any subject is a complex task that should ideally involve several stakeholders, including scientists, teachers, students, textbook authors, parents, business enterprises, politicians, etc. Each may have their specific view, and even if they are conflicting, they may still be relevant in their context, whether it is a mother from a small drug-infested community, a local politician from an industrial city fighting with pollution, or a large business enterprise competing on a global arena. However, if the goal is to facilitate student’s understanding of how a particular system works, one would obviously benefit from involving people who are experts on that system and experts on teaching. These experts would probably be important to stimulate deep learning and understanding as opposed to surface-learning of disconnected facts [52, 53]. One approach to obtain the expert view has been conducted by brainfacts.org [54]. Their result is a list of 39 principles organized under eight major themes. The principles have been correlated to the U.S. National Science Education Standards to indicate its relevance for the K-12 curricula. Unfortunately, the list seems to lack several pieces of basic information on the topic, like the major divisions of the nervous system, important for understanding the overall relationship between nervous system structure and function. Information about neuron anatomy and the concept of inhibition, both fundamental for understanding nerve signal processing, are also lacking. Furthermore, the direction-specific propagation of the nerve signals and the specificity of connections between neurons, commonly referred to as the concept of dynamic polarization and connectional specificity, respectively [55], are also missing. Both concepts were adduced by the founding father of cellular neuroscience and Nobel laurate Santiago Ramón y Cajal [56], and they are crucial for the ability to read and understand neural network diagrams (circuit diagrams) commonly used in textbooks to explain the knee-jerk- and withdrawal reflexes. Such diagrams have recently been embraced by the neuroscience community for their simple way of communicating structure–function relationships which in a written format would appear as a catalog of detailed information [39, 40]. Finally, the process leading up to the Brainfact-principles is marginally described, and information about the people involved is missing, thus making the validity of the principles questionable. Therefore, the present study is an approach to obtain an expert view which is more valid and relevant for secondary biology and science education.

## Methods

The research design of this study consists of a thematic content analysis of textbooks [57] followed by a four round Delphi-study [58–60] to identify the expert view on what knowledge is important for understanding the nervous system in the context of secondary biology and science education.

### The Delphi technique

The Delphi technique is a common research approach for gathering expert’s opinions on a matter. The goal of the method is to reach consensus on the matter which subsequently can be used as a framework for making decisions [58–60]. In the present study, the Delphi technique was used to reach consensus among experts on what content is important in order to understand the nervous system in the context of secondary biology and science education. This content can later be used by, for example, curriculum developers, textbook authors and ultimately teachers, to make decisions about what to include in the biology curriculum and what to teach in the classroom. The advantage of the Delphi technique as compared to traditional discussion groups, is that it relies on anonymity among the expert participants, which reduces conformity pressures. In addition, the anonymity requirement eliminates the need to meet at a particular time or place. Therefore, building consensus through this method is not limited by time or space, or the volume of the participants’ voice. There are however, two main challenges: First, there is a risk of having unqualified personnel in the group of experts. Second, the method is time-consuming, and since experts usually are fully occupied with their own projects, the number of participants may be low, and they commonly drop out during the process before consensus is reached [61]. In the present study, several precautions were taken to minimize these challenges.

#### Initiatives to obtain qualified personnel in the group of experts

To minimize the risk of having unqualified personnel in the group of experts, we made several selection criteria [60, 61]. To make sure the participants had the right type of qualifications, our first criterion was to invite people with expertise on the nervous system. This was accomplished by only inviting people with both teaching and research experience on the topic as well as being currently working on the topic. Furthermore, to ensure a broad representation of the topic, our second criterion was to invite people from a variety of relevant disciplines including neurobiology, medicine, psychology, and different subfields of neuroscience like systems neuroscience, cognitive neuroscience and computational neuroscience. This criterion was further strengthened by inviting people from different universities, and from different nationalities, including both males and females. To make sure that the level of expertise was high, our third criterion was to only invite people with a PhD-degree in the field. This criterion was further strengthened by including prominent scientists with long and advanced experience in both research and teaching (more than 20 years) on the nervous system at different university levels including bachelor, master and PhD levels. In addition, one of the experts had five years of experience from science teaching at the teacher education program. To increase the likelihood of attendance and completion of the study, many of the experts were contacted physically and invited in person. In addition, we followed the advice of Hsu & Sandford [62], by asking some of our initial contacts to introduce us and our project to other potential participants. In total, eighteen experts were invited to participate of which fifteen responded in one or more rounds according to our request (Table 1). This number is in line with Okoli and Pawlowski [63], who recommend 10–18 experts, although the method has been successfully completed with as few as four [64]. The experts had between 5 to 30 years of both teaching and research experience on the nervous system. The study took place in Norway, which has an international high level of expertise in the field as indicated by the Nobel prize in physiology or medicine in 2014.

**Table 1:** Demographic information about the experts attending the study.

Demographic item		N	%
Gender	Male	9	60
	Female	6	40
Position	Professor	7	46
	Associate professor	6	40
	Researcher	1	7
	Postdoctor	1	7
Area of expertise	Systems Neuroscience	7	46
	Cognitive neuroscience	2	13
	Computational neuroscience	1	7
	Neurobiology	2	13
	Psychology	2	13
	Medicine	1	7
University (nr)	1	2	13
	2	11	73
	3	1	7
	4	1	7
Nationality	Norway	8	53
	Netherlands	2	13
	Germany	1	7
	England	1	7
	Portugal	1	7
	USA	1	7
	Turkey	1	7

#### Initiatives to increase the likelihood of expert attendance and completion of the study

After an informal feedback from the panel of experts, and due to the large size and diversity of the neuroscience field, we decided to use a research design where we provided the experts with an initial list of premade statements consisting of important information about the nervous system. This list was generated from a textbook analysis, and the experts were asked to comment, remove or add statements. For the experts, this design made the task more manageable, and similar to using a literature review as a starting point for a Delphi study, as argued by [60], a textbook analysis reduces the chance of excluding items that the experts may otherwise have omitted.

#### Textbook analysis: Categorization of textbook content

To develop a representative list of premade statements as a somewhat valid and reliable basis for expert revision, several initiatives were taken. First, the diversity of the neuroscience field was accounted for by including statements from several major neuroscience topics. These topics were identified by a thematic content analysis [57] were we categorized the content in four internationally recognized textbooks used in neuroscience classes at the university level, including Principles of neural science [37], Fundamental neuroscience [65], Neuroscience [66], and Neuroscience: exploring the brain [67] (S1 and S2 Tables). The categories were essentially made from the table of content in each book separately and subsequently compared to find commonalities. This process resulted in six major categories of neuroscience topics which were common to all four books including 1) Structure/organization of the nervous system, 2) Cell structure and function, 3) Sensory systems, 4) Motor systems, 5) Plasticity, 6) Cognition and other complex brain functions. These categories are in line with the basic features of the nervous system which, according to [55], should be in focus when learning about the nervous system.

#### Identification of important neuroscientific information in the textbook

To make sure the premade statements included important information, we scrutinized the most extensive book [37] for specific knowledge considered important for understanding the function of the nervous system. This was accomplished by searching for information where terms like *principle*, *concepts*, *chief idea*, *tenet*, *central*, *fundamental*, *essential*, *basic*, *elementary* and *crucial* were used to emphasize its importance. Through this process, we identified and collected all citations where the terms *principle* and *concept* were used in a relevant context (an irrelevant context for the term *principle* was for example: “…, in principle, such a study may not be insurmountable using current methods”, p. 386). We also collected one citation for each of the terms: *chief idea*, *tenet*, *central*, *fundamental*, *crucial*, and *basic*. This resulted in 59 citations from 30 chapters (S3 Table), and these citations formed our initial database of important information about the nervous system. The citations were subsequently grouped according to the six major neuroscience topic categories in order to verify that they represented the diversity of the neuroscience field (S3 Table).

#### Selection of relevant information and the formulation of premade statements

By reading through the citations, we quickly realized that much of the information seemed too advanced for secondary school level. Therefore, we conducted an initial filtering process by discarding citations we considered too advanced. This procedure left us with 30 citations which were lacking the most advanced information but still covering all six major neuroscience topic categories (S3 Table). Finally, to formulate coherent and precise statements with the important information, we combined the 30 citations in ways we found suitable, and we added relevant details from the literature. Through this procedure, we formulated 22 statements that we considered central to understanding the nervous system (S4 Table). In addition, we found it necessary to include two more statements. The first (statement nr 4), derived from [68], was included because of its’ fundamental link to the driving forces of the nerve signal, and the second (statement nr 22), derived from Brainfacts.org [54], was included because of its major implications in health-related issues and because of its’ rather recent discovery. Thus, our final list consisted of in total 24 premade statements (Table 2).

**Table 2:** Initial list of statements considered important knowledge about the nervous system (premade statements).

Nr	Statements
1	Neurons are the elementary building blocks and signaling elements of the nervous system
2	Neurons consist of three main structures: a cell body which is the metabolic center of the cell, dendrites which receive signals from other cells, and an axon with branches that sends signals to other cells
3	The nervous system can be organized into three types of neurons: a) Sensory neurons which bring information to the nervous system, b) Motor neurons which send information to muscles and glands, and c) Interneurons which send information between neurons
4	The nerve signal is an electrochemical signal that carries information
5	The nerve signal consists of three main types of signals: 1) graded electrochemical signals which mainly occur in the dendrites, 2) all-or-none electrochemical signals (action potentials) which mainly occur in the axon, and 3) graded chemical signals which occurs between neurons
6	Action potentials are the signals by which the brain receives, analyzes, interprets, and conveys information
7	Inside neurons, the nerve signal flows in one direction only: from the dendrites to the axon terminals
8	A neuron sends a nerve signal to other neurons only if the sum of its received signals is above a certain threshold
9	Nerve signals pass from one neuron to the next at specialized zones called synapses
10	At synapses, the nerve signal is transmitted from one cell to the next either by diffusion of molecules over a tiny gap (chemical synapses) or by electrical current (electrical synapses)
11	At chemical synapses, the nerve signal is transmitted in one direction only: from the axon terminals of the presynaptic cell to the dendrites of the postsynaptic cell
12	There are two main functional types of chemical synapses: Excitatory synapses which increase the probability of the postsynaptic neuron to send an action potential, and 2) Inhibitory synapses which decrease the probability of the postsynaptic neuron to send an action potential
13	A neuron receives both excitatory and inhibitory nerve signals from other cells, but can only send one type of signal, excitatory or inhibitory, to all its postsynaptic cells, not both
14	Synapses are formed by genetic programs during embryonic development but are modified through interactions with the internal and external environment
15	The effectiveness of synapses changes as we learn new things, and these changes are necessary to form memories
16	Each neuron in the central nervous system receives information from many other neurons and sends information to many other neurons to form networks and share information
17	Each neuron makes specific connections with certain postsynaptic target cells but not with others
18	Our perceptions, thoughts, feelings and behavior are mainly determined by which pathways the nerve signal takes through the network of neurons
19	The intensity of our perceptions and actions are mainly determined by the frequency of action potentials elicited by the sensory and motor neurons, respectively. High frequency gives rise to high intensity and low frequency to low intensity
20	The brain has a continuous self-sustaining activity. Sensory input cannot stop or start this activity, only modify it
21	The brain has distinct regions that are specialized for different functions, like perception, movement, language, thoughts, emotions, etc. However, different brain regions are interlinked, and proper brain function requires coordinated action of neurons in many brain regions
22	Brain structure and function is maintained by regularly challenging the brain with physical and mental activity–“use it or lose it”
23	The nervous system influences and is influenced by all other body systems (e.g., cardiovascular, endocrine, gastrointestinal and immune systems)
24	A properly functioning nervous system requires support from other types of cells, particularly glial cells

The statements were made from a textbook analysis.

### Data collection and analysis using the Delphi technique

When the group of experts had been chosen and the list of premade statements had been made, we sent emails to each of the experts separately. The email contained the list of premade statements and a short text describing the project and why the respective expert had been selected. They were informed that the project was conducted in the context of students in biology and science education at the level of secondary school. The experts were asked to review the statements in relation to the following question “What can be considered basic principles or key information about the nervous system?”. In addition, we particularly asked them to focus on the following questions:

1. Are any of the statements wrong or unclear / imprecise?
2. Are there any statements that should be omitted (which are not so central)?
3. Are there any other statements that should be added?
4. Are the statements simplified too much?
5. Other things?

These questions would give some guidance to their feedback but still allow the experts to provide open responses which is common in the first round of the Delphi technique [60].

#### Round one

In the first round, all fifteen experts participated, and their open responses to the 24 premade statements were carefully read through and collected in a document organized according to the respective statements. This resulted in an overview of all responses from all experts to each of the statements (S5 Table). The responses were initially divided into two large groups: 1) information agreeing with the statements and 2) information calling for a change. The information agreeing with the statements was analyzed quantitatively by calculating the percentage of agreement. This is common in Delphi studies to document the progress towards consensus. It showed an average agreement of 82%. The information calling for a change was analyzed qualitatively, which is necessary for making the appropriate adjustments of the statements. It consisted of 66 comments, of which 65 called for a change in the existing statements and only one suggested removal of a statement (statement 19, Table 2). In addition, the experts suggested seven additional statements (S5 Table). The type of qualitative analysis used on this data was thematic coding [68]. This was conducted by searching each expert’s comments for concepts related to the nervous system (e.g. glial cells) as well as for action words (e.g. verbs like add, remove, replace, moderate, specify). From this analysis, the information calling for a change were grouped into 31 categories including for example “Add that glial cells are part of the nervous system”, “Remove or replace the term “maintain””, and “Moderate statement by adding the phrase “… commonly consist of …””. The number of experts providing comments within each category was also registered since this indicated how important the category was (its priority). Then, with all suggested changes at hand, represented by categories which were ranked by priority, we revised the relevant statements (see Tables 3 and 4 for an example). In general, we carried out all categories of changes suggested by two or more experts, but we also carried out changes suggested by single experts when this included correction of slightly erroneous information in the statements, or reformulations to specify, moderate or make the statements easier to understand. The validity of all changes was verified by a literature search. In some cases, the revision process required splitting a statement in two. This was usually done when the information in the statement appeared too much or too complex. In other cases, we found it suitable to merge two or more statements into one. This was usually done when the information in each of the statements was low and closely related or partly overlapping. In yet other cases, it was necessary to add statements. Based on this revision, the 24 premade statements were reduced to 22 statements.

**Table 3:** Transformation of a statement through the first round with the Delphi-technique.

Premade statement 22 (round one)	Comments from experts to this particular premade statement	Revision categories	Revised statement
Brain structure and function is maintained by regularly challenging the brain with physical and mental activity–“use it or lose it”	That is maybe a bit unspecific and may lead to wrong conclusions. It is certainly not true for everything. For example, we don’t usually forget how to ride a bicycle (or speak our mother tongue) even though we don’t use them. So either that point must be more specific or reformulated to make it true	Delete the phrase “use it or lose it” and be more specific.	5. Regularly challenging the brain with physical and cognitive activity helps sustain brain structure and function
	True, the "use it or lose it" principle is also more specific than just "mental activity" in many cases (and mistakes in others—such as strong emotional memories?	Delete the phrase “use it or lose it” and be more specific.	
	“use it or lose it”?	Delete the phrase “use it or lose it” and be more specific.	
	Nice point, but perhaps «maintain” is a bit strong–may sound like if you sit in the sofa your brain will actually degenerate. Would “sustain” be a better shade?	Remove or replace the term “maintain”	
	“Maintain” might not be the right word here?	Remove or replace the term “maintain”	
	I would like to see a statement about the plasticity of the nervous system, possibly in relation to “use it or lose it”	Add more statements on plasticity and memory	

The table shows an example of the transformation of one premade statement from round one of the Delphi process into the revised statement used in round two.

**Table 4:** Ranking of revision categories by priority.

Revision categories	Number of experts within each category	Priority
Delete the phrase “use it or lose it” and be more specific.	3	1
Remove or replace the term “maintain”	2	2
Add more statements on plasticity and memory, particularly emphasizing synaptic specificity	1	3

The table shows an example of how the revision categories for premade statement 22 in round one was ranked by priority. It is the same statement as used in Table 3. “Brain structure and function is maintained by regularly challenging the brain with physical and mental activity–“use it or lose it”.

After revising the statements, we also wanted to categorize the statements into overriding themes. To accomplish this, we analyzed the revised statements using thematic coding [68]. This was performed by searching each statement for concepts related to the nervous system, and subsequently assigning a common term or a short phrase that we found to be an appropriate representation of the majority of the concepts in the statement (see Table 5 for examples). The assigned terms or phrases them became our themes. We subsequently compared the themes of each statement, and the statements which were classified into the same theme, were grouped together. This resulted in six overriding themes of content: 1) Gross anatomy and function, 2) Cell type, structure and function, 3) The nerve signal, 4) Synapses, 5) Neural plasticity, and 6) Neural coding.

**Table 5:** The categorization of statements into overriding themes.

Statements revised after round one (concepts related to the nervous system are highlighted)	Concepts related to the nervous system	Themes
A neuron commonly consists of three main structures: dendrites which receive nervesignals from several other cells, a single branching axon which sends signals to several other cells, and a cellbody which maintains the cellmachinery	Neuron, dendrites, nerve signals, cells, axon, cells, cell body, cell machinery	Cell type, structure and function
The gradedpulses of chemicals make the receiving neurons generate gradedelectricalimpulses. Gradedelectricalimpulses larger than a certain voltagevalue (threshold) make neurons send actionpotentials. Actionpotentials entering the axonterminal make neurons send gradedpulses of chemicals	Graded pulses, neurons, graded electrical impulses, graded electrical impulses, voltage value, threshold, neurons, action potentials, action potentials, axon terminal, neurons, graded pulses	The nerve signal
The nervoussystem is commonly divided into the centralnervoussystem (CNS) which consists of the brain and the spinalcord, and the peripheralnervoussystem (PNS) which connects the CNS with the rest of the body	Nervous system, central nervous system, CNS, brain, spinal cord, peripheral nervous system, PNS, CNS, body	Gross anatomy and function
The nervesignal is dynamic and consists of three main types of impulses: a) Gradedelectricalimpulses which flow a short distance primarily from the dendrites to the beginning of the axon. b) All‐or‐noneelectricalimpulses (actionpotentials) which flow a long distance primarily from the beginning of the axon to the axonterminals. c) Gradedpulses of chemicals which primarily flow from the axonterminals, over a tiny extracellular gap, to the dendrites of the receiving neuron.	Nerve signal, impulses, Graded electrical impulses, dendrites, axon, All-or-none electrical impulses, action potentials, axon, axon terminals, Graded pulses, dendrites, neuron	The nerve signal

The table shows four statements revised after round one with relevant concepts highlighted (column 1), the highlighted concepts (column 2), and the themes assigned to represent the concepts and hence the statements (column 3).

#### Round two

In the second round, each statement was linked to a three-point Likert scale with the following alternatives: agree, disagree and neutral, as well as a free-text comment-field. The Likert-scale alternatives were included to allow for a more elaborate quantitative evaluation of the feedback than in the first round, and the free comment field was included to allow further adjustments of the statements. The statements were then sent back to the experts which were asked to choose one of the alternatives and comment on their choice. To the email, we also attached the document with all the experts’ comments from the first round (without names to preserve anonymity). According to [59], this information encourages experts without strong conviction to adjust their opinions closer to the majority, while those with strong convictions tend to retain their original opinion and argue for it. This part of the Delphi technique contributes to strengthen the validity of the data. The feedback in this second round, originating from eight experts, was again carefully read through and collected in a document organized according to the respective statements. The quantitative information from the three Likert-scale responses was calculated in percentage, showing an average agreement of 82%, an average neutral of 13% and an average disagreement of 5%. The qualitative information from the free comment field consisted of 49 comments from seven of the experts. By following the same revision procedure as in round one, the 22 statements resulted in 25 new statements. The six overriding themes mostly remained the same except for a minor reformulation and a change in sequence. The statements were then sent back to the experts for a third review.

#### Round three and four

**Fig 2:**
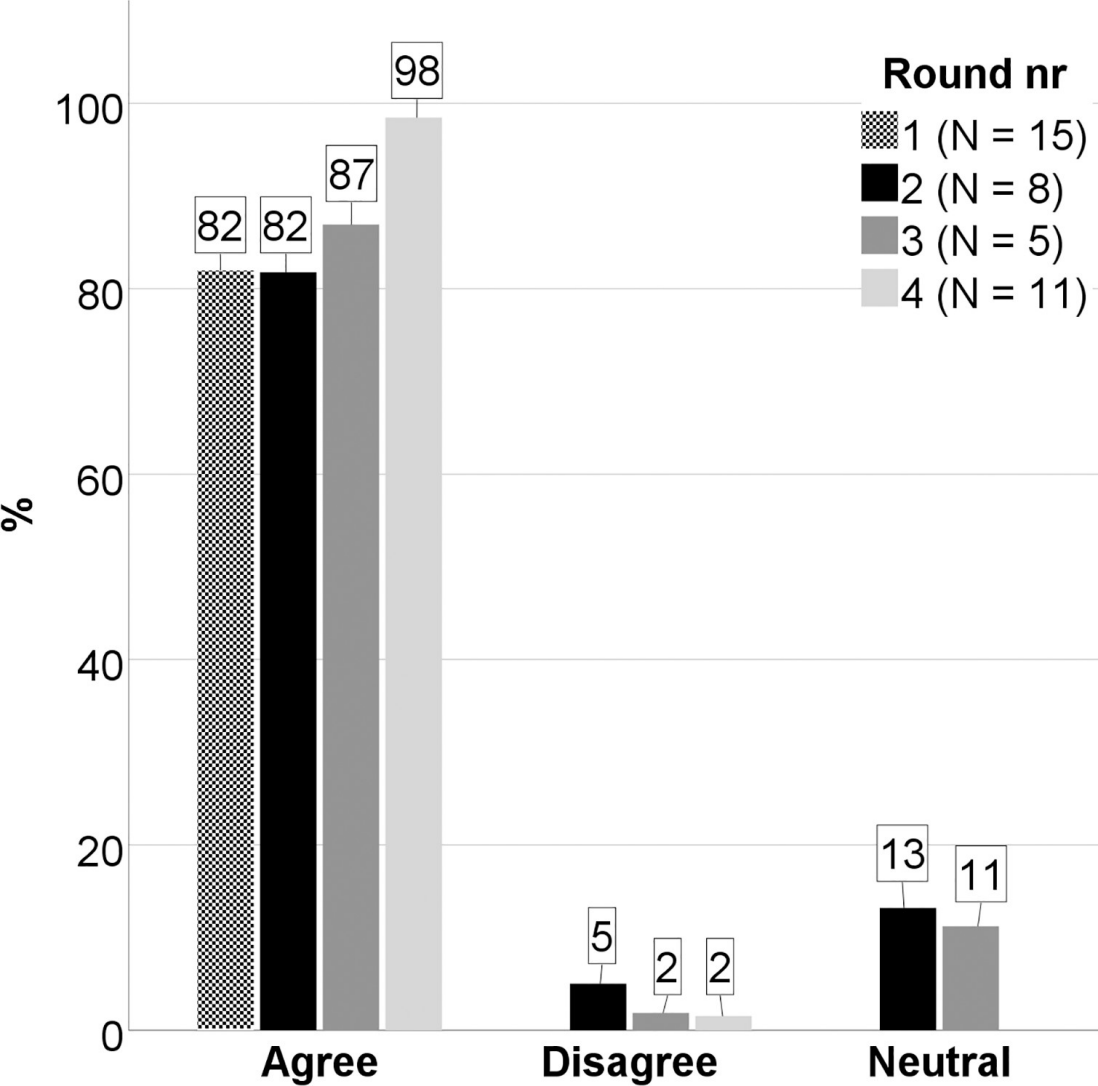
The quantitative progression towards consensus during the Delphi-process. Average percentage of experts agreeing, disagreeing and being neutral to the statements during four rounds with the Delphi-method. In the first round, the experts responded freely, and the agreements were interpreted from their answers. In the second, third and fourth round, the experts responded to a three-point Likert-scale with the alternatives agree, disagree and neutral.

Round three and four followed the same procedure as round two. The feedback in these rounds originating from five and eleven experts, respectively, was carefully read through and collected in a document organized according to the respective statements. Average percentages of agreements, disagreements and neutral was calculated and compared for each round (Fig 2). The level of agreement on each principle was classified according to [69], which means that consensus is achieved when the level of agreement is 100% (all experts agreed) and majority is achieved when the level of agreement is less than 100% but more than 50%. Based on the high percentage of agreements in the fourth round (>95%), and a subjective assessment of the stability of the disagreements as well as the low number of associated comments in the two last rounds, we concluded that four rounds were enough. The quantitative progression towards consensus is demonstrated in Fig 2 as an increase in agreements and a decrease in disagreements and neutral responses. The qualitative progression towards consensus is illustrated by an example of the transformation of one premade statement into a final principle through all four rounds of the Delphi process (Tables 6 and 7). All feedback from which the results are based on is presented in S5 Table.

**Table 6:** Transformation of a statement throughout the Delphi process.

Round one (premade statement 22)	Comments from experts	Revision category	Revised statement
Brain structure and function is maintained by regularly challenging the brain with physical and mental activity–“use it or lose it”	That is maybe a bit unspecific and may lead to wrong conclusions. It is certainly not true for everything. For example, we don’t usually forget how to ride a bicycle (or speak our mother tongue) even though we don’t use them. So either that point must be more specific or reformulated to make it true	Delete the phrase “use it or lose it” and be more specific.	5. Regularly challenging the brain with physical and cognitive activity helps sustain brain structure and function
	True, the "use it or lose it" principle is also more specific than just "mental activity" in many cases (and mistakes in others—such as strong emotional memories?	Delete the phrase “use it or lose it” and be more specific.	
	“use it or lose it”?	Delete the phrase “use it or lose it” and be more specific.	
	Nice point, but perhaps «maintain” is a bit strong–may sound like if you sit in the sofa your brain will actually degenerate. Would “sustain” be a better shade?	Replace or delete the term “maintain”	
	“Maintain” might not be the right word here?	Replace or delete the term “maintain”	
	I would like to see a statement about the plasticity of the nervous system, possibly in relation to “use it or lose it”	Add more statements on plasticity and memory	
**Round two (revised statement from round one)**	**Comments from experts**	**Revision category**	**Revised statement**
Regularly challenging the brain with physical and cognitive activity helps sustain brain structure and function	These can even enhance brain structure and function, not just sustain them	Specify cause and effect on brain health	24. Regularly engaging in learning activities enhances brain function and defers age-related decline in brain function
	I agree about the principle, but not the exact phrasing. Perhaps I would prefer something like “exercising” instead of “challenging”	Specify cause and effect on brain health	
**Round three (revised statement from round two)**	**Comments from experts**	**Revision category**	**Revised statement**
Regularly engaging in learning activities enhances brain function and defers age-related decline in brain function	Not wrong, and I like that the point is included. Just worried if it can be understood very literally or misunderstood. For example, you can’t "train yourself out of degeneration", even if it is one factor. And too much (stress) is not good either. I think it is good to convey that it is healthy to use your brain in healthy ways, but it’s not the case that it is your "fault" if you still have an agerelated decline in brain function. How to convey this correctly to school kids? But probably you have thought it well through! (And I like this one better than some previous version which I think I criticised!)	Reword to moderate the statement	25. Engaging in learning activities enhances brain function and defers age-related decline in brain function
**Round four (revised statement from round three)**	**Comments from experts**	**Revision category**	**Final principle**
Engaging in learning activities enhances brain function and defers age-related decline in brain function	None	None	Principle 25. Engaging in learning activities enhances brain function and defers age-related decline in brain function

The table shows an example of the transformation of one premade statement into the final principle through four rounds with the Delphi process.

**Table 7:** Ranking of revision categories by priority throughout the Delphi process.

Revision category round one	# 1	Pri	Revision category round two	# 2	Pri	Revision category round three	# 3	Pri	Revision category round four	# 4
Delete the phrase “use it or lose it” and be more specific.	3	1	Specify cause and effect on brain health	2	1	Reword to moderate the statement	1	1	None	None
Replace or delete the term “maintain”	2	2								
Add more statements on plasticity and memory	1	3								

The table shows an example of how the revision categories for premade statement 22 were ranked by priority through four rounds with the Delphi process. It is the same statement as used in Table 6. “Brain structure and function is maintained by regularly challenging the brain with physical and mental activity–“use it or lose it”. # 1 = Number of experts within each category in round one, # 2 = Number of experts within each category in round two, etc. Pri = Priority.

## Results

The results are based on an analysis of four textbooks as well as 929 responses from fifteen experts through four rounds of feedback using the Delphi technique. The results show a high level of consensus on six knowledge themes including *gross anatomy and function*, *cell types and functional units*, *the nerve signal*, *connections between neurons*, *when nerve signals travel through networks of neurons* and *plasticity in the nervous system*, and 26 content principles considered important knowledge for understanding the nervous system in the context of secondary biology and science education (Table 8). Consensus was achieved among all eleven experts attending the last round on all themes and on all except three principles. The exceptions included principle number 15, 16 and 19. They did, however, reach a majority of 91%, 82% and 91% agreement, respectively. Concerning principle 15, only one expert disagreed, arguing that it is beyond our knowledge: “There are elements we still do not know” (Expert F). Concerning principle 16, two experts disagreed. One of them argued that it was weakly formulated: “It is weakly formulated and probably doesn’t deserve a place on the list” (Expert E). The other expert did not add any comments (Expert F). Concerning principle 19, only one expert disagreed, arguing against the statement’s reference to feelings: “Principle 19 would work perfectly well without referring to feelings, which make it all sound a bit pseudoscientific” (Expert E).

**Table 8:** Knowledge themes and principles considered important for understanding the nervous system in the context of secondary biology and science education.

Knowledge Themes and Principles
**Gross anatomy and function**
1. The vertebrate nervous system comprises the brain, the spinal cord, ganglia and nerves. The brain and the spinal cord make up the central nervous system (CNS), whereas ganglia and nerves make up the peripheral nervous system (PNS). The PNS connects the CNS with the rest of the body.
2. The PNS consists of a sensory division which carries information about the external environment and the body into the CNS, and a motor division which carries information from the CNS to muscles and glands. Through this architecture, the nervous system influences and is influenced by all other organ systems (e.g., cardiovascular-, gastrointestinal-, muscular-, immune-, exocrine- and endocrine systems).
3. The motor division is commonly divided into a voluntary system (the somatic system) controlling our skeletal muscles, and an involuntary system (the autonomous system) controlling visceral organs. However, the two systems interact to support compatible physiological, emotional and behavioral responses.
4. The brain has a continuous self-sustaining generation of nerve signals. These nerve signals are not dependent on sensory input but can be modified by it.
**Cell types and functional units**
5. The nervous system consists of two major classes of cells:
a) Neurons, which receive, process and send nerve signals,
b) Glial cells, which protect and support the function of neurons
6. A neuron usually consists of three main structures:
a) a cell body
b) dendrites which receive nerve signals from several other cells.
c) a single branching axon with many terminals, which sends nerve signals to several other cells.
7. There are many types of neurons and they are commonly classified in two ways:
a) by where they receive and send signals:
i. Sensory neurons, which receive signals (light, sound, etc.) from outside the nervous system, transform them into nerve signals and send them to neurons in the central nervous system.
ii. Motor neurons, which receive nerve signals from neurons in the central nervous system and send them to muscles and glands.
iii. Local interneurons, which receive nerve signals from neurons in a region of the nervous system and send them to neurons located within the same region.
iv. Projection interneurons, which receive nerve signals from neurons in one region of the nervous system and send them to neurons in another region.
b) by what effect they have on their target cells:
i. excitatory neurons, which have a stimulating effect on nerve signal generation
ii. inhibitory neurons, which have an inhibitory effect on nerve signal generation
**The nerve signal**
8. The typical nerve signal is generated by neurons and consists of three types of pulses:
a) Graded electrical pulses (receptor potentials and synaptic potentials) which primarily flow from the dendrites to the beginning of the axon.
b) Ungraded electrical pulses (action potentials) which primarily flow from the beginning of the axon to the axon terminals.
c) Graded chemical pulses which primarily flow from the axon terminals, over an extracellular gap, to the dendrites of a receiving neuron. The chemical pulses propagate much slower than the electrical pulses.
9. When a neuron receives graded pulses of chemicals, it generates graded electrical pulses. If the graded electrical pulses are larger than a certain voltage value (threshold), the neuron generates action potentials successively until the voltage value goes below threshold. When the action potentials reach the axon terminals, the neuron releases graded pulses of chemicals.
**Connections between neurons**
10. Nerve signals pass from one neuron to the next at locations called synapses which are specialized zones for communication between neurons.
11. There are two main functional types of synapses:
a) Excitatory synapses which make the receiving neurons generate action potentials or increase their probability to do so.
b) Inhibitory synapses which prevent the receiving neurons from generating action potentials or decrease their probability to do so.
12. The chemicals used for communication between neurons at synapses are called neurotransmitters. There are many different types of neurotransmitters, but the most common effect on a receiving neuron is excitatory or inhibitory. The duration of the effect can vary from milliseconds to several minutes.
13. A neuron can receive both excitatory and inhibitory signals but can usually send only one of the types, excitatory or inhibitory, to all its target cells.
14. Neurons are connected in networks, but each neuron makes synapses with specific target cells, not with every cell around them. Thus, a nerve signal from a given neuron will only pass to a selected group of target cells rather than to all its neighboring cells.
**When nerve signals travel through networks of neurons**
15. The nerve signals carry information about the external environment and the body. This information can generate our sensations, perceptions, thoughts, feelings and behaviors.
16. What we sense, perceive, think, feel, and how we behave are mainly determined by two properties of the nervous system:
a) the specific network of neurons through which the nerve signals travel.
• e.g. in sensory parts of the nervous system, this carries information about the *identity* of the stimulus (a particular odor, sound frequency, color, etc.),
b) the time between successive action potentials travelling through the network.
• e.g. in sensory parts of the nervous system, this carries information about the *intensity* of the stimulus. Short time (high frequency) means high intensity and long time (low frequency) means low intensity.
17. Information from different sensory organs, like visual-, auditory-, olfactory-, taste- and tactile information, is carried by nerve signals travelling along neurons to distinct regions of the brain.
18. The brain has distinct regions for different functions including a variety of sensations, behaviors, language, etc. However, brain regions are interlinked by neurons, and each function depends on several regions.
19. The network of neurons responsible for our perceptions, emotions and behaviors interact all the time. This means that our *perceptions* influence how we feel and behave, but also that our *behavior* influences what we perceive and how we feel, and finally that our *feelings* influence what we perceive and how we behave.
20. To become *conscious of what* we are sensing, the nerve signals in the sensory neurons must be sufficiently strong (sufficient number and frequency of action potentials) to enter specific networks of neurons in the frontal part of the cerebral cortex. The required strength varies depending on how we feel, what we do and what the stimulus is.
21. Not all nerve signals from our senses are sufficiently strong to make us *conscious of what* we are sensing. However, the signals can still be strong enough to influence the networks of neurons responsible for our feelings, thoughts and behaviors. Consequently, we cannot always be aware of the reason for what we feel, think and do.
**Plasticity in the nervous system**
22. Synapses are formed and changed throughout life, and this process is influenced by individual experiences.
23. A synaptic change typically means that the influence a neuron has on its target neurons becomes stronger or weaker.
24. When you learn, each memory is stored as a change in specific synapses in the network of neurons involved in the learning activity. Some synapses may be strengthened, others weakened.
25. Engaging in learning activities enhances brain function and defers age-related decline in brain function.
26. The brain’s ability to change through experience varies over the lifetime and differs between brain regions. Sensory regions are particularly sensitive during early childhood, whereas frontal regions involved in cognitive functions are more sensitive later and the sensitivity lasts longer. Some changes are crucial for normal development, whereas others are detrimental, depending on the experience.

The principles are made from a textbook analysis and four consecutive rounds with revision based on feedback from a group of 15 experts using the Delphi technique.

## Discussion

The results showed that the expert’s view on what knowledge is important in order to understand the nervous system in the context of biology and science education at the secondary level can be expressed by 26 principles divided in six major themes including 1) gross anatomy and function, 2) cell types and functional units, 3) the nerve signal, 4) connections between neurons, 5) when nerve signals travel through networks of neurons, and 6) plasticity of the nervous system. We interpret this to mean that these principles are the expert’s view on what is important to teach at the secondary level.

Within each theme, consecutive principles are largely organized in a coherent curriculum progression from general content to specific content. This is demonstrated by the fact that most principles elaborate on a concept presented in the former principle. For example, principle 1 introduces the concept of the peripheral nervous system (PNS) which is further specified in principle 2 which introduces the concept of motor division which is further specified in principle 3. Thus, the principles logically build on each other step by step. This relationship between the principles is a prerequisite for systems knowledge [70] and deep learning [53], and it helps prevent surface-learning of disconnected facts as in a learning progression [71]. Consequently, the expert view as presented in this study, seems to facilitate systems thinking, deep learning, and a possible learning progression.

The themes are, to some extent, organized in a progression from simple to complex, or from elementary units to complex physical structures and mental phenomena. For example, themes one through four contain knowledge of basic anatomy and physiology which corresponds relatively well with traditional content, although there are some striking differences, as discussed below. Themes five and six, however, include knowledge about neural coding (how the world, inside and outside the body, is represented in the nervous system consciously or unconsciously) and neural plasticity (how the nervous system can change), which are rather new and involves the most complex functions of the nervous system. Hence, these two latter themes build upon the knowledge in the first four themes, and in contrast, mainly add new knowledge to the traditional content. In general, the principles are consistent with the curricula goals whose breadth allows for information about almost any part and any level of the nervous system, provided that structure is linked to function. Below, we discuss each principle separately.

### Gross anatomy and function

Principle 1 describes the gross anatomy and organization of the vertebrate nervous system. It is consistent with traditional textbook content [35, 36, 71], but missing from the brainfact-principles [54]. The principle makes up an essential first step to meet most of society’s need for knowledge about the nervous system by concretizing the neural substrate and spatially relating it to the rest of the body.

Principle 2 specifies principle 1 by describing two major divisions of the peripheral nervous system–the sensory and the motor divisions. It is consistent with traditional content as well as the brainfact-principles. However, the principle emphasizes the bidirectional communication between the CNS and other organs more than traditional textbooks. This knowledge is a necessary first step to meet society’s need for a better understanding of the interdependence between the body and the mind, as pointed out by the OECD [26].

Principle 3 specifies principle 2 by describing two major systems within the motor division of the peripheral nervous system. It is to some extent consistent with traditional textbook content but missing from the brainfact-principles. The principle is an important contribution to meet society’s need for understanding, not only the general interdependence between the body and the mind, but more specifically the interdependence between the emotional, physical and the cognitive. Through this principle, students learn that the voluntary and involuntary systems are coordinated to reach the common goal of the organism, and therefore highlights the unifying role of the nervous system.

Principle 4 describes the autonomous activity of nervous system. This knowledge is commonly lacking in traditional content e. g. [36, 72, 73], and it is also missing from the brainfact-principles. However, it provides an important contribution to meet society’s need for a better understanding of the conception of man, self-conception, consciousness and free will [20]. Through this principle, students may realize that our feelings, thoughts, and behaviors are not only generated as an immediate response to sensory stimulation. This is, in fact, a common misconception among teachers who believe that brain activity depends entirely on the external environment [12, 19]. However, stimulus-independent thoughts or “mind wandering” appears to be the default mode of the brain [74]. The principle could be exemplified by the noradrenergic- [75], serotonergic- [76], and/or dopaminergic neurons [77] in the brain stem, which contribute to the baseline activity in the nervous system and are crucial to obtain the level of wakefulness and attentiveness necessary for any complex behavior as well as any conscious mental state [78–81]. The principle could also be exemplified by our biological clock neurons in the hypothalamus which time the activity of brain stem neurons into the 24 hours cycles that we experience through our daily rhythm of sleep and wakefulness [82], feeding–fasting, blood pressure, renal function, and mental alertness [83].

### Cell types and functional units

Principle 5 names and describes the function of the two major cell types of the nervous system. This is consistent with traditional content, but not with the brainfact-principles which are lacking glial cells.

Principle 6 specifies principle 5 by naming the major anatomical compartments of the neuron and describing their main function. It is generally consistent with traditional content. However, traditional content often provides two statements which, although correct, may be confusing unless more specific information is added. This is the general statement that 1) a neuron is connected to many other neurons, and 2) that a neuron has many dendrites but only a single axon e. g. [35, 84]. This may lead to the misconception that neurons receive information from many other neurons but send information to only one or a few. Principle 6, however, may prevent this misconception by emphasizing that both the dendrites and the axon of a neuron connect to many other neurons. Thus, a single neuron receives information from- and sends information to several neurons. This may seem like an insignificant detail, but the principle of convergence and divergence is central to all efficient communication systems, and it is fundamental for understanding the logic of nervous system function at the network level. Thus, this principle provides important specifications in line with the focal level in the field of neuroscience. The brainfact-principles lack information about these major anatomical compartments.

Principle 7 specifies principle 5 by describing two common ways of classifying neurons of which only the first is consistent with traditional content. The second classification, based on what effect they have on their target cells, is usually omitted. Instead, all neurons are traditionally presented as excitatory e.g. [73, 84]. This is also the case for the brainfact-principles. This is unfortunate because of the crucial role of inhibitory neurons in most functions of the nervous system. For example, the central nervous system maintains a delicate balance between excitation and inhibition [85–88], allowing behavior to be rapidly controlled by an active upregulation and downregulation of neural activity. This property is analogous to the gas and brake pedals of a driving car [89], and it makes sense if one remembers the continuous self-sustaining generation of nerve signals in the brain, as stated in principle 4. Thus, the default mode of the nervous system is “driving” as opposed to standing still (inactive). Therefore, both inhibition and excitation can be used actively to change its activity. Furthermore, inhibition is necessary to understand functions as basic as the knee-jerk reflex and as complex as visual perception [90], selective attention [91] and social behavior [92], as well as neurological disorders like epilepsy [93] and autism [92]. For example, epileptic seizures would be a frequent experience without inhibitory neurons counteracting the excitation, and the knee-jerk reflex would not work without inhibition of the motor neurons which trigger contraction of the antagonistic hamstring muscles. This can be seen at the network level (see Fig 1C). Therefore, this principle adds important knowledge in line with the focal level in the field of neuroscience.

### The nerve signal

Principle 8 describes the three major components of the nerve signal and where each of them flow within and between neurons. This knowledge is quite different from traditional content which instead usually elaborate on the ionic mechanisms underlying the action potential. This is particularly emphasized in textbooks for upper secondary school e. g. [36, 73] as well as for teacher education [94], but some details on the topic are also included in textbooks for lower secondary school [84]. Principle 8, however, simplifies the topic by mainly emphasizing that the nerve signal is composed of pulses as opposed to a continuous signal, and that two of the pulses are graded in magnitude whereas one is ungraded. This knowledge is important in order to understand how neurons make the decision to send nerve signals. Presenting the nerve signal in a simplified way is consistent with the brainfact-principles which solely describes it as electrical and chemical signals. This raises the question whether the mechanisms described in detail by Hodgkin and Huxley in 1952 [95], earning them the Nobel prize in 1963, are still relevant for secondary biology and science education on the nervous system. The topic is complex and requires a considerable amount of classroom time. Is the gain worth the pain? Omitting it from the curriculum would certainly allocate much more time for newer and probably more relevant topics on the nervous system.

Principle 9 elaborates on the content of principle 8 by describing the temporal sequence of the three different pulses of the nerve signal as well as the casual relationship between them. It contributes information necessary for understanding the logic of nerve signaling without going into the details of ion transport which is common for traditional textbooks [36, 73].

### Connections between neurons

Principle 10 describes the typical zone of communication between neurons, the synapse. This knowledge is consistent with traditional content as well as the brainfact-principles.

Principle 11 specifies principle 10 by describing two major functional types of synapses, the excitatory and the inhibitory. This knowledge is generally lacking in textbooks for lower secondary school but present in upper secondary school textbooks [36, 73]. It provides important knowledge in line with the focal level in neuroscience. However, it may be wise to relate these excitatory and inhibitory synapses to excitatory and inhibitory neurons, respectively, otherwise they may promote the misconception that a single neuron, in general, can have both excitatory and inhibitory effects on its’ target neurons. Although existing, this is uncommon and makes no sense without additional information. Therefore, at this educational level it may be wise to stick to the generalization suggested by Eccles [96], that a neuron which has an inhibitory effect on one of its target neurons (through an inhibitory synapse), has an inhibitory effect on all its target neurons (through inhibitory synapses). This makes it an inhibitory neuron. The same generalization goes for the excitatory neuron. The brainfact-principles do not distinguish between synapses.

Principle 12 specifies principles 11 and 8c by adding knowledge about the chemicals used for communication between neurons at chemical synapses (neurotransmitters). Knowledge about neurotransmitters is usually lacking in textbooks for lower secondary school, but present in upper secondary school textbooks, and due to the large diversity of neurotransmitters, the information may become complex. The principle, however, simplifies the topic by reducing the number of outcomes of neurotransmitter action. This contributes to reduce the complexity and consequently make the topic of synaptic transmission more manageable for students. Information about neurotransmitters is missing from the brainfact-principles.

Principle 13 describes the functional difference between the input and the output of neurons. This knowledge is commonly lacking in traditional content. It is also missing from the brainfact-principles. However, it is important in order to prevent the misconception that a single neuron, in general, send both excitatory and inhibitory signals, also mentioned above, under principle 11.

Principle 14 points out the specificity of connections between neurons in a network, i. e. neurons are not randomly connected. This knowledge is commonly lacking or minimally present in traditional content e. g. [35, 84]. Current textbooks seem instead to focus on the large number of connections made by each neuron. This is unfortunate because it emphasizes complexity leading to confusion rather than specificity underlying the logic behind structure and function of neural networks. It is important to note, however, that connectional specificity, which is crucial for nerve signals to precisely reach their targets, is low at birth but improves as we develop and learn [97]. It is also important to note that connectional specificity is implicit in some textbook illustrations of the knee-jerk reflex and the withdrawal reflex (Fig 1A and 1B), but these illustrations are often so simple (without interneurons) that they compromise important aspects of reality and may lead to the misconception that neurons, in general, are connected one after another in a single chain. Such an understanding is incompatible with most, if not all behaviors, and is therefore a poor representation of nervous system structure–function relationships. Instead, connectional specificity should preferentially be demonstrated in a network which has a more realistic but comprehensible complexity, and which is involved in a more relevant behavior than mono- or disynaptic reflexes. Such networks can be found in [39, 40]. Connectional specificity is also missing from the brainfact-principles.

### When nerve signals travel through networks of neurons

Principle 15 describes the main purpose of nerve signals: they carry/contain information about the external environment and the body, which can be used by the individual, consciously or unconsciously, to generate a variety of behaviors as well as mental states like sensations, perceptions, thoughts and feelings. This knowledge is to some extent consistent with traditional content. However, different from many textbooks e.g. [73], the principle clearly links thoughts and feelings to the nervous system, and it specifies that these states are generated from the information carried by nerve signals. Including the word “information” implies that there is a process of interpretation going on in the network of neurons between the sensory signals and mental states. Thus, the particular mental state experienced by an individual depends on how the information is interpreted, which in turn depends on how the neurons are connected. This knowledge is essential in order to meet society’s current need for understanding the link between the body and mind [26]. Through this principle, students learn that nerve signals are more than just electrical and chemical pulses travelling in and out of some cells in the body. Nerve signals travelling in networks of neurons are the physical mechanisms underlying our perceptions, thoughts, and feelings, which are common components of the human mind [98]. The knowledge in the principle is consistent with the brainfact-principles.

One expert disagreed with this principle and argued that it is beyond our knowledge. In support of the expert, the causal relationship between nerve signals in neural networks of the human brain and conscious mental states is perhaps not yet sufficiently tested–one may argue that it is still at the correlational level. However, based on the responses from the other experts, and current literature on the topic [98–100] it does seem to reflect current understanding in the field.

Principle 16 specifies principle 15 by elaborating on the link between nerve signals and the mind. This elaboration is usually not included in traditional textbooks. Neither is it included in the brainfact-principles. However, it contributes important knowledge about mental and neurological disorders, which is needed in society [47]. Through this principle, students learn that the mental/neurological states (e. g. various feelings, thoughts and behaviors) we experience at any time depends on which network of neurons in the brain is activated, and the type of activity [99, 100], e. g. the temporal pattern of action potentials [55]. Consequently, disorders of the mental/neurological occur when the pattern of nerve signals and/or architecture of these networks change in specific ways [101, 102]. In contrast, traditional textbooks instead seem to explain mental/neurological disorders by neurotransmitters e.g. [73]. Although this is not wrong, it is a shortcut that masks the essential level of brain function which is the neural network level. Neurotransmitters themselves do not generate mental/neurological states. It is by acting through neurons, and influencing their generation of nerve signals, that neurotransmitters can be linked to mental/neurological states including disorders. The changes in pattern of nerve signals and/or architecture of neural networks may be caused by inherited factors, but also by experiences, including simple episodes of flashing lights which may trigger epileptic seizures [103], or complex episodes of warfare consciously perceived through the sense of vision, hearing, etc., which may trigger post-traumatic stress disorder [104]. Furthermore, this principle directly addresses a common misconception among teachers who believe that our feelings of happiness, anger and fear is experienced with our heart and not our brain [12, 19]. Thus, principle 16 provides a more concrete contribution than traditional textbook content, to counteract this misconception.

Two experts disagreed with this principle. One of them argued that it was weakly formulated (Expert E). However, based on the responses from the other experts, and current literature on the topic [98–100], it does seem to reflect current understanding in the field.

Principle 17 states that information from different sensory organs end up in separate regions of the brain. This is consistent with traditional content, and to some extent also with the brainfact-principles. It contributes to link the whole body to the brain, which is an essential step towards society’s need for understanding the link the body and the mind [26].

Principle 18 elaborates on principle 17 by pointing out that the anatomically and functionally separate regions of the brain are connected and functionally dependent on each other. This information is usually lacking in traditional textbooks. However, it provides an important first step to meet society’s need for understanding the interdependence of the emotional and the cognitive which has been emphasized by the OECD [26]. Through this principle, students learn that the brain works as an integrated unit as opposed to a collection of functionally independent regions. Each functional region performs just some of the computations usually needed for a particular behavior. Therefore, several regions must be involved to perform all computations for the complete behavior [105]. This is consistent with the brainfact-principles.

Principle 19 specifies principle 18 by pointing out that connections between brain regions/networks are reciprocal, and it exemplifies this with the networks of neurons responsible for our perceptions, emotions and behaviors. This information is lacking in traditional content as well as in the brainfact-principles. However, it contributes specific knowledge to meet society’s need for understanding the interdependence of the emotional and the cognitive [26]. Through this principle, students learn that information processing in the brain is not unidirectional from sensory to emotional to behavior, but rather bi- or multidirectional [106, 107]. This means, for example, that our emotions can be influenced, not only by what we perceive, but also by what we do or the way we behave [108]. Oppositely, it also means that our perception and behavior can be influenced by our emotions. Furthermore, since both perception and behavior involve an aspect of cognition, particularly in situations where language is involved, or reasoning, planning, decision-making, or learning and memory [26, 109], the principle also means that our emotions interact with our cognitive skills [110]. The principle can be exemplified by the discomfort (emotion) we feel when perceiving (cognition) other people in pain [111], or the strong memories (cognition) we often have from fearful experiences (emotion) compared to non-emotional experiences [112]. Other examples include our cognitive ability to imagine situations with the purpose of eliciting emotions, an exercise frequently practiced by professional actors to produce realistic bodily expressions consistent with the context of the play and the content of their lines [113]. Furthermore, emotions can feed back on our cognitive system to influence our future decisions (cognition) in a direction that depends on the feelings (emotions) we experienced after similar decisions (cognition) in the past [112]. As such, emotions give value to our cognitive processes, enhance memory formation, and this remembered value guide the direction of our future cognitive processes (e. g. our future decisions). This knowledge may help students understand why their education should include both cognitive and emotional training. The nervous system is constructed to make these properties interact. With proper training they may better support each other in our goal-directed behavior.

One expert disagreed with this principle and argued against using the word “feelings” (Expert E). In support of this expert, the word “feelings” is commonly used to described subjective states or inner experiences [114], and the interaction between such subjective states and perceptions or behaviors has perhaps not yet been demonstrated at the sufficient level of analysis. However, according to the OECD, feelings are described as a constituent of emotions [26]. Emotions, they claim, consists of 1) a particular mental state (feeling), 2) a particular physiological state (accelerated pulse, pallor, perspiring), and 3) an impulsion to behave in a particular way (facial expressions, freezing, fleeing, trembling). Therefore, as a constituent of the emotional network, one may argue that feelings interact with perceptions and behaviors. Based on the responses from the other experts, this seems to reflect current understanding in the field. However, pertinent stakeholders should be aware of this issue when using this principle.

Principle 20 introduces consciousness and describes some requirements necessary to become consciously aware of the information that comes through our senses. Importantly, these requirements are related to neural networks. This knowledge is missing from traditional content as well as from the brainfact-principles. However, it is important in order to meet society’s need for a better understanding of consciousness and free will [20]. Through this principle, students learn that we are not conscious of all the information that comes to our senses. In fact, most of the information that hits our senses never reaches our conscious mind [115], even if it travels quite far into the brain [116]. Furthermore, much of what the brain does with the information is also beyond our conscious awareness [117]. Therefore, we do not have complete consciously control of how sensory information will affect our mental states and behavior.

Principle 21 is an extension of principle 20 and points out the impact of unconscious sensory information on our mental/neurological state. This knowledge is also missing from traditional content as well as from the brainfact-principles, but it is important in order to meet society’s need for a better understanding of ourselves (self-conception) and the concept of free will [20]. Through this principle, students learn that sensory signals, although unconscious to them, can influence what they feel, think and do [118–120]. To what extent then, can we understand ourselves if we sometimes are unaware of the reasons for what we do? And to what extent is our will free if information of which we are unaware, can influence our behavior? Although empirical data seems to support the lack of free will [121], the topic is still debated [122]. Both these topics have, according to [20], already been successfully brought into lower secondary school through critical-reflective teaching of everyday myths about how neuroimaging and brain-computer interfaces allows for mind-reading and human manipulation. Another relevant context to discuss this principle is social media through which marketing has become an effective way of influencing people [123]. Students would perhaps benefit from knowing that the effect of unconsciously perceived sensory stimuli (subliminal priming) on human behavior is increasingly being used in political campaigns, product promotion and marketing across many areas of public interest to influence voters and consumers [124] beyond their awareness, and young people are particularly susceptible [125]. Consequently, introducing this principle in compulsory education is becoming more important since current technology is increasingly designed to impose consumer addiction by targeting the brain’s emotional and reward systems [126, 127]. In addition, our culture is increasingly allowing children of young age to use such technology.

### Plasticity in the nervous system

Principle 22 elaborates on the principles under theme four by describing synaptic plasticity. This information is sometimes lacking or minimally present in traditional content. However, since synapses are the target of many drugs, and addiction results from drug-induced changes of synapses in the brain [128] to the extent that our ability to execute “free” will is impaired [129], the principle is important in order to meet society’s need for a better understanding of substance use disorders [29, 47] and free will [20]. Considering the global increase in drug abuse and the young age at which people typically have their first experience [130], it may be wise to bring this knowledge into early education. The information is included in the brainfact-principles.

Principle 23 specifies principle 22 by describing the functional implications of a synaptic change at the level of neural networks. This information is usually lacking or minimally present in traditional content, but it contributes to meet society’s need for a better understanding of substance use disorders [29, 47] and free will [20] by describing how a synaptic change may alter the activity of a neural networks.

Principle 24 link synaptic plasticity to learning and memory. This information is usually lacking in traditional content e.g. [84, 94] and it is also missing from the brainfact-principles. However, this information contributes to meet our society’s need for knowledge about learning and memory [26]. Importantly, the principle includes that learning may weaken synapses, not only strengthen them. This may sound counterintuitive, but it makes sense if one knows that the number of synapses in babies is much higher than in adults [131], and that their locations are less accurate [97]. It makes even more sense if you acknowledge that learning, for example a motor task, which usually involves improving precision and accuracy of movements [132], is not about having many connections, but the right connections with the proper strength.

Principle 25 introduces the health benefits of learning even at old age. This knowledge is usually lacking in traditional content. However, it is important in order to meet our society’s need for knowledge about the health benefits of learning [26]. Through this principle, students may realize that learning is not only granted the young, and it does not end with basic schooling. Learning can and should take place throughout life. The principle can be used to moderate a common misconception which holds that all important aspects of brain function are determined by the age of three [26]. The information is partly present in the brainfact-principles.

Principle 26 describes the difference in plasticity between brain regions over the lifetime. This knowledge is usually lacking in traditional content, and it is absent from the brainfact-principles. However, it can contribute to the society’s need for a better understanding of adolescence mental life and behavior [26]. For example, the high cognitive potential and emotional immaturity of adolescents [133], may be due to the inevitable biology of brain development which proceed in the posterior-to-anterior direction [131, 134, 135]. The result of this sequential development is a well-developed sensory and motor region, which promote cognitive potential, and a mature hypothalamus which triggers a behavioral drive through a surge of hormones. This happens while the prefrontal cortex, which plays a central role in behavioral control [136], is still underdeveloped. Including this knowledge in secondary school is in line with Blakemore [48] and MacNabb [49], who argues that adolescents might be interested in, and could benefit from, learning about the changes that are going on in their own brains. The principle can also be used to argue against a common misconception which holds that there are critical periods in life when certain matters must be taught and learnt [26]. In line with our principle and [26], there are no “critical periods” when learning *must* take place but there are “sensitive periods” when the individual is particularly primed to engage in specific learning activities [137]. The difference may seem insignificant, but it has severe consequences for the stress levels of parents whos’ children haven’t yet acquired the expected skills within the presumed critical time window. Replacing the old understanding of certain developmental periods as “critical” with our current understanding of these periods as “sensitive”, makes people realize that there is hope, in most cases, even beyond certain periods of development.

### Limitations of the study

Due to the strong consistency between our principles and the different needs for knowledge about the nervous system in society, as expressed by relevant global organizations (OECD and WHO), scientific communities (neuroscience, social sciences and humanity), political documents (curricula), and research (e. g. misconceptions), we believe that the results from this study are generalizable across the globe. Nevertheless, there are at least three issues that may question the validity of the principles. One issue concerns the distribution of experts among areas of expertise. This shows a clear overweight of experts within the area of systems neuroscience which may have contributed to a disproportionate emphasis on certain aspects of the nervous system. This could have been improved to some extent by inviting an equal number of experts within each field. However, finding such an ideal distribution of experts willing to participate in such a study, is a challenging task.

A second issue concerns the distribution of nationalities among the experts in the study. The overweight of experts from Norway may also have contributed to a disproportionate emphasis on certain aspects of the nervous system. However, the initial part of the study, where the premade statements were generated from international textbooks, would likely contribute to reduce such a potential effect.

A third issue is related to the number of experts completing all four rounds. A reduction from fifteen to five experts from first to third round is substantial. However, because most of them (11 of 15) responded in the fourth and last round, and since the reason for dropping out in between rounds seemed to be lack of time rather than disagreement with the principles or the method leading to them, this issue may not be very significant.

### Implications and future prospects

The importance of knowledge about the nervous system has been emphasized throughout this paper, and the diversity and complexity of the topic are major obstacles to public understanding. By developing the list of 26 principles in this study and discussing how they comply with the needs of society, we hope they are rendered applicable to pertinent stakeholders who needs to develop the biology curriculum by for example writing policy documents or textbooks, or more practically by teaching about the nervous system. Curricula developers might find them useful for selection and formulation of learning goals. Authors might use them as a check list for textbook content, and teachers could, for example, use them as a guide to select classroom content. Even though many principles have been contextualized in the discussion, further studies are needed to explore how the principles could be best introduced in the classroom as well as at what level the different principles are most appropriate. Currently we suggest that they are viewed as curriculum principles that need to be adapted to the goals and context of the education in question. As mentioned earlier, there may be several views on what content to include in the curriculum and to teach in the classroom, and they may all have their rationale, even if they are conflicting. The present study, however, represents the expert view. This view provides an important voice to the content debate and curricula development because it has seemingly no other agenda than to promote an understanding of the nervous system. In addition, these curriculum themes and principles could be used as the foundation in the further development of a new learning progression in neuroscience education.

## Conclusion

This study has reached a high level of consensus among a selected group of fifteen experts, on six knowledge themes and 26 content principles considered important to teach about the nervous system in the context of secondary biology and science education. The principles are consistent with the official steering documents of secondary education in several countries, but also expand and specify the propositions of the steering documents, thus making them useful. Some of the principles clarify and elaborate on traditional content, whereas others add new knowledge. Importantly, the new knowledge meets the needs of society, as expressed by recent international policy frameworks of OECD and WHO, by providing fundamental knowledge underlying contemporary topics like 1) the interdependence of body and mind, 2) learning as a lifelong activity with health benefits, 3) adolescent mental life and behavior, 4) consciousness and free will, as well as 5) mental, neurological and substance use disorders. In addition, seven principles address four common misconceptions about the brain. The study suggests an update of classroom content, and it provides the means to do so: a list of specifications to current curricula which can be used to guide classroom instruction, textbook content, and curricula goals.

## Supporting information

S1 Table Categorization of content in textbook one and two. The table shows the categorization of the different parts and chapters within the neuroscience textbooks “Principles of Neural Science” by Kandel et al. (2013), and “Fundamental Neuroscience” by Squire et al. (2012) into major neuroscience topics.
(DOCX)

S2 Table Categorization of content in textbook three and four. The table shows the categorization of the different parts and chapters within the neuroscience textbooks “Neuroscience: Exploring the brain” by Bear et al. (2016), and “Neuroscience” by Purves et al. (2018) into major neuroscience topics.
(DOCX)

S3 Table Citations collected from the textbook “principles of neural science”. The table shows all citations collected from the textbook “Principles of neural science” [37], as well as the chapters they were collected from, the major neuroscience topic categories they were ascribed to, and whether they were kept and used to develop the statements, or discarded. The citations which were kept are denoted with the number of the specific statements they were used in, and the citations which were discarded are denoted with the reason why they were discarded.
(DOCX)

S4 Table The development of premade statements. The table shows the final list of premade statements (column 2), the major neuroscientific topic categories they were ascribed to (column 3), and the textbook citations (column 4) with citation number (column 3) which were used to develop them.
(DOCX)

S5 Table Responses from experts in all rounds. The table shows an overview of all responses from all experts to each of the statements in all rounds. The letters denote different experts, but each letter does not necessarily denote the same experts across rounds.
(DOCX)
