# Supplementary material for: Identifying knowledge important to teach about the nervous system in the context of secondary biology and science education–A Delphi study
Source: PLoS One. 2021 Dec 21;16(12):e0260752. doi: 10.1371/journal.pone.0260752 (PMC8691623; doi:10.1371/journal.pone.0260752)
Supplement: S1 Table — The table shows the categorization of the different parts and chapters within the neuroscience textbooks “Principles of Neural Science” by Kandel et al. (2013), and “Fundamental Neuroscience” by Squire et al. (2012) into major neuroscience topics. (DOCX) [file pone.0260752.s001.docx]

**S1 Table. Categorization of content in textbook one and two.** The table shows the categorization of the different parts and chapters within the neuroscience textbooks “Principles of Neural Science” by Kandel et al. (2013), and “Fundamental Neuroscience” by Squire et al. (2012) into major neuroscience topics.

| **Short content** | | | | |
| --- | --- | --- | --- | --- |
| **Part nr.** | **Name of the different parts in the textbook “Principles of neural science” [37]** | **Categorization of the parts in the textbook “Principles of neural science” [37]** | **Name of the different parts in the textbook “Fundamental neuroscience” [65]** | **Categorization of the parts in the textbook “Fundamental neuroscience” [65]** |
| 1 | Overall perspective |  | Fundamental neuroscience |  |
| 2 | Cell and molecular biology of the neuron | Cell structure and function | Cellular and molecular neuroscience | Cell structure and function |
| 3 | Synaptic transmission | Cell structure and function | Nervous system development | Plasticity |
| 4 | The neural basis of cognition | Cognition and other complex brain functions | Sensory systems | Sensory systems |
| 5 | Perception | Sensory systems | Motor systems | Motor systems |
| 6 | Movement | Motor systems | Regulatory systems | Cognition and other complex brain functions |
| 7 | The unconscious and conscious processing of neural information | Cognition and other complex brain functions | Behavioral and cognitive neuroscience | Cognition and other complex brain functions |
| 8 | Development and the emergency of behavior | Plasticity |  |  |
| 9 | Language, thought, affect, and learning | Cognition and other complex brain functions |  |  |
|  | | | | |
| **Expanded contents** | | | | |
| **Chapter nr.** | **Name of the chapters in the textbook “Principles of neural science” [37]** | **Categorization of the chapters in the textbook “Principles of neural science” [37]** | **Name of the chapters in the textbook “Fundamental neuroscience” [65]** | **Categorization of the chapters in the textbook “Fundamental neuroscience” [65]** |
| 1 | The Brain and Behavior | Structure/organization of the nervous system  Cognition and other complex brain functions | Fundamentals of neuroscience | Structure/organization of the nervous system  Cell structure and function |
| 2 | Nerve Cells, Neural Circuitry, and Behavior | Cell structure and function | Basic plan of the nervous system | Structure/organization of the nervous system |
| 3 | [Genes and Behavior](https://neurology.mhmedical.com/content.aspx?bookid=1049&sectionid=59138623) | Genes and behavior | Cellular components of nervous tissue | Cell structure and function |
| 4 | [The Cells of the Nervous System](https://neurology.mhmedical.com/content.aspx?bookid=1049&sectionid=59138626) | Cell structure and function | Subcellular organization of the nervous system: Organelles and their functions | Cell structure and function |
| 5 | [Ion Channels](https://neurology.mhmedical.com/content.aspx?bookid=1049&sectionid=59138627) | Cell structure and function | Membrane potential and action potential | Cell structure and function |
| 6 | [Membrane Potential and the Passive Electrical Properties of the Neuron](https://neurology.mhmedical.com/content.aspx?bookid=1049&sectionid=59138628) | Cell structure and function | Neurotransmitters | Cell structure and function |
| 7 | [Propagated Signaling: The Action Potential](https://neurology.mhmedical.com/content.aspx?bookid=1049&sectionid=59138629) | Cell structure and function | Release of neurotransmitters | Cell structure and function |
| 8 | [Overview of Synaptic Transmission](https://neurology.mhmedical.com/content.aspx?bookid=1049&sectionid=59138632) | Cell structure and function | Transmitter receptors | Cell structure and function |
| 9 | [Signaling at the Nerve-Muscle Synapse: Directly Gated Transmission](https://neurology.mhmedical.com/content.aspx?bookid=1049&sectionid=59138633) | Cell structure and function | Intracellular signaling | Cell structure and function |
| 10 | [Synaptic Integration in the Central Nervous System](https://neurology.mhmedical.com/content.aspx?bookid=1049&sectionid=59138634) | Cell structure and function | Postsynaptic potentials and synaptic integration | Cell structure and function |
| 11 | [Modulation of Synaptic Transmission: Second Messengers](https://neurology.mhmedical.com/content.aspx?bookid=1049&sectionid=59138635) | Cell structure and function | Information processing in dendrites and spines | Cell structure and function |
| 12 | [Transmitter Release](https://neurology.mhmedical.com/content.aspx?bookid=1049&sectionid=59138636) | Cell structure and function | Brain energy metabolism | Brain energy metabolism |
| 13 | [Neurotransmitters](https://neurology.mhmedical.com/content.aspx?bookid=1049&sectionid=59138637) | Cell structure and function | Neural induction and pattern formation | Plasticity |
| 14 | [Diseases of the Nerve and Motor Unit](https://neurology.mhmedical.com/content.aspx?bookid=1049&sectionid=59138638) | Motor systems  Cell structure and function | Cellular determination | Plasticity |
| 15 | [The Organization of the Central Nervous System](https://neurology.mhmedical.com/content.aspx?bookid=1049&sectionid=59138641) | Structure/organization of the nervous system | Neurogenesis and migration | Plasticity |
| 16 | [The Functional Organization of Perception and Movement](https://neurology.mhmedical.com/content.aspx?bookid=1049&sectionid=59138642) | Structure/organization of the nervous system  Sensory systems  Motor systems | Growth cones and axon pathfinding | Plasticity |
| 17 | [From Nerve Cells to Cognition: The Internal Representations of Space and Action](https://neurology.mhmedical.com/content.aspx?bookid=1049&sectionid=59138643) | Cognition and other complex brain functions | Synapse formation | Plasticity |
| 18 | [The Organization of Cognition](https://neurology.mhmedical.com/content.aspx?bookid=1049&sectionid=59138644) | Cognition and other complex brain functions | Programmed cell death and neurotrophic factors | Plasticity |
| 19 | [Cognitive Functions of the Premotor Systems](https://neurology.mhmedical.com/content.aspx?bookid=1049&sectionid=59138645) | Cognition and other complex brain functions | Synapse elimination | Plasticity |
| 20 | [Functional Imaging of Cognition](https://neurology.mhmedical.com/content.aspx?bookid=1049&sectionid=59138646) | Cognition and other complex brain functions | Dendritic development | Plasticity |
| 21 | [Sensory Coding](https://neurology.mhmedical.com/content.aspx?bookid=1049&sectionid=59138649) | Sensory systems | Early experience and sensitive periods | Plasticity |
| 22 | [The Somatosensory System: Receptors and Central Pathways](https://neurology.mhmedical.com/content.aspx?bookid=1049&sectionid=59138650) | Sensory systems | Fundamentals of sensory systems | Sensory systems |
| 23 | [Touch](https://neurology.mhmedical.com/content.aspx?bookid=1049&sectionid=59138651) | Sensory systems | Chemical senses: Taste and olfaction | Sensory systems |
| 24 | [Pain](https://neurology.mhmedical.com/content.aspx?bookid=1049&sectionid=59138652) | Sensory systems | The somatosensory system | Sensory systems |
| 25 | [The Constructive Nature of Visual Processing](https://neurology.mhmedical.com/content.aspx?bookid=1049&sectionid=59138653) | Sensory systems | Audition | Sensory systems |
| 26 | [Low-Level Visual Processing: The Retina](https://neurology.mhmedical.com/content.aspx?bookid=1049&sectionid=59138654) | Sensory systems | Vision | Sensory systems |
| 27 | [Intermediate-Level Visual Processing and Visual Primitives](https://neurology.mhmedical.com/content.aspx?bookid=1049&sectionid=59138655) | Sensory systems | Fundamentals of motor systems | Motor systems |
| 28 | [High-Level Visual Processing: Cognitive Influences](https://neurology.mhmedical.com/content.aspx?bookid=1049&sectionid=59138656) | Sensory systems  Cognition and other complex brain functions | The spinal and peripheral motor system | Motor systems |
| 29 | [Visual Processing and Action](https://neurology.mhmedical.com/content.aspx?bookid=1049&sectionid=59138657) | Sensory systems  Motor systems | Control of movement | Motor systems |
| 30 | [The Inner Ear](https://neurology.mhmedical.com/content.aspx?bookid=1049&sectionid=59138658) | Sensory systems | The basal ganglia | Motor systems |
| 31 | [The Auditory Central Nervous System](https://neurology.mhmedical.com/content.aspx?bookid=1049&sectionid=59138659) | Sensory systems | Cerebellum | Motor systems |
| 32 | [Smell and Taste: The Chemical Senses](https://neurology.mhmedical.com/content.aspx?bookid=1049&sectionid=59138660) | Sensory systems | Eye movements | Motor systems |
| 33 | [The Organization and Planning of Movement](https://neurology.mhmedical.com/content.aspx?bookid=1049&sectionid=59138663) | Structure/organization of the nervous system  Motor systems | The hypothalamus: An overview of regulatory systems | Cognition and other complex brain functions |
| 34 | [The Motor Unit and Muscle Action](https://neurology.mhmedical.com/content.aspx?bookid=1049&sectionid=59138664) | Motor systems | Central control of autonomic functions: Organization of the autonomic nervous system | Cognition and other complex brain functions |
| 35 | [Spinal Reflexes](https://neurology.mhmedical.com/content.aspx?bookid=1049&sectionid=59138665) | Sensory system  Motor system | Neural control of respiratory and cardiovascular functions | Cognition and other complex brain functions |
| 36 | [Locomotion](https://neurology.mhmedical.com/content.aspx?bookid=1049&sectionid=59138666) | Motor systems | Food intake and metabolism | Cognition and other complex brain functions |
| 37 | [Voluntary Movement: The Primary Motor Cortex](https://neurology.mhmedical.com/content.aspx?bookid=1049&sectionid=59138667) | Motor systems | Water and salt intake and body fluid homeostasis | Cognition and other complex brain functions |
| 38 | [Voluntary Movement: The Parietal and Premotor Cortex](https://neurology.mhmedical.com/content.aspx?bookid=1049&sectionid=59138668) | Motor systems | Neuroendocrine systems | Cognition and other complex brain functions |
| 39 | [The Control of Gaze](https://neurology.mhmedical.com/content.aspx?bookid=1049&sectionid=59138669) | Motor systems | Circadian timekeeping | Cognition and other complex brain functions |
| 40 | [The Vestibular System](https://neurology.mhmedical.com/content.aspx?bookid=1049&sectionid=59138670) | Sensory system  Motor system | The neurobiology of sleep and dreaming | Cognition and other complex brain functions |
| 41 | [Posture](https://neurology.mhmedical.com/content.aspx?bookid=1049&sectionid=59138671) | Sensory system  Motor system | Reward, motivation and addiction | Cognition and other complex brain functions |
| 42 | [The Cerebellum](https://neurology.mhmedical.com/content.aspx?bookid=1049&sectionid=59138672) | Sensory system  Motor system | Human brain evolution | Plasticity |
| 43 | [The Basal Ganglia](https://neurology.mhmedical.com/content.aspx?bookid=1049&sectionid=59138673) | Motor systems | Cognitive development and aging | Cognition and other complex brain functions |
| 44 | [Genetic Mechanisms in Degenerative Diseases of the Nervous System](https://neurology.mhmedical.com/content.aspx?bookid=1049&sectionid=59138674) | Disorder | Visual perception of objects | Sensory systems |
| 45 | [The Sensory, Motor, and Reflex Functions of the Brain Stem](https://neurology.mhmedical.com/content.aspx?bookid=1049&sectionid=59138677) | Sensory system  Motor system | Spatial cognition | Cognition and other complex brain functions |
| 46 | [The Modulatory Functions of the Brain Stem](https://neurology.mhmedical.com/content.aspx?bookid=1049&sectionid=59138678) | Cognition and other complex brain functions | Attention | Cognition and other complex brain functions |
| 47 | [The Autonomic Motor System and the Hypothalamus](https://neurology.mhmedical.com/content.aspx?bookid=1049&sectionid=59138679) | Cognition and other complex brain functions | Learning and memory: Basic mechanisms | Plasticity |
| 48 | [Emotions and Feelings](https://neurology.mhmedical.com/content.aspx?bookid=1049&sectionid=59138680) | Cognition and other complex brain functions | Learning and memory: brain systems | Plasticity |
| 49 | [Homeostasis, Motivation, and Addictive States](https://neurology.mhmedical.com/content.aspx?bookid=1049&sectionid=59138681) | Cognition and other complex brain functions | Language | Cognition and other complex brain functions |
| 50 | [Seizures and Epilepsy](https://neurology.mhmedical.com/content.aspx?bookid=1049&sectionid=59138682) | Disorder | The prefrontal cortex and executive brain functions | Cognition and other complex brain functions |
| 51 | [Sleep and Dreaming](https://neurology.mhmedical.com/content.aspx?bookid=1049&sectionid=59138683) | Cognition and other complex brain functions | The neuroscience of consciousness | Cognition and other complex brain functions |
| 52 | [Patterning the Nervous System](https://neurology.mhmedical.com/content.aspx?bookid=1049&sectionid=59138686) | Plasticity |  |  |
| 53 | [Differentiation and Survival of Nerve Cells](https://neurology.mhmedical.com/content.aspx?bookid=1049&sectionid=59138687) | Plasticity |  |  |
| 54 | [The Growth and Guidance of Axons](https://neurology.mhmedical.com/content.aspx?bookid=1049&sectionid=59138688) | Plasticity |  |  |
| 55 | [Formation and Elimination of Synapses](https://neurology.mhmedical.com/content.aspx?bookid=1049&sectionid=59138689) | Plasticity |  |  |
| 56 | [Experience and the Refinement of Synaptic Connections](https://neurology.mhmedical.com/content.aspx?bookid=1049&sectionid=59138690) | Plasticity |  |  |
| 57 | [Repairing the Damaged Brain](https://neurology.mhmedical.com/content.aspx?bookid=1049&sectionid=59138691) | Plasticity |  |  |
| 58 | [Sexual Differentiation of the Nervous System](https://neurology.mhmedical.com/content.aspx?bookid=1049&sectionid=59138692) | Plasticity |  |  |
| 59 | [The Aging Brain](https://neurology.mhmedical.com/content.aspx?bookid=1049&sectionid=59138693) | Plasticity |  |  |
| 60 | [Language](https://neurology.mhmedical.com/content.aspx?bookid=1049&sectionid=59138696) | Cognition and other complex brain functions |  |  |
| 61 | [Disorders of Conscious and Unconscious Mental Processes](https://neurology.mhmedical.com/content.aspx?bookid=1049&sectionid=59138697) | Cognition and other complex brain functions |  |  |
| 62 | [Disorders of Thought and Volition: Schizophrenia](https://neurology.mhmedical.com/content.aspx?bookid=1049&sectionid=59138698) | Cognition and other complex brain functions |  |  |
| 63 | [Disorders of Mood and Anxiety](https://neurology.mhmedical.com/content.aspx?bookid=1049&sectionid=59138699) | Cognition and other complex brain functions |  |  |
| 64 | [Autism and Other Neurodevelopmental Disorders Affecting Cognition](https://neurology.mhmedical.com/content.aspx?bookid=1049&sectionid=59138700) | Cognition and other complex brain functions |  |  |
| 65 | [Learning and Memory](https://neurology.mhmedical.com/content.aspx?bookid=1049&sectionid=59138701) | Plasticity |  |  |
| 66 | [Cellular Mechanisms of Implicit Memory Storage and the Biological Basis of Individuality](https://neurology.mhmedical.com/content.aspx?bookid=1049&sectionid=59138702) | Plasticity |  |  |
| 67 | [Prefrontal Cortex, Hippocampus, and the Biology of Explicit Memory Storage](https://neurology.mhmedical.com/content.aspx?bookid=1049&sectionid=59138703) | Plasticity |  |  |
