# Supplementary material for: Identifying knowledge important to teach about the nervous system in the context of secondary biology and science education–A Delphi study
Source: PLoS One. 2021 Dec 21;16(12):e0260752. doi: 10.1371/journal.pone.0260752 (PMC8691623; doi:10.1371/journal.pone.0260752)
Supplement: S2 Table — The table shows the categorization of the different parts and chapters within the neuroscience textbooks “Neuroscience: Exploring the brain” by Bear et al. (2016), and “Neuroscience” by Purves et al. (2018) into major neuroscience topics. (DOCX) [file pone.0260752.s002.docx]

**S2 Table. Categorization of content in textbook three and four.** The table shows the categorization of the different parts and chapters within the neuroscience textbooks “Neuroscience: Exploring the brain” by Bear et al. (2016), and “Neuroscience” by Purves et al. (2018) into major neuroscience topics.

| **Short content** | | | | |
| --- | --- | --- | --- | --- |
| **Part nr.** | **Name of the different parts in the textbook “Neuroscience: Exploring the brain” [67]** | **Categorization of the parts in the textbook “Neuroscience: Exploring the brain” [67]** | **Name of the different parts in the textbook “Neuroscience” [66]** | **Categorization of the parts in the textbook “Neuroscience” [66]** |
| 1 | Foundations | Structure/organization of the nervous system  Cell structure and function | Studying the nervous system | Structure/organization of the nervous system  Cell structure and function |
| 2 | Sensory and motor systems | Sensory systems  Motor systems | Neural Signaling | Cell structure and function |
| 3 | The brain and behavior | Cognition and other complex brain functions  Motor systems | Sensation and Sensory Processing | Sensory systems |
| 4 | The changing brain | Plasticity | Movement and Its Central Control | Motor systems |
|  |  |  | The Changing Brain | Plasticity |
|  |  |  | Complex Brain Functions and Cognitive Neuroscience | Cognition and other complex brain functions |
|  | | | | |
| **Expanded contents** | | | | |
| **Chapter nr.** | **Name of the chapters in the textbook “Neuroscience: Exploring the brain” [67]** | **Categorization of the chapters in the textbook “Neuroscience: Exploring the brain” [67]** | **Name of the chapters in the textbook “Neuroscience” [66]** | **Categorization of the chapters in the textbook “Neuroscience” [66]** |
| 1 | Neuroscience: Past, present and future | Structure/organization of the nervous system | Studying the Nervous System | Structure/organization of the nervous system  Cell structure and function |
| 2 | Neurons and glia | Cell structure and function | Electrical Signals of Nerve Cells | Cell structure and function |
| 3 | The neuronal membrane at rest | Cell structure and function | Voltage-Dependent Membrane Permeability | Cell structure and function |
| 4 | The action potential | Cell structure and function | Ion Channels and Transporters | Cell structure and function |
| 5 | Synaptic transmission | Cell structure and function | Synaptic Transmission | Cell structure and function |
| 6 | Neurotransmitter systems | Cell structure and function | Neurotransmitters and Their Receptors | Cell structure and function |
| 7 | The structure of the nervous system | Structure/organization of the nervous system | Molecular Signaling within Neurons | Cell structure and function |
| 8 | The chemical senses | Sensory systems | Synaptic Plasticity | Plasticity |
| 9 | The eye | Sensory systems | The Somatosensory System: Touch and Proprioception | Sensory systems |
| 10 | The central visual system | Sensory systems | Pain | Sensory systems |
| 11 | The auditory and vestibular systems | Sensory systems | Vision: The Eye | Sensory systems |
| 12 | The somatic sensory system | Sensory systems | Central Visual Pathways | Sensory systems |
| 13 | Spinal control of movement | Motor systems | The Auditory System | Sensory systems |
| 14 | Brain control of movement | Motor systems | The Vestibular System | Sensory systems |
| 15 | Chemical control of the brain and behavior | Cognition and other complex brain functions | The Chemical Senses | Sensory systems |
| 16 | Motivation | Cognition and other complex brain functions | Lower Motor Neuron Circuits and Motor Control | Motor systems |
| 17 | Sex and the brain | Cognition and other complex brain functions | Upper Motor Neuron Control of the Brainstem and Spinal Cord | Motor systems |
| 18 | Brain mechanism of emotion | Cognition and other complex brain functions | Modulation of Movement by the Basal Ganglia | Motor systems |
| 19 | Brain rhythms and sleep | Cognition and other complex brain functions | Modulation of Movement by the Cerebellum | Motor systems |
| 20 | Language | Cognition and other complex brain functions | Eye Movements and Sensory Motor Integration | Motor systems  Sensory systems |
| 21 | Attention | Cognition and other complex brain functions | The Visceral Motor System | Motor systems |
| 22 | Mental illness | Cognition and other complex brain functions  Disorder | Early Brain Development | Plasticity |
| 23 | Wiring the brain | Plasticity | Construction of Neural Circuits | Plasticity |
| 24 | Memory systems | Plasticity | Circuit Differentiation: Intrinsic Factors and Sex Differences | Plasticity |
| 25 | Molecular mechanisms of learning and memory | Plasticity | Experience-Dependent Plasticity in the Developing Brain | Plasticity |
| 26 |  |  | Repair and Regeneration in the Nervous System | Plasticity |
| 27 |  |  | Cognitive Functions and the Organization of the Cerebral Cortex | Cognition and other complex brain functions |
| 28 |  |  | Cortical States | Cognition and other complex brain functions |
| 29 |  |  | Attention | Cognition and other complex brain functions |
| 30 |  |  | Memory | Cognition and other complex brain functions  Plasticity |
| 31 |  |  | Emotion | Cognition and other complex brain functions |
| 32 |  |  | Thinking, Planning, and Deciding | Cognition and other complex brain functions |
| 33 |  |  | Speech and Language | Cognition and other complex brain functions |
| 34 |  |  | Development and Evolution of Cognitive Functions | Plasticity |
