# Supplementary material for: Identifying knowledge important to teach about the nervous system in the context of secondary biology and science education–A Delphi study
Source: PLoS One. 2021 Dec 21;16(12):e0260752. doi: 10.1371/journal.pone.0260752 (PMC8691623; doi:10.1371/journal.pone.0260752)
Supplement: S3 Table — The table shows all citations collected from the textbook “Principles of neural science” [37], as well as the chapters they were collected from, the major neuroscience topic categories they were ascribed to, and whether they were kept and used to develop the statements, or discarded. The citations which were kept are denoted with the number of the specific statements they were used in, and the citations which were discarded are denoted with the reason why they were discarded. (DOCX) [file pone.0260752.s003.docx]

**S3 Table. Citations collected from the textbook “Principles of neural science”.**

The table shows all citations collected from the textbook “Principles of neural science” [37], as well as the chapters they were collected from, the major neuroscience topic categories they were ascribed to, and whether they were kept and used to develop the statements, or discarded. The citations which were kept are denoted with the number of the specific statements they were used in, and the citations which were discarded are denoted with the reason why they were discarded.

| **Chapter nr.** | **Chapter name in the textbook “Principles of neural science” [37]** | **Citation nr.** | **Citations from the textbook “Principles of neural science” [37]** | **Major neuroscience topic categories** | **Kept or discarded** |
| --- | --- | --- | --- | --- | --- |
| 1 | [The Brain and Behavior](https://neurology.mhmedical.com/content.aspx?bookid=1049&sectionid=59138621) | 1 | One of the chief ideas we shall develop in this book is that the specificity of the synaptic connections established during development underlie perceptions, action, emotion, and learning. (p. 4). | Structure/organization of the nervous system | Kept (used to develop statements 18, 14) |
|  |  |  |  | Cognition and other complex brain functions |  |
|  |  | 2 | Using Golgi's technique, Ramón y Cajal discovered that nervous tissue is not a syncytium, a continuous web of elements, but a network of discrete cells. In the course of this work Ramón y Cajal developed some of the key concepts and much of the early evidence for the *neuron doctrine –* the principle that individual neurons are the elementary building blocks and signaling elements of the nervous system (p. 6). | Structure/organization of the nervous system | Kept  (used to develop statement 1) |
|  |  |  |  | Cell structure and function |  |
|  |  | 3 | In placing the principle of localized function within a connectionist framework, Wernicke realized that different components of a single behavior are likely to be processed in several regions of the brain. He was thus the first to advance the idea of *distributed processing,* now a central tenet of neural science (p. 12). | Structure/organization of the nervous system | Kept  (used to develop statement 21) |
|  |  |  |  | Cognition and other complex brain functions |  |
|  |  | 4 | As we shall see in later chapters, functional specialization is a key organizing principle in the cerebral cortex, extending even to individual columns of cells within a functional area (p. 13). | Structure/organization of the nervous system | Kept  (used to develop statement 21) |
|  |  |  |  | Cognition and other complex brain functions |  |
| 2 | [Nerve Cells, Neural Circuitry, and Behavior](https://neurology.mhmedical.com/content.aspx?bookid=1049&sectionid=59138622) | 5 | Here we see a key principle of brain function: the information conveyed by an action potential is determined not by the form of the signal but by the pathway the signal travels in the brain. The brain analyzes and interprets patterns of incoming electrical signals and their pathways, and in turn creates our sensations of sight, touch, smell, and sound (p. 23). | Structure/organization of the nervous system | Kept  (used to develop statements 6, 18, 19) |
|  |  |  |  | Cell structure and function |  |
|  |  |  |  | Sensory systems |  |
|  |  |  |  | Cognition and other complex brain functions |  |
|  |  | 6 | The principle that each neuron is a discrete cell with distinctive processes arising from its cell body and that neurons are the signaling units of the nervous system (p. 23). | Cell structure and function | Kept  (used to develop statement 2) |
|  |  | 7 | The first of these has come to be known as the *principle of dynamic polarization.* It states that electrical signals within a nerve cell flow only in one direction: from the receiving sites of the neuron, usually the dendrites and cell body, to the trigger region at the axon (p. 24). | Cell structure and function | Kept  (used to develop statement 7) |
|  |  | 8 | The other principle advanced by Ramón y Cajal is that of *connectional specificity,* which states that nerve cells do not connect randomly with one another in the formation of networks. Rather each cell makes specific connections at particular contact points with certain postsynaptic target cells but not with others (p. 24). | Cell structure and function | Kept  (used to develop statements 17, and 11) |
|  |  |  |  | Structure/organization of the nervous system |  |
|  |  | 9 | “Regardless of cell size and shape, transmitter biochemistry, or behavioral function, almost all neurons can be described by a model neuron that has four functional components that generate the four types of signals: a receptive component, a summing or integrative component, a long-range signaling component, and a secretory component (Figure 2-9). This model neuron is the physiological expression of Ramón y Cajal's principle of dynamic polarization (p. 30). | Cell structure and function | Kept  (used to develop statement 5 and 11) |
| 3 | [Genes and Behavior](https://neurology.mhmedical.com/content.aspx?bookid=1049&sectionid=59138623) |  |  |  |  |
| 4 | [The Cells of the Nervous System](https://neurology.mhmedical.com/content.aspx?bookid=1049&sectionid=59138626) |  |  |  |  |
| 5 | [Ion Channels](https://neurology.mhmedical.com/content.aspx?bookid=1049&sectionid=59138627) |  |  |  |  |
| 6 | [Membrane Potential and the Passive Electrical Properties of the Neuron](https://neurology.mhmedical.com/content.aspx?bookid=1049&sectionid=59138628) |  |  |  |  |
| 7 | [Propagated Signaling: The Action Potential](https://neurology.mhmedical.com/content.aspx?bookid=1049&sectionid=59138629) |  |  |  |  |
| 8 | [Overview of Synaptic Transmission](https://neurology.mhmedical.com/content.aspx?bookid=1049&sectionid=59138632) | 10 | , … all neurons make use of one of the two basic forms of synaptic transmission: electrical or chemical (p. 177). | Cell structure and function | Kept  (used to develop statement 10) |
|  |  | 11 | “Because chemical synaptic transmission is so central to understanding brain and behavior, it is examined in detail in the next four chapters”… “They can mediate either excitatory or inhibitory actions in postsynaptic cells and produce electrical changes in the postsynaptic cell that last from milliseconds to many minutes” (p. 177). | Cell structure and function | Kept  (used to develop statement 12) |
| 9 | [Signaling at the Nerve-Muscle Synapse: Directly Gated Transmission](https://neurology.mhmedical.com/content.aspx?bookid=1049&sectionid=59138633) |  |  |  |  |
| 10 | [Synaptic Integration in the Central Nervous System](https://neurology.mhmedical.com/content.aspx?bookid=1049&sectionid=59138634) | 12 | “Thus, many principles that apply to the synaptic connection between the motor neuron and skeletal muscle fiber at the neuromuscular junction also apply in the central nervous system. Synaptic transmission between central neurons is more complex, however, for several reasons. First, … Second, muscle fibers receive only excitatory inputs, whereas central neurons receive both excitatory and inhibitory inputs” (p. 210). | Structure/organization of the nervous system | Kept  (used to develop statements 13 and 12) |
|  |  | 13 | “Excitatory and Inhibitory Synaptic Actions Are Integrated by the Cell into a Single Output” (p. 210). | Cell structure and function | Kept  (used to develop statement 13) |
|  |  | 14 | “Thus, many principles that apply to the synaptic connection between the motor neuron and skeletal muscle fiber at the neuromuscular junction also apply in the central nervous system. Synaptic transmission between central neurons is more complex, however, for several reasons. First, although most muscle fibers are innervated by only one motor neuron, a central nerve cell (such as the motor neuron in the spinal cord) receives connections from hundreds or even thousands of neurons” (p. 210). | Structure/organization of the nervous system | Kept  (used to develop statement 16) |
|  |  | 15 | Inputs are coordinated in the postsynaptic neuron by a process called *neuronal integration.* This cellular process reflects the task that confronts the nervous system as a whole: decision making. A cell at any given moment has two options: to fire or not to fire an action potential. Charles Sherrington described the brain's ability to choose between competing alternatives as the *integrative action of the nervous system.* He regarded this decision making as the brain' s most fundamental operation (p. 227). | Cell structure and function | Kept  (used to develop statement 8) |
|  |  |  |  | Cognition and other complex brain functions |  |
| 11 | [Modulation of Synaptic Transmission: Second Messengers](https://neurology.mhmedical.com/content.aspx?bookid=1049&sectionid=59138635) |  |  |  |  |
| 12 | [Transmitter Release](https://neurology.mhmedical.com/content.aspx?bookid=1049&sectionid=59138636) | 16 | Long-term changes in presynaptic and postsynaptic mechanisms are crucial to development and learning (p. 283). | Cell structure and function | Kept  (used to develop statement 14 and 15) |
|  |  |  |  | Plasticity |  |
| 13 | [Neurotransmitters](https://neurology.mhmedical.com/content.aspx?bookid=1049&sectionid=59138637) | 17 | The concept that nerve stimulation led to release of chemical signals was elaborated as early as 1905 … (p. 289). | Cell structure and function | Kept  (used to develop statement 10) |
|  |  | 18 | , … the concept of a transmitter … (p. 290). | Cell structure and function | Discarded: Considered too detailed. It is closely related to the previous concept. |
| 14 | [Diseases of the Nerve and Motor Unit](https://neurology.mhmedical.com/content.aspx?bookid=1049&sectionid=59138638) |  |  |  |  |
| 15 | [The Organization of the Central Nervous System](https://neurology.mhmedical.com/content.aspx?bookid=1049&sectionid=59138641) |  |  |  |  |
| 16 | [The Functional Organization of Perception and Movement](https://neurology.mhmedical.com/content.aspx?bookid=1049&sectionid=59138642) |  |  |  |  |
| 17 | [From Nerve Cells to Cognition: The Internal Representations of Space and Action](https://neurology.mhmedical.com/content.aspx?bookid=1049&sectionid=59138643) | 19 | This discussion illustrates a key principle that we will consider again in Chapter 19, that action has a key role in perception (p. 374). | Cognition and other complex brain functions | Discarded: Considered too advanced.  Involves complex processing in the CNS. |
|  |  |  |  | Sensory systems |  |
|  |  |  |  | Motor systems |  |
| 18 | [The Organization of Cognition](https://neurology.mhmedical.com/content.aspx?bookid=1049&sectionid=59138644) | 20 | In Luria’s scheme, sensory information flows into the central nervous system through a series of synaptic relays from primary to secondary to tertiary sensory areas, whereas motor commands flow from tertiary to secondary to primary motor areas. The tertiary areas at the peak of these sensory and motor hierarchies interact and are the seats of cognitive function. More than 45 years after publication of Luria’s book these general principles are still accepted (p. 393). | Sensory systems | Kept  (used to develop statements 9 and 3) |
|  |  |  |  | Motor systems |  |
|  |  |  |  | Cognition and other complex brain functions |  |
|  |  |  |  | Structure/organization of the nervous system |  |
|  |  | 21 | In addition to serial processing another principle of cortical organization is that the same information is processed differently in parallel pathways. In the visual system for example, two major parallel pathways terminate in different higher-order areas of cortex. The dorsal stream processes spatial information (position, motion, speed) and projects to parietal association cortex. The ventral stream processes information about form (color, shape, texture) and projects to temporal association cortex (p. 396). | Structure/organization of the nervous system | Kept  (used to develop statement 16) |
|  |  |  |  | Cognition and other complex brain functions |  |
|  |  | 22 | The two main principles of cortical organization are serial and parallel processing (p. 409). | Structure/organization of the nervous system | Kept  (used to develop statement 16) |
| 19 | [Cognitive Functions of the Premotor Systems](https://neurology.mhmedical.com/content.aspx?bookid=1049&sectionid=59138645) |  |  |  |  |
| 20 | [Functional Imaging of Cognition](https://neurology.mhmedical.com/content.aspx?bookid=1049&sectionid=59138646) |  |  |  |  |
| 21 | [Sensory Coding](https://neurology.mhmedical.com/content.aspx?bookid=1049&sectionid=59138649) |  |  |  |  |
| 22 | [The Somatosensory System: Receptors and Central Pathways](https://neurology.mhmedical.com/content.aspx?bookid=1049&sectionid=59138650) | 23 | The bodily senses mediate a wide range of experiences that are important for normal bodily function and for survival. Although diverse, they share common pathways and common principles of organization. The most important of those principles is specificity: Each of the bodily senses arises from a specific type of receptor distributed throughout the body. Mechanoreceptors are sensitive to specific aspects of local tissue distortion, thermoreceptors to particular temperature ranges and shifts in temperature, and chemoreceptors to particular molecular structures (p. 495) | Sensory systems | Kept  (used to develop statements 18, and 17) |
|  |  |  |  | Motor systems |  |
|  |  |  |  | Cognition and other complex brain functions |  |
|  |  |  |  | Structure/organization of the nervous system |  |
| 23 | [Touch](https://neurology.mhmedical.com/content.aspx?bookid=1049&sectionid=59138651) |  |  |  |  |
| 24 | [Pain](https://neurology.mhmedical.com/content.aspx?bookid=1049&sectionid=59138652) | 24 | The variability of the perception of pain is yet another example of a principle that we have encountered in earlier chapters: Pain is not the direct expression of a sensory event but rather the product of elaborate processing by the brain of a variety of neural signals (p. 530). | Sensory systems | Discarded: Considered too advanced.  Involves complex processing in the CNS. |
|  |  |  |  | Cognition and other complex brain functions |  |
|  |  | 25 | The concept that the convergence of sensory inputs onto spinal projection neurons regulates pain processing first emerged in the 1960s (p. 545). | Sensory systems | Discarded: Considered too advanced and specific on pain processing. |
|  |  |  |  | Structure/organization of the nervous system |  |
|  |  |  |  | Cognition and other complex brain functions |  |
|  |  | 26 | Nevertheless, the core concept of convergence of different sensory modalities has provided an important basis for the design of new pain therapies (p. 545). | Structure/organization of the nervous system | Kept  (used to develop statement 16) |
|  |  |  |  | Sensory systems |  |
| 25 | [The Constructive Nature of Visual Processing](https://neurology.mhmedical.com/content.aspx?bookid=1049&sectionid=59138653) | 27 | Because distributed processing is one of the main organizational principles in the neurobiology of vision, one must have a grasp of the anatomical pathways of the visual system to understand fully the physiological description of visual processing in later chapters (p. 557). | Structure/organization of the nervous system | Kept  (used to develop statement 21) |
|  |  |  |  | Sensory systems |  |
|  |  | 28 | The principle of good continuation is also seen in contour saliency (p. 558). | Sensory systems | Discarded: Considered too advanced.  Involves complex processes in the CNS. |
|  |  | 29 | In 1906 Charles Sherrington coined the term receptive field in his analysis of the scratch withdrawal reflex. The whole collection of points of skin surface from which the scratch reflex can be elicited is termed the receptive field of that reflex. When it became possible to record from single neurons in the eye, H. Keffler Hartline applied the concept of the receptive field his study of the retina of the horseshoe crab, *Limnulus.* "The region of the retina which must be illuminated in order to obtain a response in any given fiber … is termed the receptive field of that fiber." In the visual system a neuron's receptive field represents a small window on visual space (p. 564). | Sensory systems | Discarded: Considered too advanced.  Involves complex processes in the CNS. It is also of less relevance since it is specific for the senses and the senses is a topic separate from the nervous system in secondary school. |
|  |  |  |  | Structure/organization of the nervous system |  |
| 26 | [Low-Level Visual Processing: The Retina](https://neurology.mhmedical.com/content.aspx?bookid=1049&sectionid=59138654) |  |  |  |  |
| 27 | [Intermediate-Level Visual Processing and Visual Primitives](https://neurology.mhmedical.com/content.aspx?bookid=1049&sectionid=59138655) | 30 | Our ability to perceive an object' s size and color as constant illustrates again the fundamental principle of the visual system. It does not record images passively, like a camera, but instead uses transient and variable stimulation of the retina to construct representations of a stable three-dimensional world (p. 612). | Sensory systems | Discarded: Considered too advanced.  Involves complex processes in the CNS. |
|  |  |  |  | Cognition and other complex brain functions |  |
| 28 | [High-Level Visual Processing: Cognitive Influences](https://neurology.mhmedical.com/content.aspx?bookid=1049&sectionid=59138656) |  |  |  |  |
| 29 | [Visual Processing and Action](https://neurology.mhmedical.com/content.aspx?bookid=1049&sectionid=59138657) |  |  |  |  |
| 30 | [The Inner Ear](https://neurology.mhmedical.com/content.aspx?bookid=1049&sectionid=59138658) |  |  |  |  |
| 31 | [The Auditory Central Nervous System](https://neurology.mhmedical.com/content.aspx?bookid=1049&sectionid=59138659) | 31 | Nonetheless, although the details may differ between systems, the basic concept holds that sensory systems decompose stimuli into features and analyze these in discrete pathways (p. 705). | Sensory systems | Discarded: Considered too advanced.  Involves complex processes in the CNS. |
|  |  |  |  | Cognition and other complex brain functions |  |
| 32 | [Smell and Taste: The Chemical Senses](https://neurology.mhmedical.com/content.aspx?bookid=1049&sectionid=59138660) | 32 | The "one neuron, one receptor" principle observed in vertebrates and insects does not operate in nematodes as the number of neurons is much smaller than the number of receptors (p. 725). | Sensory systems | Discarded: Considered too specific. It only applies to the receptor cells of the olfactory and visual system. |
|  |  |  |  | Cell structure and function |  |
| 33 | [The Organization and Planning of Movement](https://neurology.mhmedical.com/content.aspx?bookid=1049&sectionid=59138663) |  |  |  |  |
| 34 | [The Motor Unit and Muscle Action](https://neurology.mhmedical.com/content.aspx?bookid=1049&sectionid=59138664) | 33 | The smallest motor neuron is recruited first and the largest motor neuron last (Figure 34-5). This effect is known as the size principle of motor neuron recruitment, a principle enunciated by Elwood Henneman in 1957 (p. 773). | Motor systems | Discarded: Considered too specific. |
|  |  |  |  | Structure/organization of the nervous system |  |
|  |  | 34 | A motor neuron and the muscle fibers it innervates are known as a motor unit the basic functional unit by which the nervous system controls movement, a concept proposed by Charles Sherrington in 1925 (p. 768). | Motor systems | Kept  (used to develop statement 3) |
|  |  |  |  | Structure/organization of the nervous system |  |
| 35 | [Spinal Reflexes](https://neurology.mhmedical.com/content.aspx?bookid=1049&sectionid=59138665) | 35 | Three important principles are illustrated by these examples. First, neural signaling in reflex pathways is adjusted according to the motor task. The state of the reflex pathways for any task is referred to as the *functional set.* Exactly how a functional set is established for most motor tasks is largely unknown, and unraveling the underlying mechanisms is one of the challenging areas of contemporary research on motor systems. Second, sensory input from a localized source generally produces coordinated reflex responses in several muscles at once, some of which may be distant from the stimulus. Third, supraspinal centers play an important role in modulating and adapting spinal reflexes, even to the extent of reversing movements when appropriate (p. 792). | Motor systems | Discarded: Considered too advanced. Involves complex processes in the CNS. |
|  |  |  |  | Sensory systems |  |
|  |  |  |  | Structure/organization of the nervous system |  |
|  |  |  |  | Cognition and other complex brain functions |  |
| 36 | [Locomotion](https://neurology.mhmedical.com/content.aspx?bookid=1049&sectionid=59138666) | 36 | He therefore proposed the concept of the half-center, whereby flexors and extensors inhibit each other reciprocally, giving rise to alternating stepping movements. Four conclusions can be drawn from these early studies (p. 813). | Motor systems  Structure/organization of the nervous system  Sensory systems | Discarded: Considered too advanced.  Involves complex processes in the CNS. |
|  |  | 37 | In light of these findings there is reason to believe that human walking relies on the same general principles of neuronal organization as quadrupedal walking: intrinsic oscillatory networks are activated and modulated by other brain structures and by afferent input (p. 830). | Motor systems | Discarded: Considered to advanced. Involves complex processes in the CNS. |
|  |  |  |  | Structure/organization of the nervous system |  |
|  |  |  |  | Sensory systems |  |
| 37 | [Voluntary Movement: The Primary Motor Cortex](https://neurology.mhmedical.com/content.aspx?bookid=1049&sectionid=59138667) |  |  |  |  |
| 38 | [Voluntary Movement: The Parietal and Premotor Cortex](https://neurology.mhmedical.com/content.aspx?bookid=1049&sectionid=59138668) | 38 | To help understand how visual information about an object is transformed into specific movements to grasp and manipulate it, we shall speak of the *affordances* of an object, a concept introduced by James Gibson. When we observe an object our visual system automatically identifies the parts of it that allow for efficient manipulation of it (p. 877). | Sensory systems | Discarded: Considered too advanced.  Involves complex processes in the CNS. |
|  |  |  |  | Motor systems |  |
|  |  |  |  | Cognition and other complex brain functions |  |
| 39 | [The Control of Gaze](https://neurology.mhmedical.com/content.aspx?bookid=1049&sectionid=59138669) |  |  |  |  |
| 40 | [The Vestibular System](https://neurology.mhmedical.com/content.aspx?bookid=1049&sectionid=59138670) |  |  |  |  |
| 41 | [Posture](https://neurology.mhmedical.com/content.aspx?bookid=1049&sectionid=59138671) | 39 | Henry Head, a neurologist working in the early part of the 20th century, described the body schema as a dynamic system in which both spatial and temporal features are continually updated, a concept that remains current (p. 951). | Sensory systems | Discarded: Considered too advanced.  Involves complex processes in the CNS. |
|  |  |  |  | Cognition and other complex brain functions |  |
|  |  |  |  | Plasticity |  |
| 42 | [The Cerebellum](https://neurology.mhmedical.com/content.aspx?bookid=1049&sectionid=59138672) | 40 | One fundamental principle of cerebellar operation can be appreciated on the basis of two important pathways from the spinal interneurons. The ventral and dorsal spinocerebellar tracts both transmit signals from the spinal cord directly to the cerebellar cortex but convey two different kinds of information. The *dorsal spinocerebellar tract* conveys somatosensory information from muscle and joint receptors, providing the cerebellum with sensory feedback about the consequences of the movement. This information flows whether the limbs are moved passively or voluntarily. In contrast, the *ventral spinocerebellar tract* is active only during active movements. Its cells of origin receive the same inputs as spinal motor neurons and interneurons, and it transmits an efference copy or corollary discharge of spinal motor neuron activity that informs the cerebellum about the movement commands assembled at the spinal cord. The cerebellum is thought to compare this information on planned movement with the actual movement reported by the dorsal spinocerebellar tract in order to determine whether the motor command must be modified to achieve the desired movement (p. 969 – 970). | Sensory systems | Discarded: Considered too advanced. Involves complex processes in the CNS. |
|  |  |  |  | Motor systems |  |
|  |  |  |  | Structure/organization of the nervous system |  |
|  |  |  |  | Cognition and other complex brain functions |  |
|  |  | 41 | Spinocerebellar Regulation of Movement Follows Three Organizational Principles … First, both Purkinje neurons and deep cerebellar nucleus neurons discharge vigorously in relation to voluntary movements. … Second, the cerebellum provides feed-forward control of muscle contractions to regulate the timing of movements. Third, the cerebellum has internal models of the limbs that automatically take account of limb structure (p. 972 - 973). | Structure/organization of the nervous system | Discarded: Considered too advanced. Involves complex processes in the CNS. |
|  |  |  |  | Cell structure and function |  |
|  |  |  |  | Motor systems |  |
|  |  |  |  | Sensory systems |  |
| 43 | [The Basal Ganglia](https://neurology.mhmedical.com/content.aspx?bookid=1049&sectionid=59138673) | 42 | The larger motor circuit consists of segregated subcircuits, each centered on an individual precentral motor field. These subcircuits are believed to be responsible for different aspects of motor processing, such as motor planning, coordination of sequences of movement, or movement execution. Evidence for the subcircuit organization comes from anatomical studies … Segregated anterograde transsynaptic transport of input from cortical areas to the striatum and pallidum has likewise been shown, providing further support for the segregated circuit concept (p. 986). | Motor systems | Discarded: Considered too advanced.  Involves complex processes in the CNS. |
|  |  |  |  | Structure/organization of the nervous system |  |
|  |  |  |  | Cognition and other complex brain functions |  |
|  |  | 43 | The concept that the basal ganglia play a role in action selection, in the broadest sense, implies that they also participate in the acquisition of behaviors that lead to a reward or reinforcement and the avoidance of acts that lead to punishment or adverse outcomes (p. 986). | Motor systems | Discarded: Considered too advanced.  Involves complex processes in the CNS. |
|  |  |  |  | Cognition and other complex brain functions |  |
|  |  |  |  | Sensory systems |  |
| 44 | [Genetic Mechanisms in Degenerative Diseases of the Nervous System](https://neurology.mhmedical.com/content.aspx?bookid=1049&sectionid=59138674) |  |  |  |  |
| 45 | [The Sensory, Motor, and Reflex Functions of the Brain Stem](https://neurology.mhmedical.com/content.aspx?bookid=1049&sectionid=59138677) |  |  |  |  |
| 46 | [The Modulatory Functions of the Brain Stem](https://neurology.mhmedical.com/content.aspx?bookid=1049&sectionid=59138678) | 44 | Two neurological principles are important for determining the cause of coma. First, any decrease in the level of consciousness (decreased arousal) implies dysfunction of either both cerebral hemispheres or of the ascending arousal system (or its projections in the thalamus or hypothalamus). Second, one can pinpoint the levels of the brain stem that are damaged by determining abnormalities of reflexes mediated by cranial nerves, which often accompany coma. (p. 1051). | Cognition and other complex brain functions | Kept  (used to develop statement 20) |
|  |  |  |  | Structure/organization of the nervous system |  |
| 47 | [The Autonomic Motor System and the Hypothalamus](https://neurology.mhmedical.com/content.aspx?bookid=1049&sectionid=59138679) | 45 | Building on this idea, in the 1930s Walter B. Cannon introduced the concept of homeostasis to describe the mechanisms that maintain within a narrow physiological range the constancy of composition of the bodily fluids, body temperature, blood pressure, and other physiological variables. ... All homeostatic behavior, including control of circulation, arises from neural modulation of the physiological properties of organ systems, mediated by hypothalamic control of the autonomic motor system and the endocrine system (p. 1057). | Structure/organization of the nervous system | Kept  (used to develop statement 23) |
|  |  |  |  | Sensory systems |  |
|  |  |  |  | Motor systems |  |
|  |  | 46 | In addition to acting on different receptors in different postsynaptic cells, one transmitter can activate two or more receptor types in the same postsynaptic cell. This principle was first discovered in sympathetic ganglia where ACh activates both nicotinic and muscarinic postsynaptic receptors to produce both a fast and slow excitatory postsynaptic potential (EPSP) (p. 1061). | Cell structure and function | Discarded: Considered too advanced/specific on the molecular level. |
|  |  | 47 | The cellular principles of co-transmission have been elucidated in part in a series of studies of paravertebral sympathetic ganglia in the bullfrog (p. 1063). | Cell structure and function | Discarded: Considered too advanced/specific on the molecular level. |
|  |  | 48 | This discovery illustrated three general principles, … First, peptides can mediate very slow synaptic events. Second, peptides act diffusely at a distance and thereby provide cross talk between different sympathetic cell types. Finally, different transmitters can share intracellular signaling pathways (p. 1066). | Cell structure and function | Discarded: These principles are an extension of the principle above. They are therefore considered too advanced on the molecular level. |
| 48 | [Emotions and Feelings](https://neurology.mhmedical.com/content.aspx?bookid=1049&sectionid=59138680) |  |  |  |  |
| 49 | [Homeostasis, Motivation, and Addictive States](https://neurology.mhmedical.com/content.aspx?bookid=1049&sectionid=59138681) |  |  |  |  |
| 50 | [Seizures and Epilepsy](https://neurology.mhmedical.com/content.aspx?bookid=1049&sectionid=59138682) |  |  |  |  |
| 51 | [Sleep and Dreaming](https://neurology.mhmedical.com/content.aspx?bookid=1049&sectionid=59138683) |  |  |  |  |
| 52 | [Patterning the Nervous System](https://neurology.mhmedical.com/content.aspx?bookid=1049&sectionid=59138686) | 49 | Studies of the logic of ventral neuronal patterning have thus shown that the fate of a neuron is determined in part by the actions of transcriptional repressors rather than activators. This principle operates in many other tissues and organisms, … (p. 1176). | Plasticity | Discarded: Considered too advanced on the genetic level. |
| 53 | [Differentiation and Survival of Nerve Cells](https://neurology.mhmedical.com/content.aspx?bookid=1049&sectionid=59138687) |  |  |  |  |
| 54 | [The Growth and Guidance of Axons](https://neurology.mhmedical.com/content.aspx?bookid=1049&sectionid=59138688) |  |  |  |  |
| 55 | [Formation and Elimination of Synapses](https://neurology.mhmedical.com/content.aspx?bookid=1049&sectionid=59138689) | 50 | Synapses in the central nervous system are structurally similar to neuromuscular junctions, and they function in a similar way. Their formation also adheres to the principles of development of the neuromuscular junction: Pre- and postsynaptic elements regulate each other's differentiation by organizing presynthesized synaptic components rather than by inducing expression of specific genes, and synapses develop in a progressive series of steps (p. 1249 – 1250). | Plasticity | Discarded: Considered too advanced on the molecular and genetic level |
| 56 | [Experience and the Refinement of Synaptic Connections](https://neurology.mhmedical.com/content.aspx?bookid=1049&sectionid=59138690) | 51 | Competition and cooperation are not simply the outcome of neural activity per se, or of differences in absolute levels of activity among axons. Instead, they appear to depend on precise temporal patterns of activity in the competing (or cooperating) axons. The principle was dramatically illustrated by Hubel and Wiesel in a set of studies that examined stereoscopic vision-the perception of depth (p. 1265 - 1266). | Plasticity | Discarded: Considered too advanced. |
| 57 | [Repairing the Damaged Brain](https://neurology.mhmedical.com/content.aspx?bookid=1049&sectionid=59138691) | 52 | The principle that embryonic neurons and glia arise from multipotential progenitors also applies to neurons born in adults (p. 1297). | Cell structure and function | Kept  (used to develop statement 24) |
|  |  |  |  | Plasticity |  |
| 58 | [Sexual Differentiation of the Nervous System](https://neurology.mhmedical.com/content.aspx?bookid=1049&sectionid=59138692) |  |  |  |  |
| 59 | [The Aging Brain](https://neurology.mhmedical.com/content.aspx?bookid=1049&sectionid=59138693) |  |  |  |  |
| 60 | [Language](https://neurology.mhmedical.com/content.aspx?bookid=1049&sectionid=59138696) |  |  |  |  |
| 61 | [Disorders of Conscious and Unconscious Mental Processes](https://neurology.mhmedical.com/content.aspx?bookid=1049&sectionid=59138697) | 53 | The first form of alternative psychological therapy to emerge from laboratory studies is known as *behavior therapy.* The fundamental assumption of this approach is that maladaptive behavior is learned and can therefore be eliminated by applying the Pavlovian and Skinnerian principles of stimulus-response learning (p. 1374). | Sensory systems | Discarded: Of little relevance because it involves several principles that are specific to one type of learning procedure. |
|  |  |  |  | Motor systems |  |
|  |  |  |  | Plasticity |  |
|  |  |  |  | Cognition and other complex brain functions |  |
| 62 | [Disorders of Thought and Volition: Schizophrenia](https://neurology.mhmedical.com/content.aspx?bookid=1049&sectionid=59138698) |  |  |  |  |
| 63 | [Disorders of Mood and Anxiety](https://neurology.mhmedical.com/content.aspx?bookid=1049&sectionid=59138699) |  |  |  |  |
| 64 | [Autism and Other Neurodevelopmental Disorders Affecting Cognition](https://neurology.mhmedical.com/content.aspx?bookid=1049&sectionid=59138700) |  |  |  |  |
| 65 | [Learning and Memory](https://neurology.mhmedical.com/content.aspx?bookid=1049&sectionid=59138701) | 54 | We began this chapter by noting three key principles:  (1) several different forms of learning and memory can be distinguished behaviorally, (2) memory can be analyzed in terms of discrete operations (encoding, storage, consolidation, and retrieval), and (3) imperfections and errors in remembering can provide telltale dues about learning and memory (p. 1458). | Plasticity | Discarded: Considered too advanced. |
|  |  |  |  | Motor systems |  |
|  |  |  |  | Cognition and other complex brain functions |  |
|  |  | 55 | "When an axon of cell A ... excites cell B and repeatedly or persistently takes part in firing it, some growth process or metabolic change takes place in one or both cells so that A's efficiency as one of the cells firing B is increased." A similar principle is involved in fine-tuning synaptic connections during the late stages of development (p. 1498). | Plasticity | Kept  (used to develop statements 14 and 15) |
|  |  |  |  | Cell structure and function |  |
| 66 | [Cellular Mechanisms of Implicit Memory Storage and the Biological Basis of Individuality](https://neurology.mhmedical.com/content.aspx?bookid=1049&sectionid=59138702) | 56 | A striking feature of implicit or procedural memory storage is that the recall of this memory is accomplished without recourse to conscious thought. Many aspects of personality, much of what we do in our daily life, is guided by implicit memory. These principles are consistent with a central tenet of psychoanalytic theory, the idea that we are unaware of much of our mental life. A great deal of what we experience-what we perceive, think, fantasize-cannot be directly accessed by conscious thought. Nor can we explain what often motivates our actions (p. 1483). | Plasticity | Discarded: Considered too advanced. Involves complex processes in the CNS. |
|  |  |  |  | Cognition and other complex brain functions |  |
| 67 | [Prefrontal Cortex, Hippocampus, and the Biology of Explicit Memory Storage](https://neurology.mhmedical.com/content.aspx?bookid=1049&sectionid=59138703) |  |  |  |  |
| App E |  | 57 | Starting from the 1940s researchers have proposed and studied many brain models in which sophisticated computations are performed by networks of simple neuron-like elements. Most models are based on two shared principles. First, our immediate experience is rooted in ongoing patterns of action potentials in brain cells. Second, our ability to learn from and remember past experiences is based at least partially on long-lasting modifications of synaptic connections. Although these principles are widely accepted by neuroscientists, they immediately suggest many difficult questions (p. 1581). | Cognition and other complex brain functions | Kept  (used to develop statements 6 and 15). |
|  |  |  |  | Cell structure and function |  |
|  |  |  |  | Structure/organization of the nervous system |  |
|  |  |  |  | Plasticity |  |
|  |  | 58 | As we shall see, this hierarchical conception of visual recognition of objects has been formulated precisely in a number of neural network models (p. 1589). | Structure/organization of the nervous system | Discarded: Considered too advanced.  Involves complex processes in the CNS. |
|  |  |  |  | Sensory systems |  |
|  |  |  |  | Cognition and other complex brain functions |  |
|  |  | 59 | To summarize, the cell assembly concept has been used to explain both long-term and short-term memory. According to this concept a long-term memory is stored as strengthened connections between neurons in a cell assembly, while a short-term memory is maintained by persistent activity of the neurons in a cell assembly (p. 1595). | Plasticity | Kept  (used to develop statement 15) |
|  |  |  |  | Cell structure and function |  |
|  |  |  |  | Structure/organization of the nervous system |  |
