# Supplementary material for: Identifying knowledge important to teach about the nervous system in the context of secondary biology and science education–A Delphi study
Source: PLoS One. 2021 Dec 21;16(12):e0260752. doi: 10.1371/journal.pone.0260752 (PMC8691623; doi:10.1371/journal.pone.0260752)
Supplement: S4 Table — The table shows the final list of premade statements (column 2), the major neuroscientific topic categories they were ascribed to (column 3), and the textbook citations (column 4) with citation number (column 3) which were used to develop them. (DOCX) [file pone.0260752.s004.docx]

**S4 Table. The development of premade statements.** The table shows the final list of premade statements (column 2), the major neuroscientific topic categories they were ascribed to (column 3), and the textbook citations (column 4) with citation number (column 3) which were used to develop them.

| **Nr** | **Statements** | **Topic Categories** | **Citation Nr** | **Citations from the textbook “Principles of neural science” used to develop statements** |
| --- | --- | --- | --- | --- |
| 1 | Neurons are the elementary building blocks and signaling elements of the nervous system | Structure/organization of the nervous system | 2 | Using Golgi's technique, Ramón y Cajal discovered that nervous tissue is not a syncytium, a continuous web of elements, but a network of discrete cells. In the course of this work Ramón y Cajal developed some of the key concepts and much of the early evidence for the *neuron doctrine –* the principle that individual neurons are the elementary building blocks and signaling elements of the nervous system (p. 6). |
| 2 | Neurons consist of three main structures: a cell body which is the metabolic center of the cell, dendrites which receive signals from other cells, and an axon with branches that sends signals to other cells | Cell structure and function | 6 | The principle that each neuron is a discrete cell with distinctive processes arising from its cell body and that neurons are the signaling units of the nervous system (p. 23).  Relevant details added from the literature:  “The cell body usually gives rise to two kinds of processes: several short dendrites and one long, tubular axon” (p. 22). “Dendrites branch out in tree-like fashion and are the main apparatus for receiving incoming signals from other cells. The axon typically extends some distance from the cell body and carries signals to other neurons”. (p. 22). |
| 3 | The nervous system can be organized into three types of neurons: a) Sensory neurons which bring information to the nervous system, b) Motor neurons which send information to muscles and glands, and c) Interneurons which send information between neurons | Structure/organization of the nervous system  Sensory systems  Motor systems | 23 | The bodily senses mediate a wide range of experiences that are important for normal bodily function and for survival. Although diverse, they share common pathways and common principles of organization. The most important of those principles is specificity: Each of the bodily senses arises from a specific type of receptor distributed throughout the body. Mechanoreceptors are sensitive to specific aspects of local tissue distortion, thermoreceptors to particular temperature ranges and shifts in temperature, and chemoreceptors to particular molecular structures (p. 495) |
|  |  |  | 20 | In Luria’s scheme, sensory information flows into the central nervous system through a series of synaptic relays from primary to secondary to tertiary sensory areas, whereas motor commands flow from tertiary to secondary to primary motor areas. The tertiary areas at the peak of these sensory and motor hierarchies interact and are the seats of cognitive function. More than 45 years after publication of Luria’s book these general principles are still accepted (p. 393). |
|  |  |  | 34 | A motor neuron and the muscle fibers it innervates are known as a motor unit the basic functional unit by which the nervous system controls movement, a concept proposed by Charles Sherrington in 1925 (p. 768).  Relevant details added from the literature:  “Nerve cells are also classified into three major functional categories: sensory neurons, motor neurons, and interneurons. Sensory neurons carry information from the body’s peripheral sensors into the nervous system …”. (p. 24).  “Motor neurons carry commands from the brain or spinal cord to muscles and glands …”. (p. 24).  “Interneurons are the most numerous …” (p. 24). |
| 4 | The nerve signal is an electrochemical signal that carries information | Cell structure and function | The first of the two additional statements | “To understand how this balance point is determined, bear in mind that the magnitude of the flux of an ion across a cell membrane is the product of its *electrochemical driving force* (the sum of the electrical and chemical driving forces) and the conductance of the membrane to the ion” (p. 131).  Relevant details added from the literature:  «Information is carried within neurons and from neurons to their target cells by electrical and chemical signals» (Kandel et al. 2013, p. 126). |
| 5 | The nerve signal consists of three main types of signals: 1) graded electrochemical signals which mainly occur in the dendrites, 2) all-or-none electrochemical signals (action potentials) which mainly occur in the axon, and 3) graded chemical signals which occurs between neurons | Cell structure and function | 9 | “… , almost all neurons can be described by a model neuron that has four functional components that generate the four types of signals: a receptive component, a summing or integrative component, a long-range signaling component, and a secretory component (Figure 2-9). This model neuron is the physiological expression of Ramón y Cajal’s principle of dynamic polarization” (p. 29 -30).  Relevant details added from the literature:  “Thus, unlike the action potential, which is all-or-none, receptor potentials are graded”. (p. 31).  “Like the receptor potential, the synaptic potential is graded”. (p. 31).  “When an action potential reaches a neuron’s terminal it stimulates the release of chemical substances from the cell. These substances, called neurotransmitters, … Once released, the neurotransmitter is the neuron’s output signal. Like the input signal, it is graded.” (p. 35). |
| 6 | Action potentials are the signals by which the brain receives, analyzes, interprets, and conveys information | Cell structure and function  Cognition and other complex brain functions | 57 | Starting from the 1940s researchers have proposed and studied many brain models in which sophisticated computations are performed by networks of simple neuron-like elements. Most models are based on two shared principles. First, our immediate experience is rooted in ongoing patterns of action potentials in brain cells (p. 1581). |
|  |  |  | 5 | Here we see a key principle of brain function: the information conveyed by an action potential is determined not by the form of the signal but by the pathway the signal travels in the brain. The brain analyzes and interprets patterns of incoming electrical signals and their pathways, and in turn creates our sensations of sight, touch, smell, and sound (p. 23). |
| 7 | Inside neurons, the nerve signal flows in one direction only: from the dendrites to the axon terminals | Cell structure and function | 7 | The first of these has come to be known as the *principle of dynamic polarization.* It states that electrical signals within a nerve cell flow only in one direction: from the receiving sites of the neuron, usually the dendrites and cell body, to the trigger region at the axon (p. 24). |
| 8 | A neuron sends a nerve signal to other neurons only if the sum of its received signals is above a certain threshold | Cell structure and function | 15 | “Inputs are coordinated in the postsynaptic neuron by a process called *neuronal integration.* This cellular process reflects the task that confronts the nervous system as a whole: decision making. A cell at any given moment has two options: to fire or not to fire an action potential. Charles Sherrington described the brain's ability to choose between competing alternatives as the *integrative action of the nervous system.* He regarded this decision making as the brain' s most fundamental operation (p. 227).  Relevant details added from the literature:  “The action potential is all-or-none: Stimuli below the threshold do not produce a signal, but stimuli above the threshold all produce the signals of the same amplitude”. (p. 33).  “The action potential is conducted down the cell’s axon to the axon’s terminal, where it initiates an elaborate chemical communication with other neurons or muscle cells”. (p. 31). |
| 9 | Nerve signals pass from one neuron to the next at specialized zones called synapses | Cell structure and function | 20 | “In Luria’s scheme, sensory information flows into the central nervous system through a series of synaptic relays from primary to secondary to tertiary sensory areas, whereas motor commands flow from tertiary to secondary to primary motor areas. The tertiary areas at the peak of these sensory and motor hierarchies interact and are the seats of cognitive function. More than 45 years after publication of Luria’s book these general principles are still accepted” (p. 393).  Relevant details added from the literature:  “Near its end the axon divides into fine branches that contact other neurons at specialized zones of communication known as synapses”. (p. 23). |
| 10 | At synapses, the nerve signal is transmitted from one cell to the next either by diffusion of molecules over a tiny gap (chemical synapses) or by electrical current (electrical synapses) | Cell structure and function | 10 | “, … all neurons make use of one of the two basic forms of synaptic transmission: electrical or chemical (p. 177).  Relevant details added from the literature:  “Based on the structure of the apposition, synapses are categorized into two major groups: electrical and chemical”. (p. 175). |
|  |  |  | 17 | “The concept that nerve stimulation led to release of chemical signals was elaborated as early as 1905 …”. (p. 289). |
| 11 | At chemical synapses, the nerve signal is transmitted in one direction only: from the axon terminals of the presynaptic cell to the dendrites of the postsynaptic cell | Cell structure and function  Structure/organization of the nervous system | 9 | “Regardless of cell size and shape, transmitter biochemistry, or behavioral function, almost all neurons can be described by a model neuron that has four functional components that generate the four types of signals: a receptive component, a summing or integrative component, a long-range signaling component, and a secretory component (Figure 2-9). This model neuron is the physiological expression of Ramón y Cajal's principle of dynamic polarization (p. 30). |
|  |  |  | 8 | “The other principle advanced by Ramón y Cajal is that of *connectional specificity,* which states that nerve cells do not connect randomly with one another in the formation of networks. Rather each cell makes specific connections at particular contact points with certain postsynaptic target cells but not with others” (p. 24).  Relevant details added from the literature:  ”The presynaptic cell transmits signals from specialized enlarged regions of its axon’s branches, called presynaptic terminals … Most presynaptic terminals end on the postsynaptic neuron’s dendrites; …” (p. 23). |
| 12 | There are two main functional types of chemical synapses: Excitatory synapses which increase the probability of the postsynaptic neuron to send an action potential, and 2) Inhibitory synapses which decrease the probability of the postsynaptic neuron to send an action potential | Cell structure and function | 11 | “Because chemical synaptic transmission is so central to understanding brain and behavior, it is examined in detail in the next four chapters”… “… chemical synapses are capable of more variable signaling and thus can produce more complex behaviors. They can mediate either excitatory or inhibitory actions in postsynaptic cells and produce electrical changes in the postsynaptic cell that last from milliseconds to many minutes” (p. 177).  Relevant details added from the literature:  “Although glutamatergic excitatory synapses account for the vast majority of synapses in the brain, inhibitory synapses play an essential role in the nervous system both by preventing too much excitation and by helping coordinate activity among networks of neurons” (p. 222). |
| 13 | A neuron receives both excitatory and inhibitory nerve signals from other cells, but can only send one type of signal, excitatory or inhibitory, to all its postsynaptic cells, not both | Structure/organization of the nervous system  Cell structure and function | 12 | “Thus, many principles that apply to the synaptic connection between the motor neuron and skeletal muscle fiber at the neuromuscular junction also apply in the central nervous system. Synaptic transmission between central neurons is more complex, however, for several reasons. First, … Second, muscle fibers receive only excitatory inputs, whereas central neurons receive both excitatory and inhibitory inputs” (p. 210).  Relevant details added from the literature:  “The synaptic terminals of excitatory and inhibitory neurons can be distinguished by their morphology”. (p. 213). |
|  |  |  | 13 | “Excitatory and Inhibitory Synaptic Actions Are Integrated by the Cell into a Single Output” (p. 210). |
| 14 | Synapses are formed by genetic programs during embryonic development but are modified through interactions with the internal and external environment | Cell structure and function  Plasticity | 16 | “Long-term changes in presynaptic and postsynaptic mechanisms are crucial to development and learning” (p. 283).  Relevant details added from the literature:  “Throughout this chapter we emphasize the interplay of molecular programs and neural activity in shaping synaptic patterns … The initial steps in this process appear to be “hardwired” by molecular programs” (p. 1234).  “The current view is that molecular matching predominates during embryonic development, and that activity and experience modify circuits after they have formed”. (p. 1218). |
|  |  |  | 55 | "When an axon of cell A ... excites cell B and repeatedly or persistently takes part in firing it, same growth process or metabolic change takes place in one or both cells so that A's efficiency as one of the cells firing B is increased." A similar principle is involved in fine-tuning synaptic connections during the late stages of development (p. 1498). |
|  |  |  | 1 | “One of the chief ideas we shall develop in this book is that the specificity of the synaptic connections established during development underlie perceptions, action, emotion, and learning”. (p. 4). |
| 15 | The effectiveness of synapses changes as we learn new things, and these changes are necessary to form memories | Cell structure and function  Plasticity  Cognition and other complex brain functions | 16 | “Long-term changes in presynaptic and postsynaptic mechanisms are crucial to development and learning” (p. 283). |
|  |  |  | 55 | "When an axon of cell A ... excites cell B and repeatedly or persistently takes part in firing it, some growth process or metabolic change takes place in one or both cells so that A's efficiency as one of the cells firing B is increased." A similar principle is involved in fine-tuning synaptic connections during the late stages of development (p. 1498).  Relevant details added from the literature:  “Moreover, the strength of both forms of synaptic transmission can be enhanced or diminished by cellular activity. This plasticity is crucial to memory …”. (p. 177).  «Chemical synapses are functionally and anatomically modified through experience and learning» (Kandel et al. 2013, p. 37). |
|  |  |  | 57 | Starting from the 1940s researchers have proposed and studied many brain models in which sophisticated computations are performed by networks of simple neuron-like elements. Most models are based on two shared principles. First, our immediate experience is rooted in ongoing patterns of action potentials in brain cells. Second, our ability to learn from and remember past experiences is based at least partially on long-lasting modifications of synaptic connections. Although these principles are widely accepted by neuroscientists, they immediately suggest many difficult questions (p. 1581). |
|  |  |  | 59 | To summarize, the cell assembly concept has been used to explain both long-term and short-term memory. According to this concept a long-term memory is stored as strengthened connections between neurons in a cell assembly, while a short-term memory is maintained by persistent activity of the neurons in a cell assembly (p. 1595). |
| 16 | Each neuron in the central nervous system receives information from many other neurons and sends information to many other neurons to form networks and share information | Structure/organization of the nervous system  Cell structure and function | 14 | “Thus, many principles that apply to the synaptic connection between the motor neuron and skeletal muscle fiber at the neuromuscular junction also apply in the central nervous system. Synaptic transmission between central neurons is more complex, however, for several reasons. First, although most muscle fibers are innervated by only one motor neuron, a central nerve cell (such as the motor neuron in the spinal cord) receives connections from hundreds or even thousands of neurons” (p. 210).  Relevant details added from the literature:  “Thus it is not accurate to think of a mental process as being mediated by a chain of nerve cells connected in series – one cell connected directly to the next – for in such an arrangement the entire process breaks down when a single connection is disrupted”. (p. 17).  “Near its end the axon divides into fine branches that contact other neurons at specialized zones of communication known as synapses”. (p. 23).  “An average neuron forms and receives 1,000 to 10,000 synaptic connections”. (p. 175). |
|  |  |  | 21 | In addition to serial processing another principle of cortical organization is that the same information is processed differently in parallel pathways. In the visual system for example, two major parallel pathways terminate in different higher-order areas of cortex. The dorsal stream processes spatial information (position, motion, speed) and projects to parietal association cortex. The ventral stream processes information about form (color, shape, texture) and projects to temporal association cortex (p. 396). |
|  |  |  | 22 | The two main principles of cortical organization are serial and parallel processing (p. 409). |
|  |  |  | 26 | Nevertheless, the core concept of convergence of different sensory modalities has provided an important basis for the design of new pain therapies (p. 545). |
| 17 | Each neuron makes specific connections with certain postsynaptic target cells but not with others | Structure/organization of the nervous system  Cell structure and function | 8 | The other principle advanced by Ramón y Cajal is that of *connectional specificity,* which states that nerve cells do not connect randomly with one another in the formation of networks. Rather each cell makes specific connections at particular contact points with certain postsynaptic target cells but not with others (p. 24). |
|  |  |  | 23 | The bodily senses mediate a wide range of experiences that are important for normal bodily function and for survival. Although diverse, they share common pathways and common principles of organization. The most important of those principles is specificity: Each of the bodily senses arises from a specific type of receptor distributed throughout the body. Mechanoreceptors are sensitive to specific aspects of local tissue distortion, thermoreceptors to particular temperature ranges and shifts in temperature, and chemoreceptors to particular molecular structures (p. 495) |
| 18 | Our perceptions, thoughts, feelings and behavior are mainly determined by which pathways the nerve signal takes through the network of neurons | Cognition and other complex brain functions  Structure/organization of the nervous system  Cell structure and function  Sensory systems  Motor system | 5 | Here we see a key principle of brain function: the information conveyed by an action potential is determined not by the form of the signal but by the pathway the signal travels in the brain (p. 23). |
|  |  |  | 1 | “One of the chief ideas we shall develop in this book is that the specificity of the synaptic connections established during development underlie perceptions, action, emotion, and learning”. (p. 4). |
|  |  |  | 23 | The bodily senses mediate a wide range of experiences that are important for normal bodily function and for survival. Although diverse, they share common pathways and common principles of organization. The most important of those principles is specificity: Each of the bodily senses arises from a specific type of receptor distributed throughout the body. Mechanoreceptors are sensitive to specific aspects of local tissue distortion, thermoreceptors to particular temperature ranges and shifts in temperature, and chemoreceptors to particular molecular structures (p. 495) |
| 19 | The intensity of our perceptions and actions are mainly determined by the frequency of action potentials elicited by the sensory and motor neurons, respectively. High frequency gives rise to high intensity and low frequency to low intensity | Sensory systems  Motor system  Cognition and other complex brain functions  Cell structure and function | 5 | Here we see a key principle of brain function: the information conveyed by an action potential is determined not by the form of the signal but by the pathway the signal travels in the brain. The brain analyzes and interprets patterns of incoming electrical signals and their pathways, and in turn creates our sensations of sight, touch, smell, and sound (p. 23).  Relevant details added from the literature:  “As Adrian put it in 1928, summarizing his work on sensory fibers: “all impulses are very much alike, whether the message is destined to arouse the sensation of light, of touch, or of pain; if they are crowded together the sensation is intense, if they are separated by long intervals the sensation is correspondingly feeble”. Thus, what determines the intensity of sensation or speed of movement is the frequency of the action potentials” (p. 33). |
| 20 | The brain has a continuous self-sustaining activity. Sensory input cannot stop or start this activity, only modify it | Cell structure and function  Structure/organization of the nervous system  Sensory systems | 44 | “Two neurological principles are important for determining the cause of coma. First, any decrease in the level of consciousness (decreased arousal) implies dysfunction of either both cerebral hemispheres or of the ascending arousal system (or its projections in the thalamus or hypothalamus). Second, one can pinpoint the levels of the brain stem that are damaged by determining abnormalities of reflexes mediated by cranial nerves, which often accompany coma” (p. 1051).  Relevant details added from the literature:  «Neurons that are spontaneously active do not require sensory or synaptic input to fire action potentials …» (Kandel et al. 2013, p. 35). |
| 21 | The brain has distinct regions that are specialized for different functions, like perception, movement, language, thoughts, emotions, etc. However, different brain regions are interlinked, and proper brain function requires coordinated action of neurons in many brain regions | Structure/organization of the nervous system  Sensory systems  Motor systems  Cognition and other complex brain functions | 4 | As we shall see in later chapters, functional specialization is a key organizing principle in the cerebral cortex, extending even to individual columns of cells within a functional area (p. 13). |
|  |  |  | 3 | In placing the principle of localized function within a connectionist framework, Wernicke realized that different components of a single behavior are likely to be processed in several regions of the brain. He was thus the first to advance the idea of *distributed processing,* now a central tenet of neural science (p. 12). |
|  |  |  | 27 | Because distributed processing is one of the main organizational principles in the neurobiology of vision, one must have a grasp of the anatomical pathways of the visual system to understand fully the physiological description of visual processing in later chapters (p. 557). |
| 22 | Brain structure and function is maintained by regularly challenging the brain with physical and mental activity – “use it or lose it” | Structure/organization of the nervous system  Cognition and other complex brain functions  Plasticity | The second of the two additional statements | “Continuously challenging the brain with physical and mental activity helps maintain its structure and function — “use it or lose it”” Brainfacts (2020).  Relevant details added from the literature:  “Human amblyopia can be ameliorated in adulthood by training” (p. 1281).  “After training there is a substantial enlargement of the cortical representation of the stimulated fingers” (p. 378).  “Thus practice may expand synaptic connections by strengthening the effectiveness of existing connections” (p. 1483).  “Dramatic changes in afferent connections can also occur because of disuse” (p. 378) |
| 23 | The nervous system influences and is influenced by all other body systems (e.g., cardiovascular, endocrine, gastrointestinal and immune systems) | Structure/organization of the nervous system  Motor systems  Sensory systems | 45 | Building on this idea, in the 1930s Walter B. Cannon introduced the concept of homeostasis to describe the mechanisms that maintain within a narrow physiological range the constancy of composition of the bodily fluids, body temperature, blood pressure, and other physiological variables. ... All homeostatic behavior, including control of circulation, arises from neural modulation of the physiological properties of organ systems, mediated by hypothalamic control of the autonomic motor system and the endocrine system (p. 1057).  Relevant details added from the literature:  “Sensory neurons carry information from the body’s peripheral sensors into the nervous system …” (Kandel et al. 2013, p. 24).  “Motor neurons carry commands from the brain or spinal cord to muscles or glands …” (Kandel et al. 2013, p. 24).  “The sympathetic ganglia lie close to the spinal column and supply virtually every tissue in the body” (Kandel et al. 2013, p. 1059). |
| 24 | A properly functioning nervous system requires support from other types of cells, particularly glial cells | Structure/organization of the nervous system | 52 | The principle that embryonic neurons and glia arise from multipotential progenitors also applies to neurons born in adults (p. 1297).  Relevant details added from the literature:  “There are two main classes of cells in the nervous system: nerve cells, or neurons, and glial cells, or glia”. (p. 20).  “Glia Form the Insulating Sheaths for Axons” (p. 88).  “Astrocytes Support Synaptic Signaling” (p. 88). |
