# Supplementary material for: Identifying knowledge important to teach about the nervous system in the context of secondary biology and science education–A Delphi study
Source: PLoS One. 2021 Dec 21;16(12):e0260752. doi: 10.1371/journal.pone.0260752 (PMC8691623; doi:10.1371/journal.pone.0260752)
Supplement: S5 Table — The table shows an overview of all responses from all experts to each of the statements in all rounds. The letters denote different experts, but each letter does not necessarily denote the same experts across rounds. (DOCX) [file pone.0260752.s005.docx]

**S5 Table. Responses from experts in all rounds.**

The table shows an overview of all responses from all experts to each of the statements in all rounds. The letters denote different experts. Each letter does not necessarily denote the same experts across rounds.

Round one

| **Premade statement 1** | |
| --- | --- |
| Neurons are the elementary building blocks and signaling elements of the nervous system | |
|  | |
| Comments from experts | |
| A | I am not so comfortable with this point |
| B | “Signaling elements” does not sound good |
| C | Ok. But glia are also intimately involved in signaling processes. The student might think the brain only has neurons |
| D | Ok |
| E | Ok |
| F | Ok |
| G | Ok |
| H | Ok |
| I | Ok |
| J | Ok |
| K | Ok |
| L | Ok |
| M | Ok |
| N | Ok |
| O | Ok |

| **Premade statement 2** | |
| --- | --- |
| Neurons consist of three main structures: a cell body which is the metabolic center of the cell, dendrites which receive signals from other cells, and an axon with branches that sends signals to other cells | |
|  | |
| Comments from experts | |
| A | I am not so comfortable with this point |
| B | Ok |
| C | Ok |
| D | Ok |
| E | Ok |
| F | Use another word than “metabolic center” |
| G | Ok |
| H | Ok |
| I | Ok |
| J | Ok |
| K | Not all neurons have all these parts as you know... more over many neurons could receive and communicate through dendrites... |
| L | Ok |
| M | Ok |
| N | Ok |
| O | Ok |

| **Premade statement 3** | |
| --- | --- |
| The nervous system can be organized into three types of neurons: a) Sensory neurons which bring information to the nervous system, b) Motor neurons which send information to muscles and glands, and c) Interneurons which send information between neurons. | |
|  | |
| Comments from experts | |
| A | Ok |
| B | «interneurons send information between other neurons» is not good |
| C | This is similar to Per Brodal’s definition in his book. I think it can be confusing. For instance, it would mean a hippocampal pyramidal cell is one of many interneurons in the brain. I would normally only use interneurons to when referring to local circuit usually GABAergic neurons  Maybe you should write “… bring information to the central nervous system” |
| D | Ok |
| E | I would change this to say that interneurons “modulate information flow between neurons”. Some elaborate circuits have been identified for interneuron-to-interneuron connections (which can lead to disinhibition, for example), which I don’t think are captured by the wording as it is. Also- neurons send information to each other directly, and do not necessarily rely on interneurons for this purpose |
| F | What about principal neurons in «the rest of the brain», which is not primary sensory or motor areas? I think this point has been too simplified; we have principal cells (main processing units, excitatory) and interneurons (regulate the activity of the principal cells, inhibitory). Perhaps you can say that principal neurons can be divided into sensory / input cells, motor / output cells and “processing cells” or something like that, but I would have to think more to find ideal terms. I understand that this gets more complex, but something here needs to be changed |
| G | Ok |
| H | Ok |
| I | Too much simplified |
| J | Ok |
| K | I would add here a new category, principle/projections neurons too, if you talk about interneurons the upper is a must |
| L | Ok |
| M | becomes too simple - what is the definition of an interneuron? In the past it was whether it was inhibitory or not - if so - we have shown that they even project out of their structure - so I had been careful to defining |
| N | We usually distinguish between "principal neurons" and "interneurons", right? Or "Projection neurons" and "interneurons" where principal / projection sends messages between circuits / brain regions and interneurons send signals within the circuit?  Maybe specify what classification into 3 types is based on (where they send information to and from?) For example: Another division of neurons is in excitatory and inhibitory. A third is perhaps based on morphology? Etc… |
| O | Ok |

| **Premade statement 4** | |
| --- | --- |
| The nerve signal is an electrochemical signal that carries information | |
|  | |
| Comments from experts | |
| A | Ok |
| B | Electrochemical is difficult to understand |
| C | Ok |
| D | Ok |
| E | Ok |
| F | Does «electrochemical signal» mean anything for school students, or should it be explained briefly?  Is it confusing to state that the signal “carries” information (I imagine a signal that consists of a written letter or something), even if the term may be correct? |
| G | Ok |
| H | Formulation? |
| I | Ok |
| J | Ok |
| K | Ok |
| L | Ok |
| M | Ok |
| N | Maybe rather say that neurons communicate via a combination of electrical and chemical signals? |
| O | Ok |

| **Premade statement 5** | |
| --- | --- |
| The nerve signal consists of three main types of signals: 1) graded electrochemical signals which mainly occur in the dendrites, 2) all-or-none electrochemical signals (action potentials) which mainly occur in the axon, and 3) graded chemical signals which occurs between neurons. | |
|  | |
| Comments from experts | |
| A | Ok |
| B | Ok |
| C | Ok |
| D | Ok |
| E | Ok |
| F | Ok |
| G | Ok |
| H | The term «electrochemical» is confusing. Action potentials and synaptic potentials are pure electrical signals…  Reformulate |
| I | Ok |
| J | Ok |
| K | Ok |
| L | Maybe it makes sense to split “electrochemical” into “electrical” and “chemical” and be explicit where the signal is electrical vs. chemical. The post-synaptic potential (which you call “graded signal in the dendrites” is electrical and so are APs while the transfer between two neurons via the synapse is (mostly) chemical |
| M | Ok |
| N | This is correct. Some nervous systems are based only on gap junctions, and they probably exist with us as well, so there is a "different type" nervous signal that can be another basic principle. (But maybe a detail in this context). |
| O | Ok |

| **Premade statement 6** | |
| --- | --- |
| Action potentials are the signals by which the brain receives, analyzes, interprets, and conveys information. | |
|  | |
| Comments from experts | |
| A | Ok |
| B | Replace the words «analyzes, interprets» |
| C | Ok |
| D | Ok |
| E | Ok |
| F | Ok |
| G | Reformulate: Action potentials are the signals by which neurons communicate with each other |
| H | I would rather say that action potentials are used to convey information over long distances |
| I | Ok |
| J | Ok |
| K | Ok |
| L | Ok |
| M | Ok |
| N | Ok |
| O | Ok |

| **Premade statement 7** | |
| --- | --- |
| Inside neurons, the nerve signal flows in one direction only: from the dendrites to the axon terminals | |
|  | |
| Comments from experts | |
| A | Ok |
| B | «inside neurons» is not correct |
| C | Ok |
| D | Ok |
| E | I would also modify the wording on this one—the nerve signal flows *primarily* in one direction, but not only one direction. There are many papers out on back-propagating action potentials, and they can have a large effect on dendritic excitability. |
| F | Ok |
| G | Ok |
| H | If you want to simplify, yes – but it is not the case |
| I | Ok |
| J | Ok |
| K | As you know this is not true.. back propagating Aps, Neurotransmitter releasing dendrites, etc ... |
| L | Ok |
| M | Ok |
| N | Ok |
| O | Ok |

| **Premade statement 8** | |
| --- | --- |
| A neuron sends a nerve signal to other neurons only if the sum of its received signals is above a certain threshold. | |
|  | |
| Comments from experts | |
| A | Ok |
| B | Ok |
| C | Ok |
| D | Ok |
| E | Ok |
| F | Ok |
| G | Ok |
| H | Ok |
| I | Ok |
| J | Ok |
| K | Some sensory neurons, e.g. taste or photo receptors this is not true... |
| L | Make clear that it is a «weighted sum», i.e., that each incoming signal can contribute with different strength to the accumulated potential, depending on how strong the synapse is (and, in fact, the sign of the signal, i.e., whether it is excitatory or inhibitory). The formulation “sum of received signals” sounds like counting how many inputs there are |
| M | Ok |
| N | Ok |
| O | Ok |

| **Premade statement 9** | |
| --- | --- |
| Nerve signals pass from one neuron to the next at specialized zones called synapses | |
|  | |
| Comments from experts | |
| A | Ok |
| B | Ok |
| C | Ok |
| D | Ok |
| E | Ok |
| F | Ok |
| G | Ok |
| H | Ok |
| I | Ok |
| J | Ok |
| K | Ok |
| L | Ok |
| M | Ok |
| N | When I first read it, I thought it sounded like neurons were connected directly. See that it doesn't need to be read like that. At the same time, can it be okay to emphasize that nerve cells are separate? Maybe something like this: Nerve signals pass from one neuron to the next in specialized zones called synapses. Or.. Neurons are connected to each other or to other cell types in specialized zones called synapses where the nerve signal can pass. |
| O | Ok |

| **Premade statement 10** | |
| --- | --- |
| At synapses, the nerve signal is transmitted from one cell to the next either by diffusion of molecules over a tiny gap (chemical synapses) or by electrical current (electrical synapses) | |
|  | |
| Comments from experts | |
| A | Ok |
| B | Ok |
| C | Ok |
| D | Ok |
| E | And electrical synapses take place through direct cell-cell contact- no synaptic gap. |
| F | Include that the electrical signal is *converted to a chemical signal* at chemical synapses?  Reformulate |
| G | Ok |
| H | Ok |
| I | Ok |
| J | Ok |
| K | Ok |
| L | Ok |
| M | Ok |
| N | Ok |
| O | Ok |

| **Premade statement 11** | |
| --- | --- |
| At chemical synapses, the nerve signal is transmitted in one direction only: from the axon terminals of the presynaptic cell to the dendrites of the postsynaptic cell | |
|  | |
| Comments from experts | |
| A | Ok |
| B | Ok |
| C | Ok |
| D | Ok |
| E | Change to: … “transmitted primarily in one direction” |
| F | Ok |
| G | Ok |
| H | Ok |
| I | Ok |
| J | Ok |
| K | Ok |
| L | Ok |
| M | Ok |
| N | Ok |
| O | Ok |

| **Premade statement 12** | |
| --- | --- |
| There are two main functional types of chemical synapses: Excitatory synapses which increase the probability of the postsynaptic neuron to send an action potential, and 2) Inhibitory synapses which decrease the probability of the postsynaptic neuron to send an action potential | |
|  | |
| Comments from experts | |
| A | Ok |
| B | Ok |
| C | Ok |
| D | Ok |
| E | Ok |
| F | Ok |
| G | Would have included the principle of gas and brake, and mentioned that without inhibition one would get too much activity => epilepsy |
| H | Ok |
| I | Ok |
| J | Ok |
| K | Ok |
| L | Ok |
| M | Ok |
| N | Ok |
| O | Ok |

| **Premade statement 13** | |
| --- | --- |
| A neuron receives both excitatory and inhibitory nerve signals from other cells, but can only send one type of signal, excitatory or inhibitory, to all its postsynaptic cells, not both. | |
|  | |
| Comments from experts | |
| A | Ok |
| B | Ok |
| C | Ok |
| D | Ok |
| E | Ok |
| F | Ok |
| G | Ok |
| H | Ok |
| I | Ok |
| J | Ok |
| K | This concept is widely disproven by several neuron types co-releasing GABA/Glutamate, even acetylcholine/glutamate |
| L | Ok |
| M | Ok |
| N | Ok |
| O | Ok |

| **Premade statement 14** | |
| --- | --- |
| Synapses are formed by genetic programs during embryonic development but are modified through interactions with the internal and external environment | |
|  | |
| Comments from experts | |
| A | Ok |
| B | Ok |
| C | Ok |
| D | Ok |
| E | Ok |
| F | Ok |
| G | Ok |
| H | Ok |
| I | Ok |
| J | Ok |
| K | Ok |
| L | There is synaptogenesis throughout life, not only during embryonic development |
| M | Ok |
| N | Ok |
| O | Ok |

| **Premade statement 15** | |
| --- | --- |
| The effectiveness of synapses changes as we learn new things, and these changes are necessary to form memories. | |
|  | |
| Comments from experts | |
| A | Ok |
| B | Ok |
| C | Ok |
| D | Ok |
| E | Ok |
| F | Ok |
| G | Ok |
| H | Ok |
| I | Ok |
| J | Ok |
| K | Ok |
| L | That’s similar as 14, maybe combine? |
| M | here I would like to add that memories are not only effective synapses but which synapses carry the engram (which pattern) - Ensembles of nerve cells - but becomes complicated. Just wanted to avoid that the more efficient the better the memory - it's wrong |
| N | Ok |
| O | Ok |

| **Premade statement 16** | |
| --- | --- |
| Each neuron in the central nervous system receives information from many other neurons and sends information to many other neurons to form networks and share information | |
|  | |
| Comments from experts | |
| A | Ok |
| B | Ok |
| C | Ok |
| D | Ok |
| E | Ok |
| F | Indicate how many “many” can be? I don’t remember exactly, but we talk tens of thousands, don’t we? |
| G | Ok |
| H | Ok |
| I | Ok |
| J | Ok |
| K | Ok |
| L | Ok |
| M | Ok |
| N | Ok |
| O | Ok |

| **Premade statement 17** | |
| --- | --- |
| Each neuron makes specific connections with certain postsynaptic target cells but not with others | |
|  | |
| Comments from experts | |
| A | Ok |
| B | Ok |
| C | Ok |
| D | Ok |
| E | Ok |
| F | “..but not with others” is a bit unclear. Consider to add “…so that its signal is passed on to a selected group of neurons rather than to all its neighbours”…? |
| G | Ok |
| H | Ok |
| I | Ok |
| J | Ok |
| K | Ok |
| L | Ok |
| M | Ok |
| N | Ok |
| O | Ok |

| **Premade statement 18** | |
| --- | --- |
| Our perceptions, thoughts, feelings and behavior are mainly determined by which pathways the nerve signal takes through the network of neurons | |
|  | |
| Comments from experts | |
| A | Ok |
| B | Not sure about thoughts, feelings and behavior |
| C | Ok |
| D | Ok |
| E | I would rather say… “driven by nerve signals coursing through specific pathways connecting networks of neurons.”. The way it is worded now, it sounds like one neural pathway is for a perception, another one is for a thought, and yet another is for feelings—while it’s becoming increasingly clear in the field that these pathways and functions rather impossible to separate from one another.  I would say that there are separate processing pathways in the brain, but they overlap, and there is way more cross-talk between them than previously appreciated in the classical textbook view.  I think it is totally correct to say that there are major processing pathways in the brain for perceptions, feelings (internal states), and behavior (i.e. generating motor behavior), and that these pathways interact and influence one another |
| F | Internal states and non-motor functions: not only anatomical pathways, very much about activity patterns |
| G | Ok |
| H | Nor quite sure about thoughts |
| I | Ok |
| J | Ok |
| K | Ok |
| L | This sounds too linear for my taste. It is not one signal that “finds its path” through the network but many recurring and cross-talking signals that are all transmitted and initiated at the same time that give rise to our perceptions etc |
| M | Think it becomes too simple - there are both top-down and bottom-up processes anyway |
| N | Ok |
| O | Ok |

| **Premade statement 19** | |
| --- | --- |
| The intensity of our perceptions and actions are mainly determined by the frequency of action potentials elicited by the sensory and motor neurons, respectively. High frequency gives rise to high intensity and low frequency to low intensity. | |
|  | |
| Comments from experts | |
| A | This statement is maybe not so central? |
| B | Reformulate. It sounds like it is like this in the whole brain |
| C | Ok |
| D | Ok |
| E | Ok |
| F | Is this really the case, enough to be a general rule? Perhaps in sensory information, but the statement gives the impression that a higher firing rate always codes for “more” of something, which is not the case - ? |
| G | Ok |
| H | Not sure about that… |
| I | Becomes too simplified |
| J | Ok |
| K | Not photo receptors as you know |
| L | I would add “(rate code)” and also say that spike-timing of single APs also can carry information |
| M | Do not completely agree |
| N | Ok |
| O | Ok |

| **Premade statement 20** | |
| --- | --- |
| The brain has a continuous self-sustaining activity. Sensory input cannot stop or start this activity, only modify it | |
|  | |
| Comments from experts | |
| A | Ok |
| B | Ok |
| C | Ok |
| D | Ok |
| E | Ok |
| F | Nice point. Should you somewhere mention how it may be modified? |
| G | Ok |
| H | Ok |
| I | Ok |
| J | Ok |
| K | Ok |
| L | Ok |
| M | Seems too simple, but have no arguments against it |
| N | Ok |
| O | Ok |

| **Premade statement 21** | |
| --- | --- |
| The brain has distinct regions that are specialized for different functions, like perception, movement, language, thought, emotions, etc. However, different brain regions are interlinked, and proper brain function requires coordinated action of neurons in many brain regions | |
|  | |
| Comments from experts | |
| A | Ok |
| B | Ok |
| C | Ok |
| D | Ok |
| E | Ok |
| F | Nice point. Consider exchanging “proper brain function” with “each function”. |
| G | Ok |
| H | Hm… no brain area is dedicated to thoughts… |
| I | Ok |
| J | Ok |
| K | Ok |
| L | Ok |
| M | Ok |
| N | Think it is controversial to say that thoughts are localized in one area. |
| O | Ok |

| **Premade statement 22** | |
| --- | --- |
| Brain structure and function is maintained by regularly challenging the brain with physical and mental activity – “use it or lose it” | |
|  | |
| Comments from experts | |
| A | I would like to see a statement about the plasticity of the nervous system, possibly in relation to “use it or lose it” |
| B | Ok |
| C | Ok |
| D | Ok |
| E | Ok |
| F | Nice point, but perhaps «maintain” is a bit strong – may sound like if you sit in the sofa your brain will actually degenerate. Would “sustain” be a better shade? |
| G | Ok |
| H | Ok |
| I | Ok |
| J | Ok |
| K | “use it or lose it”? |
| L | That is maybe a bit unspecific and may lead to wrong conclusions. It is certainly not true for everything. For example, we don’t usually forget how to ride a bicycle (or speak our mother tongue) even though we don’t use them. So either that point must be more specific or reformulated to make it true |
| M | Ok |
| N | True, the "use it or lose it" principle is also more specific than just "mental activity" in many cases (and mistakes in others - such as strong emotional memories? |
| O | “Maintain” might not be the right word here? |

| **Premade statement 23** | |
| --- | --- |
| The nervous system influences and is influenced by all other body systems (e.g., cardiovascular, endocrine, gastrointestinal and immune systems) | |
|  | |
| Comments from experts | |
| A | Ok |
| B | Ok |
| C | Ok |
| D | Ok |
| E | Ok |
| F | Ok |
| G | Ok |
| H | Ok |
| I | Ok |
| J | Ok |
| K | Motor |
| L | Ok |
| M | Ok |
| N | Ok |
| O | Ok |

| **Premade statement 24** | |
| --- | --- |
| A properly functioning nervous system requires support from other types of cells, particularly glial cells. | |
|  | |
| Comments from experts | |
| A | Ok |
| B | Ok |
| C | Ok |
| D | Ok |
| E | Ok |
| F | I would say that glia is part of the nervous system, which consist of neurons and glia. Perhaps you can rather say “neurons require support from other types of cells, particularly glial cells, which together constitute the nervous system.”. The nervous system consists of both neurons and glia. Perhaps this statement could come earlier, where you introduce neurons? |
| G | Ok |
| H | Ok |
| I | Ok |
| J | Ok |
| K | Ok |
| L | Ok |
| M | Ok |
| N | Okay, but you haven't said what glial cells are, so it might not provide that much extra information here. Can turn it around and say that the nervous system is dependent on several other types of cells, such as… Or say something about glial cells, maybe also that they may be involved in signal processing |
| O | Ok |

**Additions suggested by the experts:**

| **Expert** | **Addition 1: Add neurotransmitter systems** |
| --- | --- |
| L | I would add something about neurotransmitter systems (dopamine, norepinephrine) as those have a strong influence on how the brain processes everything. |

| **Expert** | **Addition 2: Add interaction between networks** |
| --- | --- |
| E | I would say that there are separate processing pathways in the brain, but they overlap, and there is way more cross-talk between them than previously appreciated in the classical textbook view |
| L | Synchronized activity between neurons is a key organizing feature of the brain (e.g., theta- and gamma oscillations). |

| **Expert** | **Addition 3: Add large scale networks** |
| --- | --- |
| L | The brain flexibly engages distributed large-scale networks to adapt to changing requirements (keyword resting-state networks, e.g., default-mode network) |

| **Expert** | **Addition 4: Add the major divisions of the nervous system** |
| --- | --- |
| G | The general parts of the nervous system are not well covered. Differences between the voluntary and the autonomous nervous systems are lacking. |
| J | Perhaps a good idea to also include the concept of the division of the nervous system into the central nervous system (brain, spinal cord) and the peripheral nervous system. |

| **Expert** | **Addition 5: Add that glial cells are part of the nervous system** |
| --- | --- |
| F | Include that “neurons require support from other types of cells, particularly glial cells, which together constitute the nervous system.”. |
| J | Include that the nervous system contains two main cell types: nerve cells and glial cells |

| **Expert** | **Addition 6: Add that neurons and muscles are excitable cells** |
| --- | --- |
| J | Neurons (and muscle cells) are excitable cells, cells that can actively change the electric potential across the cell membrane as a means to carry information” |

| **Expert** | **Addition 7: Add mor information about plasticity** |
| --- | --- |
| A | I would like to see a statement about the plasticity of the nervous system, possibly in relation to “use it or lose it” |
| N | Include plasticity = physical changes in the brain, neurogenesis, metabolism/sleep |

Round two

**Gross anatomy and function**

| **Statement 1** | | | Agree | Neutral | Disagree |
| --- | --- | --- | --- | --- | --- |
| The nervous system is commonly divided into the central nervous system (CNS) which consists of the brain and the spinal cord, and the peripheral nervous system (PNS) which connects the CNS with the rest of the body. | | | 7 | 1 |  |
|  | | | | | |
| Comments from the experts: | | | | | |
| Agree: | F | What you say is correct. However, relevant information about the elements forming the PNS is lacking. But OK, I see that you define the PNS below | | | |
| Neutral: | C | Consider to add “…through peripheral nerves” or something like that. If one knows nothing about the nervous system, it may sound mysterious how the PNS connects the CNS to the rest of the body? | | | |
|  | | | | | |

| **Statement 2** | | | Agree | Neutral | Disagree |
| --- | --- | --- | --- | --- | --- |
| The PNS is commonly divided into a sensory system which brings information from the sense organs to the CNS, and a motor system which brings information from the CNS to muscles and glands | | | 7 | 1 |  |
|  | | | | | |
| Comments from the experts: | | | | | |
| Agree: | B | And the autonomic nervous system. Don't forget that one! | | | |
|  | C | Is the  “…and glands” part (and “muscles” probably including both skeletal and smooth muscles) supposed to cover the autonomous nerve system? If so, agree. Could perhaps been clearer but understand that then it will be complicated | | | |
| Neutral: | F | Yes, I know it is often classified like this. However, for me it would be logic to categorize into sensory part, motoric part, and autonomic (sympathetic and parasympathetic) | | | |
|  | | | | | |

| **Statement 3** | Agree | Neutral | Disagree |
| --- | --- | --- | --- |
| The nervous system influences and is influenced by all other body systems (e.g., cardiovascular, exocrine- and endocrine glands, gastrointestinal, muscular and immune systems). | 8 |  |  |
|  | | | |
| No comments from the experts: I have merged this principle with the content of principle 2 because they supported each other anatomically and functionally. This is now principle 3. | | | |

| **Statement 4** | | | Agree | Neutral | Disagree |
| --- | --- | --- | --- | --- | --- |
| The brain has a continuous self-sustaining activity. Sensory input cannot stop or start this activity, only modify it. | | | 6 | 2 |  |
|  | | | | | |
| Comments from the experts: | | | | | |
| Agree: | B | You might want to add that this is especially true for the brainstem vital systems. | | | |
| Neutral: |  |  | | | |
|  | | | | | |

| **Statement 5** | | | Agree | Neutral | Disagree |
| --- | --- | --- | --- | --- | --- |
| Regularly challenging the brain with physical and cognitive activity helps sustain brain structure and function. | | | 7 | 1 |  |
|  | | | | | |
| Comments from the experts: | | | | | |
| Agree: | B | These can even enhance brain structure and function, not just sustain them | | | |
| Neutral: |  |  | | | |
| Disagree: | C | I agree about the principle, but not the exact phrasing. Perhaps I would prefer something like “exercising” instead of “challenging” | | | |
|  | | | | | |

**Cell type, structure and function**

| **Statement 6** | | | Agree | Neutral | Disagree |
| --- | --- | --- | --- | --- | --- |
| 1. The nervous system consists of three major classes of cells: 2. Sensory cells, which transform stimulus energy (light, sound, temperature, chemicals, etc.) into nerve signals and pass them on to neurons 3. Neurons, which receive, process and send nerve signals to each other, or to effector organs like muscles and glands 4. Glial cells, which protect and support neuron functioning.   Each class can be subdivided further based on several criteria, f. ex. morphology, physiology, location and gene expression | | | 5 | 1 | 1 |
|  | | | | | |
| Comments from the experts: | | | | | |
| Agree: | H | Maybe under a) change 'nerve signals' to 'electrical signals'? It seems you want to point out that sensory cells not necessarily are neurons, in that case, it might be strange that non-nerve cells process nerve signals | | | |
| Neutral: | I | Aren't sensory cells also neurons? | | | |
| Disagree: | C | Perhaps agree but needs clarification before I do. I have never seen this type of division before, with “sensory cells” as a third type of nervous-system-cell next to neurons and glia. How are they normally classified? But I am not an expert on PNS-stuff! If accepted by the others, fine by me! Another thing, does “morphology” mean anything to school pupils? | | | |
|  | | | | | |

| **Statement 7** | | | Agree | Neutral | Disagree |
| --- | --- | --- | --- | --- | --- |
| A neuron commonly consists of three main structures: dendrites which receive nerve signals from several other cells, a single branching axon which sends signals to several other cells, and a cell body which maintains the cell machinery. | | | 6 | 1 |  |
|  | | | | | |
| Comments from the experts: | | | | | |
| Agree: | C | Is “cell machinery” informative for students? | | | |
| Neutral: | I | Dendrites can also release neurotransmitters | | | |
| Disagree: |  |  | | | |
|  | | | | | |

**The nerve signal**

| **Statement 8** | | | Agree | Neutral | Disagree |
| --- | --- | --- | --- | --- | --- |
| 1. The nerve signal is dynamic and consists of three main types of impulses: 2. Graded electrical impulses which flow a short distance primarily from the dendrites to the beginning of the axon 3. All-or-none electrical impulses (action potentials) which flow a long distance primarily from the beginning of the axon to the axon terminals. 4. Graded pulses of chemicals which primarily flow from the axon terminals, over a tiny extracellular gap, to the dendrites of the receiving neuron | | | 6 | 1 |  |
|  | | | | | |
| Comments from the experts: | | | | | |
| Agree: | B | Some functional cell types (e.g. mechano-electrical hair cells) don’t generate action potentials, while a classic cortical pyramidal neuron does. | | | |
|  | H | Nice principle. With respect to c), I am not sure if ' Graded pulses of chemicals' describes the process most accurately. Maybe exchange 'pulses' with 'amounts'? E.g. " Graded amounts of chemicals are released from the axon terminal and these chemicals primarily flow over a tiny extracellular gap to the dendrites of the receiving neuron" | | | |
| Neutral: |  |  | | | |
| Disagree: | C | Neutral and Disagree. It should be more clear that a is much smaller in amplitude than b and happens within single neurons, and that integration of a’s decides whether b is evoked, which conveys signals to other neurons. Or make it more clear how this connects to principle 9. C is more complicated, given that synaptic release of chemicals is just one form of chemical signaling in the brain. What about volume transmission of neurotransmitters, and “local” signals which act other places than across one synaptic gap? I understand that you cannot explain all this, but perhaps C should be formulated in a bit more open way, which at least opens for other forms of transmission (I can see that “primarily” opens a little bit up for that, but could it be more?) | | | |
|  | | | | | |

| **Statement 9** | | | Agree | Neutral | Disagree |
| --- | --- | --- | --- | --- | --- |
| The graded pulses of chemicals make the receiving neurons generate graded electrical impulses. Graded electrical impulses larger than a certain voltage value (threshold) make neurons send action potentials. Action potentials entering the axon terminal make neurons send graded pulses of chemicals. | | | 5 | 2 |  |
|  | | | | | |
| Comments from the experts: | | | | | |
| Agree: | B | “graded pulses of chemicals” is indeed good wording | | | |
|  | H | Should you include that neurotransmitters have both negative and positive effects on the postsynaptic neuron? It is also a bit confusing in this principle that the two first statements refer to the input, integration dendritic side, whereas the last statement refers to the output, presynaptic terminal side. | | | |
| Neutral: | E | Reword? | | | |
|  | C | Not a bad way to meet the comments I had to 8, in an efficient way. Still some issues are bugging me: chemicals not only work on ion channels (making el impulses) but also on intracellular molecular cascades (metabotropic receptors), which is pretty important. Is that mentioned anywhere, for example in relation to different types of / neuromodulatory transmittors? | | | |
|  | C | The last sentence: action potentials send graded pulses of chemicals? I don’t know enough about this, but if an AP is all or nothing, won’t that lead to an all-or-nothing release, though that may be modified by other states / local factors? The problem in the statement is that it is hard to understand how an all-or-nothing response possibly can lead to a graded signal without taking into account other things that may be going on? | | | |
| Disagree: |  |  | | | |
|  | | | | | |

| **Statement 10** | | | Agree | Neutral | Disagree |
| --- | --- | --- | --- | --- | --- |
| The graded pulses of chemicals pass from one neuron to the next at specialized zones called synapses. | | | 6 | 1 |  |
|  | | | | | |
| Comments from the experts: | | | | | |
| Agree: | C | Should this statement come under the block called “synapses”? | | | |
| Neutral: | E | Combine with 9? | | | |
| Disagree: |  |  | | | |
|  | | | | | |

**Synapses**

| **Statement 11** | | | Agree | Neutral | Disagree |
| --- | --- | --- | --- | --- | --- |
| 1. There are two main functional types of synapses: 2. Excitatory synapses which increase the probability of the receiving neuron to reach threshold for sending an action potential 3. Inhibitory synapses which decrease the probability of the receiving neuron to reach threshold for sending an action potential | | | 7 |  |  |
|  | | | | | |
| Comments from the experts: | | | | | |
| Agree: | I | These are chemical synapses.... what about electrical synp. It might be wise to keep electrical synapses out, if you wish to keep it simple | | | |
|  | C | Except that my first thought on “two types of synapses” is electrical (gap junctions) vs chemical (transmittors) synapses. If possible, you could reformulate to something that states that two types of synapeses are BLAH, instead of making it sound like this is the only logical separation? But a detail. | | | |
| Neutral: |  |  | | | |
| Disagree: |  |  | | | |
|  | | | | | |

| **Statement 12** | | | Agree | Neutral | Disagree |
| --- | --- | --- | --- | --- | --- |
| 1. A neuron receives signals through both excitatory and inhibitory synapses but can primarily send signals through only one of the types, excitatory or inhibitory, to all its target (postsynaptic) cells. Consequently, most neurons can be physiologically classified as either excitatory or inhibitory based on the effect they have on their postsynaptic cells. | | | 5 | 2 |  |
|  | | | | | |
| Comments from the experts: | | | | | |
| Agree: |  |  | | | |
| Neutral: | E | Too much detail? | | | |
|  | I | True to some extent, as now there are more examples of co-release | | | |
|  | C | Good – If it is the case that we later will hear a little bit more about different transmitters / neuromodulators | | | |
| Disagree: |  |  | | | |
|  | | | | | |

| **Statement 13** | | | Agree | Neutral | Disagree |
| --- | --- | --- | --- | --- | --- |
| 1. Each neuron makes synapses with certain target cells but not with others so that its nerve signal is send to a selected group of neurons rather than to all its neighbors. | | | 7 |  |  |
|  | | | | | |
| Comments from the experts: | | | | | |
| Agree: | E | Check spelling | | | |
|  | B | How about this for the wording: "Each neuron makes synapses with specific target cells, not with every cell around them. Thus, a signal from a given neuron will only go to a selected group of target neurons rather than to all its neighbors." | | | |
| Neutral: |  |  | | | |
| Disagree: |  |  | | | |
|  | | | | | |

**Neural plasticity**

| **Statement 14** | | | Agree | Neutral | Disagree |
| --- | --- | --- | --- | --- | --- |
| 1. Synapses are formed and modified throughout life as a result of genetic programs and interactions with the environment. | | | 5 | 1 | 1 |
|  | | | | | |
| Comments from the experts: | | | | | |
| Agree: |  |  | | | |
| Neutral: | H | This seems a bit unspecific. To me it reads that the synapses themselves are 'interacting with the environment'. Maybe you can rephrase this statement to make it more clear? | | | |
| Disagree: | E | I would omit the genetic programs | | | |
|  | | | | | |

| **Statement 15** | | Agree | Neutral | Disagree |
| --- | --- | --- | --- | --- |
| 1. Synaptic modifications increase or decrease the effectiveness of signaling between neurons. | | 7 |  |  |
|  | | | | |
| Comments from the experts: | | | | |
| Agree: |  | | | |
| Neutral: |  | | | |
| Disagree: |  | | | |
|  | | | | |

| **Statement 16** | | | Agree | Neutral | Disagree |
| --- | --- | --- | --- | --- | --- |
| 1. When you learn, memories are stored as modified synapses in the network of neurons involved with the learning activity (sensory network, motor network, emotional network, etc.). | | | 6 |  | 1 |
|  | | | | | |
| Comments from the experts: | | | | | |
| Agree: | C | Good, though perhaps not needed to add the stuff in the parenthesis, which just opens up doors to difficult terrains. Perhaps the principle is clearer without that. | | | |
| Neutral: |  |  | | | |
| Disagree: | E | Philosophical issue – I don’t think you need to assume cognitive structures/ memories to explain learning | | | |
|  | | | | | |

| **Statement 17** | | | Agree | Neutral | Disagree |
| --- | --- | --- | --- | --- | --- |
| The specificity of a memory is determined by which synapses are modified, in which way each of them are modified (increased or decreased effectiveness), and the magnitude of the modifications. | | | 5 |  | 2 |
|  | | | | | |
| Comments from the experts: | | | | | |
| Agree: | B | If you can use the phrase, "a distributed pattern of synaptic weights", that one stuck with me from before graduate school. | | | |
| Neutral: |  |  | | | |
| Disagree: | C | I found this one difficult. I think I understand what you want to say, but what exactly does “the specificity of a memory” means? Is this one needed? Quite advanced anyway, perhaps 16 is sufficient. | | | |
|  | | | | | |

**Neural coding**

| **Statement 18** | | | Agree | Neutral | Disagree |
| --- | --- | --- | --- | --- | --- |
| Our perceptions, thoughts, feelings and behaviors are mainly determined by two factors:   1. the specific pathways the nerve signals take through the network of neurons 2. the frequency pattern of action potentials as they travel through these pathways. | | | 5 | 1 | 1 |
|  | | | | | |
| Comments from the experts: | | | | | |
| Agree: | C | “Frequency pattern" is fine! However, if you are going to dig properly into it, then you might say that the "frequency pattern" leads to believe that it is rate code you mean, at least the frequency in a single neuron, whereas what I may miss a bit is the aspect of "timing", i. e. when the action potentials come in relation to other action potentials and other activity / states in the network. But it gets too heavy to bake in here, so I'm happy that the temporal aspect has been taken into account through the "frequency pattern" in 18b here, in addition to the spatial in a! | | | |
| Neutral: |  |  | | | |
| Disagree: | E | A touch reductionistic? | | | |
|  | | | | | |

| **Statement 19** | | | Agree | Neutral | Disagree |
| --- | --- | --- | --- | --- | --- |
| Specific pathways for different functions give rise to distinct brain regions that are specialized for movement, language, feelings, vision, audition, olfaction, etc. However, the brain regions are interlinked, and a proper function of each requires coordinated action of neurons across many regions | | | 7 |  |  |
|  | | | | | |
| Comments from the experts: | | | | | |
| Agree: | B | Another wording suggestion (and please take it or leave it as you like!). "Once mature, the brain consists of specific pathways for different functions. At larger scales, these pathways make up brain regions that are specialized for movement, language....." | | | |
| Neutral: | N | Is it the specific pathways that actually give rise to the distinct brain regions? | | | |
| Disagree: |  |  | | | |
|  | | | | | |

| **Statement 20** | | | Agree | Neutral | Disagree |
| --- | --- | --- | --- | --- | --- |
| 1. The network of neurons underlying our perceptions, feelings and behaviors interact in such a way that our perceptions influence how we feel and behave, our behavior influence what we perceive and how we feel, and our feelings influence how we behave and what we perceive. | | | 6 |  | 1 |
|  | | | | | |
| Comments from the experts: | | | | | |
| Agree: | B | Rewording: "The network of neurons underlying our perceptions, feelings and behaviors interact with each other all the time. This means that our perceptions influence how we feel and behave, but this goes both ways. Our behavior (i.e. what we are doing) also influences how we perceive and how we feel, and our feelings influence what we perceive and how we behave:" | | | |
|  | C | A hard one, it is attempting to include “think “ etc, but I get your point, I think this works. | | | |
|  | M | But what is the current status on consciousness? you probably should include it, but in a simple way. | | | |
| Neutral: |  |  | | | |
| Disagree: | E | I would omit the feelings. Spelling | | | |
|  | | | | | |

| **Statement 21** | | | Agree | Neutral | Disagree |
| --- | --- | --- | --- | --- | --- |
| In peripheral sensory neurons (touch, taste, smell, sound, light, etc.), high frequency of action potentials give rise to intense perceptions, and low frequency give rise to faint perceptions. | | | 4 | 3 |  |
|  | | | | | |
| Comments from the experts: | | | | | |
| Agree: | C | I would have had this one and 22 higher up in the more “concrete” stuff about neural coding, and let the later ones be the more abstract ones | | | |
| Neutral: | E | Spelling | | | |
|  | H | Is this only true for peripheral neurons? | | | |
|  | H | Here it seems that perception arises strictly because of the AP firing rate of sensory neurons. | | | |
| Disagree: |  |  | | | |
|  | | | | | |

| **Statement 22** | | | Agree | Neutral | Disagree |
| --- | --- | --- | --- | --- | --- |
| In peripheral motor neurons, high frequency of action potentials give rise to fast behavior and low frequency give rise to slow behavior. | | | 3 | 2 | 2 |
|  | | | | | |
| Comments from the experts: | | | | | |
| Agree: | C | I think this could be merged with the previous one, if possible | | | |
| Neutral: | B | Read up on intrafusal and extrafusal fibers where muscles connect with bone. There are fast and slow fibers at play there.... but they are sensory. Is the statement actually true? (i.e. is it really that simple?) | | | |
| Disagree: | E | What do you mean by fast/slow behavior? 21 and 22 are maybe not so central? Check spelling. | | | |
|  | H | Is this true? I suppose that high AP firing rate can give rise to more forceful movements, which do not necessarily need to be fast. Speed and contraction force is heavily influenced by the biochemical properties of the muscle fibers as well | | | |
|  | | | | | |

Round 3

**Gross anatomy and function**

| **Statement 1** | | | Agree | Neutral | Disagree |
| --- | --- | --- | --- | --- | --- |
| The vertebrate nervous system comprises the brain, the spinal cord, ganglia and bundles of fibers called nerves. The brain and the spinal cord make up the central nervous system (CNS), whereas ganglia and nerves make up the peripheral nervous system (PNS). The PNS connects the CNS with the rest of the body. | | | 5 |  |  |
|  | | | | | |
| Comments from the experts: | | | | | |
| Agree: | N | Maybe use a different word than “fibers”, and maybe ganglia is unnecessary | | | |
|  | M | The Enteric nervous system is under the umbrella of ganglia and nerves in this definition? | | | |
| Neutral: |  |  | | | |
| Disagree: |  |  | | | |
|  | | | | | |

| **Statement 2** | | | Agree | Neutral | Disagree |
| --- | --- | --- | --- | --- | --- |
| The nervous system is commonly divided into a volitional system (the somatic system) and a non-volitional system (the autonomous system). However, the two systems interact to support compatible physiological, emotional and behavioral responses. | | | 5 |  |  |
|  | | | | | |
| Comments from the experts: | | | | | |
| Agree: | M | Just a comment: philosophically speaking "volitional" and "non-volitional" are not so meaningful expressions as they imply some kind of control over neural processes that is beyond the physical realm (i.e. spiritual). I would rather say that processes in the somatic system involve conscious awareness | | | |
| Neutral: | H | Is there a better term to use than 'volitional' vs 'non-volitional'? | | | |
| Disagree: |  |  | | | |
|  | | | | | |

| **Statement 3** | | | Agree | Neutral | Disagree |
| --- | --- | --- | --- | --- | --- |
| 1. The volitional and the non-volitional system each have a sensory division carrying information about the surroundings into the central nervous system, and a motor division carrying information from the central nervous system to the surrounding tissue. Through this architecture, the nervous system influences and is influenced by all other body systems (e.g., cardiovascular-, gastrointestinal-, muscular-, immune-, exocrine- and endocrine systems). | | | 4 | 1 |  |
|  | | | | | |
| Comments from the experts: | | | | | |
| Agree: |  |  | | | |
| Neutral: | C | It could potentially sound confusing that you have "two sensory systems", autonomous and volitional, but I guess it is technically right? | | | |
| Disagree: |  |  | | | |
|  | | | | | |

| **Statement 4** | | | Agree | Neutral | Disagree |
| --- | --- | --- | --- | --- | --- |
| 1. The brain has a continuous self-sustaining activity. Sensory input cannot stop or start this activity, only modify it. | | | 4 | 1 |  |
|  | | | | | |
| Comments from the experts: | | | | | |
| Agree: |  |  | | | |
| Neutral: | M | I interpret this to mean that neurons in the brain show activity that does not require sensory inputs. Is it perhaps better to phrase it to say that this activity is not dependent on sensory inputs but can be modified by it? On the level of single cells and over short temporal scales, sensory inputs can both set of (start) spiking in recipient neurons as well as suppress (stop) spiking through inhibition. | | | |
| Disagree: |  |  | | | |
|  | | | | | |

**Cell types and functional units**

| **Statement 5** | | Agree | Neutral | Disagree |
| --- | --- | --- | --- | --- |
| 1. The nervous system consists of three major classes of cells: 2. Sensory cells, which transform stimulus energy (light, sound, temperature, chemicals, etc.) into nerve signals and pass them on to neurons 3. Neurons, which receive, process and send nerve signals to each other, or to effector organs like muscles and glands 4. Glial cells, which protect and support neuron functioning. | | 5 |  |  |
|  | | | | |
| Comments from the experts: | | | | |
| Agree: |  | | | |
| Neutral: |  | | | |
| Disagree: |  | | | |
|  | | | | |

| **Statement 6** | | | Agree | Neutral | Disagree |
| --- | --- | --- | --- | --- | --- |
| 1. A neuron usually consists of three main structures: 2. dendrites which receive nerve signals from several other cells 3. a single branching axon with many terminals, which sends nerve signals to several other cells 4. a cell body | | | 5 |  |  |
|  | | | | | |
| Comments from the experts: | | | | | |
| Agree: | M | As people probably have commented before there are of course exceptions, but that goes for most thing in biology | | | |
| Neutral: |  |  | | | |
| Disagree: |  |  | | | |
|  | | | | | |

**The nerve signal**

| **Statement 7** | | | Agree | Neutral | Disagree |
| --- | --- | --- | --- | --- | --- |
| 1. The typical nerve signal is generated by neurons. It is dynamic and consists of three main types of impulses: 2. Graded electrical impulses which primarily flow from the dendrites to the beginning of the axon 3. “All-or-none” electrical impulses (action potentials) which primarily flow from the beginning of the axon to the axon terminals. 4. Graded pulses of chemicals which primarily flow from the axon terminals, over an extracellular gap, to the dendrites of a receiving neuron. The chemical impulses propagate much slower than the electrical impulses. | | | 5 |  |  |
|  | | | | | |
| Comments from the experts: | | | | | |
| Agree: | M | Electrical synapses? | | | |
|  | C | Probably the most correct version (after bunch of critical feedback), but did it now become more complicated / confusing than what perhaps was your first draft? Hope not! ("Dynamic" in the first part may have to do with my comments, but may not be needed here?) | | | |
| Neutral: |  |  | | | |
| Disagree: |  |  | | | |
|  | | | | | |

| **Statement 8** | | | Agree | Neutral | Disagree |
| --- | --- | --- | --- | --- | --- |
| When a neuron receives graded pulses of chemicals, it generates graded electrical impulses. If the graded electrical impulses are larger than a certain voltage value (threshold), the neuron generates action potentials successively and with maximum frequency until the voltage value goes below threshold. When the action potentials reach the axon terminals, the neuron releases graded pulses of chemicals. | | | 3 | 1 |  |
|  | | | | | |
| Comments from the experts: | | | | | |
| Agree: | N | Maybe delete “… and with maximum frequency …”. Otherwise it will be difficult to understand the logic in principle 15 b. If you keep it, you need additional information to understand principle 15 b (the fluctuations around threshold of the graded electrical impulses). | | | |
| Neutral: | C | Would consider to remove "successively and with ... goes below threshold"? | | | |
| Disagree: |  |  | | | |
|  | | | | | |

**Connections between neurons**

| **Statement 9** | | | Agree | Neutral | Disagree |
| --- | --- | --- | --- | --- | --- |
| 1. The graded pulses of chemicals pass from one neuron to the next at locations called synapses which are specialized zones for communication between neurons. | | | 4 |  |  |
|  | | | | | |
| Comments from the experts: | | | | | |
| Agree: | C | (Well, then we have electrical synapses but I support if they are not included here!) | | | |
| Neutral: |  |  | | | |
| Disagree: |  |  | | | |
|  | | | | | |

| **Statement 10** | | | Agree | Neutral | Disagree |
| --- | --- | --- | --- | --- | --- |
| 1. There are two main functional types of synapses: 2. Excitatory synapses which make the receiving neurons generate action potentials or increase their probability to do so. 3. Inhibitory synapses which prevent the receiving neurons from generating action potentials or decrease their probability to do so. | | | 3 | 1 |  |
|  | | | | | |
| Comments from the experts: | | | | | |
| Agree: | N | Are there more than two functional types? | | | |
| Neutral: | C | I would just have the "increase / decrease their probability to" generate action potentials – is there any synapse which alone can ensure generation of an action potential?? | | | |
| Disagree: |  |  | | | |
|  | | | | | |

| **Statement 11** | | | Agree | Neutral | Disagree |
| --- | --- | --- | --- | --- | --- |
| 1. The chemicals used for communication between neurons at synapses are called transmitters. There are many different types of transmitters, but they usually have only one of two alternative effects on a receiving neuron: excitatory or inhibitory. The duration of the effect can vary from milliseconds to several minutes. | | | 3 | 1 |  |
|  | | | | | |
| Comments from the experts: | | | | | |
| Agree: | N | Use the term “neurotransmitters” | | | |
| Neutral: | C | I would rewrite too "..but the most common effects is excitatory or inhibitory" (why? to not "exclude" more complex modulatory transmitter effects) | | | |
| Disagree: |  |  | | | |
|  | | | | | |

| **Statement 12** | | | Agree | Neutral | Disagree |
| --- | --- | --- | --- | --- | --- |
| 1. A neuron can receive both excitatory and inhibitory signals but can primarily send only one of the types, excitatory or inhibitory, to all its target cells. | | | 3 | 1 |  |
|  | | | | | |
| Comments from the experts: | | | | | |
| Agree: |  |  | | | |
| Neutral: | C | "can usually send.." or something? Aren't there quite some neuron types that create different chemicals for co-release..? Me not sure, but why not state too definite? | | | |
| Disagree: |  |  | | | |
|  | | | | | |

| **Statement 13** | | Agree | Neutral | Disagree |
| --- | --- | --- | --- | --- |
| Neurons are connected in networks, but each neuron makes synapses with specific target cells, not with every cell around them. Thus, a nerve signal from a given neuron will only pass to a selected group of target cells rather than to all its neighboring cells. | | 4 |  |  |
|  | | | | |
| Comments from the experts: | | | | |
| Agree: |  | | | |
| Neutral: |  | | | |
| Disagree: |  | | | |
|  | | | | |

**When nerve signals travel through networks of neurons**

| **Statement 14** | | | Agree | Neutral | Disagree |
| --- | --- | --- | --- | --- | --- |
| The nerve signal carries information about the external environment and the body. It generates our sensations, perceptions, thoughts, feelings and behaviors. | | | 3 |  | 1 |
|  | | | | | |
| Comments from the experts: | | | | | |
| Agree: |  |  | | | |
| Neutral: | C | This is a difficult one. I kind of agree, though I itch a little bit about the impression it gives that one single signal carries the information about a thought. But support to include this. | | | |
| Disagree: | F | There are many other factors than nerve signals involved in *generating* our thoughts | | | |
|  | | | | | |

| **Statement 15** | | | Agree | Neutral | Disagree |
| --- | --- | --- | --- | --- | --- |
| What we sense, perceive, think, feel, and how we behave are mainly determined by two factors:   1. the specific network of neurons through which the nerve signals travel.  - e.g. in sensory parts of the nervous system, this carries information about the *identity* of the stimulus (a particular odor, sound frequency, color, etc.),  1. the time between successive action potentials travelling through the network.  - e.g. in sensory parts of the nervous system, this carries information about the *intensity* of the stimulus. Short time (high frequency) means high intensity and long time (low frequency) means low intensity. | | | 3 |  | 1 |
|  | | | | | |
| Comments from the experts: | | | | | |
| Agree: | N | replace ”factors “with «properties of the nervous system» | | | |
| Neutral: |  |  | | | |
| Disagree: | F | There is a link between neural network and sensation - yes. But there is a huge gap between any neural network and consciousness, perception, and thinking | | | |
|  | | | | | |

| **Statement 16** | | | Agree | Neutral | Disagree |
| --- | --- | --- | --- | --- | --- |
| Information (nerve signals) from different sensory organ, e.g. visual-, auditory-, olfactory-, taste- and touch information, is conveyed to distinct regions of the brain. | | | 3 | 1 |  |
|  | | | | | |
| Comments from the experts: | | | | | |
| Agree: |  |  | | | |
| Neutral: | C | add "distinct for these functions"? ... before they are combined? | | | |
| Disagree: |  |  | | | |
|  | | | | | |

| **Statement 17** | | | Agree | Neutral | Disagree |
| --- | --- | --- | --- | --- | --- |
| The brain has distinct regions for several functions, e.g. the different sensory modalities, language, and different types of behaviors and learning. However, brain regions are interlinked, and a proper function of each requires coordination of nerve signals across many regions. | | | 3 | 1 |  |
|  | | | | | |
| Comments from the experts: | | | | | |
| Agree: | N | Replace “several” with “different” | | | |
| Neutral: | C | and, each function depends on several regions..? But okay. | | | |
| Disagree: |  |  | | | |
|  | | | | | |

| **Statement 18** | | | Agree | Neutral | Disagree |
| --- | --- | --- | --- | --- | --- |
| Nerve signals in the network of neurons responsible for our perceptions, feelings and behaviors interact all the time. This means that our *perceptions* influence how we feel and behave, but also that our *behavior* influences what we perceive and how we feel, and finally that our *feelings* influence what we perceive and how we behave. | | | 4 |  |  |
|  | | | | | |
| Comments from the experts: | | | | | |
| Agree: | C | Nice! | | | |
| Neutral: |  |  | | | |
| Disagree: |  |  | | | |
|  | | | | | |

| **Statement 19** | | | Agree | Neutral | Disagree |
| --- | --- | --- | --- | --- | --- |
| To become *aware of what* we are sensing, the nerve signals in the relevant sensory neurons must be sufficiently strong (sufficient frequency and number of action potentials) to enter specific networks of neurons in the frontal part of the cerebral cortex. | | | 3 | 1 |  |
|  | | | | | |
| Comments from the experts: | | | | | |
| Agree: | N | This depends on certain conditions, right? Maybe you should add that | | | |
| Neutral: | C | This one is complicated. Understand what you mean and it is okay, but there are also all these top-down things, not only the intensity in the sensory neuron. | | | |
| Disagree: |  |  | | | |
|  | | | | | |

| **Statement 20** | | | Agree | Neutral | Disagree |
| --- | --- | --- | --- | --- | --- |
| Not all nerve signals from our senses are sufficiently strong to make us *aware of what* we are sensing. However, the signals can still be strong enough to influence the networks of neurons responsible for our feelings, thoughts and behaviors. Consequently, we cannot always know the reason for what we feel, think and do. | | | 3 | 1 |  |
|  | | | | | |
| Comments from the experts: | | | | | |
| Agree: | N | Replace “know” with “be aware of” | | | |
| Neutral: | C | Same comment as 39. This is complex stuff that we do not understand to the same level as the earlier principles. | | | |
| Disagree: |  |  | | | |
|  | | | | | |

**Plasticity in the nervous system**

| **Statement 21** | | Agree | Neutral | Disagree |
| --- | --- | --- | --- | --- |
| Synapses are formed and changed throughout life, and this process is influenced by individual experiences. | | 4 |  |  |
|  | | | | |
| Comments from the experts: | | | | |
| Agree: |  | | | |
| Neutral: |  | | | |
| Disagree: |  | | | |
|  | | | | |

| **Statement 22** | | Agree | Neutral | Disagree |
| --- | --- | --- | --- | --- |
| A synaptic change typically means that the influence a neuron has on its target neurons becomes stronger or weaker. | | 4 |  |  |
|  | | | | |
| Comments from the experts: | | | | |
| Agree: |  | | | |
| Neutral: |  | | | |
| Disagree: |  | | | |
|  | | | | |

| **Statement 23** | | Agree | Neutral | Disagree |
| --- | --- | --- | --- | --- |
| When you learn, memories are stored as a change in specific synapses in the network of neurons involved in the learning activity. Some synapses may be strengthened, others weakened. | | 4 |  |  |
|  | | | | |
| Comments from the experts: | | | | |
| Agree: |  | | | |
| Neutral: |  | | | |
| Disagree: |  | | | |
|  | | | | |

| **Statement 24** | | | Agree | Neutral | Disagree |
| --- | --- | --- | --- | --- | --- |
| Regularly engaging in learning activities enhances brain function and defers age-related decline in brain function. | | | 3 | 1 |  |
|  | | | | | |
| Comments from the experts: | | | | | |
| Agree: |  |  | | | |
| Neutral: | C | Not wrong, and I like that the point is included. Just worried if it can be understood very literally or misunderstood. For example, you can't "train yourself out of degeneration", even if it is one factor. And too much (stress) is not good either. I think it is good to convey that it is healthy to use your brain in healthy ways, but its not the case that it is your "fault" if you still have an agerelated decline in brain function. How to convey this correctly to school kids? But probably you have thought it well through! (And I like this one better than some previous version which I think I criticised!) | | | |
| Disagree: |  |  | | | |
|  | | | | | |

| **Statement 25** | | | Agree | Neutral | Disagree |
| --- | --- | --- | --- | --- | --- |
| The brains’ ability to change through experience varies during lifetime and differs between brain regions. In early childhood, sensory regions are particularly susceptible, and the changes may be large and irreversible. Whereas some changes are crucial for normal development, others are detrimental, depending on the experience. | | | 3 | 1 |  |
|  | | | | | |
| Comments from the experts: | | | | | |
| Agree: |  |  | | | |
| Neutral: | C | Similar to above (49) - I just wonder if there is any way that this one can be taken very literally or to the extreme, like thinking "if only I didn't experience (blah) then my brain would not have been irreversibly damaged" :P I like the point, I would just be very cautious for possible misunderstandings. | | | |
| Disagree: |  |  | | | |
|  | | | | | |

Round 4

**Gross anatomy and function**

| **Statement 1** | | Agree | Neutral | Disagree |
| --- | --- | --- | --- | --- |
| The vertebrate nervous system comprises the brain, the spinal cord, ganglia and nerves. The brain and the spinal cord make up the central nervous system (CNS), whereas ganglia and nerves make up the peripheral nervous system (PNS). The PNS connects the CNS with the rest of the body | | 11 |  |  |
|  | | | | |
| Comments from the experts: | | | | |
| Agree: |  | | | |
| Neutral: |  | | | |
| Disagree: |  | | | |
|  | | | | |

| **Statement 2** | | Agree | Neutral | Disagree |
| --- | --- | --- | --- | --- |
| The PNS consists of a sensory division which carries information about the external environment and the body into the CNS, and a motor division which carries information from the CNS to muscles and glands. Through this architecture, the nervous system influences and is influenced by all other organ systems (e.g., cardiovascular-, gastrointestinal-, muscular-, immune-, exocrine- and endocrine systems). | | 11 |  |  |
|  | | | | |
| Comments from the experts: | | | | |
| Agree: |  | | | |
| Neutral: |  | | | |
| Disagree: |  | | | |
|  | | | | |

| **Statement 3** | | Agree | Neutral | Disagree |
| --- | --- | --- | --- | --- |
| 1. The motor division is commonly divided into a voluntary system (the somatic system) controlling our skeletal muscles, and an involuntary system (the autonomous system) controlling visceral organs. However, the two systems interact to support compatible physiological, emotional and behavioral responses | | 11 |  |  |
|  | | | | |
| Comments from the experts: | | | | |
| Agree: |  | | | |
| Neutral: |  | | | |
| Disagree: |  | | | |
|  | | | | |

| **Statement 4** | | Agree | Neutral | Disagree |
| --- | --- | --- | --- | --- |
| 1. The brain has a continuous self-sustaining generation of nerve signals. These nerve signals are not dependent on sensory input but can be modified by it. | | 11 |  |  |
|  | | | | |
| Comments from the experts: | | | | |
| Agree: |  | | | |
| Neutral: |  | | | |
| Disagree: |  | | | |
|  | | | | |

**Cell types and functional units**

| **Statement 5** | | Agree | Neutral | Disagree |
| --- | --- | --- | --- | --- |
| The nervous system consists of two major classes of cells:  a) Neurons, which receive, process and send nerve signals,  b) Glial cells, which protect and support the function of neurons | | 11 |  |  |
|  | | | | |
| Comments from the experts: | | | | |
| Agree: |  | | | |
| Neutral: |  | | | |
| Disagree: |  | | | |
|  | | | | |

| **Statement 6** | | | Agree | Neutral | Disagree |
| --- | --- | --- | --- | --- | --- |
| A neuron usually consists of three main structures:  a) a cell body  b) dendrites which receive nerve signals from several other cells.  c) a single branching axon with many terminals, which sends nerve signals to several other cells | | | 11 |  |  |
|  | | | | | |
| Comments from the experts: | | | | | |
| Agree: | O | Does the axon terminal deserve its own structure? | | | |
| Neutral: |  |  | | | |
| Disagree: |  |  | | | |
|  | | | | | |

| **Statement 7** | | | Agree | Neutral | Disagree |
| --- | --- | --- | --- | --- | --- |
| 1. There are many types of neurons and they are commonly classified in two ways:   a) by where they receive and send signals:   1. Sensory neurons, which receive signals (light, sound, etc.) from outside the nervous system, transform them into nerve signals and send them to neurons in the central nervous system. 2. Motor neurons, which receive nerve signals from neurons in the central nervous system and send them to muscles and glands. 3. Local interneurons, which receive nerve signals from neurons in a region of the nervous system and send them to neurons located within the same region. 4. Projection interneurons, which receive nerve signals from neurons in one region of the nervous system and send them to neurons in another region.   b) by what effect they have on their target cells:   - - 1. excitatory neurons, which have a stimulating effect on nerve signal generation     2. inhibitory neurons, which have an inhibitory effect on nerve signal generation | | | 11 |  |  |
|  | | | | | |
| Comments from the experts: | | | | | |
| Agree: | C | Projection interneuron sounds strange, but ok | | | |
|  | C | Excitation and inhibition is mentioned several places and may therefore appear confusing. | | | |
| Neutral: |  |  | | | |
| Disagree: |  |  | | | |
|  | | | | | |

**The nerve signal**

| **Statement 8** | | | Agree | Neutral | Disagree |
| --- | --- | --- | --- | --- | --- |
| The typical nerve signal is generated by neurons and consists of three types of pulses:  a) Graded electrical pulses (receptor potentials and synaptic potentials) which primarily flow from the dendrites to the beginning of the axon.  b) Ungraded electrical pulses (action potentials) which primarily flow from the beginning of the axon to the axon terminals.  c) Graded chemical pulses which primarily flow from the axon terminals, over an extracellular gap, to the dendrites of a receiving neuron. The chemical pulses propagate much slower than the electrical pulses. | | | 11 |  |  |
|  | | | | | |
| Comments from the experts: | | | | | |
| Agree: | B | Consider having the word “synapse” in principle 8C. It would have been orienting if it was specifically stated that you mean a synapse?  But only if it’s true— if you mean extrasynaptic chemical transmission too, then please ignore my comment | | | |
| Neutral: |  |  | | | |
| Disagree: |  |  | | | |
|  | | | | | |

| **Statement 9** | | Agree | Neutral | Disagree |
| --- | --- | --- | --- | --- |
| 1. When a neuron receives graded pulses of chemicals, it generates graded electrical pulses. If the graded electrical pulses are larger than a certain voltage value (threshold), the neuron generates action potentials successively until the voltage value goes below threshold. When the action potentials reach the axon terminals, the neuron releases graded pulses of chemicals | | 11 |  |  |
|  | | | | |
| Comments from the experts: | | | | |
| Agree: |  | | | |
| Neutral: |  | | | |
| Disagree: |  | | | |
|  | | | | |

**Connections between neurons**

| **Statement 10** | | Agree | Neutral | Disagree |
| --- | --- | --- | --- | --- |
| Nerve signals pass from one neuron to the next at locations called synapses which are specialized zones for communication between neurons. | | 11 |  |  |
|  | | | | |
| Comments from the experts: | | | | |
| Agree: |  | | | |
| Neutral: |  | | | |
| Disagree: |  | | | |
|  | | | | |

| **Statement 11** | | Agree | Neutral | Disagree |
| --- | --- | --- | --- | --- |
| There are two main functional types of synapses:  a) Excitatory synapses which make the receiving neurons generate action potentials or increase their probability to do so. b) Inhibitory synapses which prevent the receiving neurons from generating action potentials or decrease their probability to do so | | 11 |  |  |
|  | | | | |
| Comments from the experts: | | | | |
| Agree: |  | | | |
| Neutral: |  | | | |
| Disagree: |  | | | |
|  | | | | |

| **Statement 12** | | Agree | Neutral | Disagree |
| --- | --- | --- | --- | --- |
| 1. The chemicals used for communication between neurons at synapses are called neurotransmitters. There are many different types of neurotransmitters, but the most common effect on a receiving neuron is excitatory or inhibitory. The duration of the effect can vary from milliseconds to several minutes. | | 11 |  |  |
|  | | | | |
| Comments from the experts: | | | | |
| Agree: |  | | | |
| Neutral: |  | | | |
| Disagree: |  | | | |
|  | | | | |

| **Statement 13** | | Agree | Neutral | Disagree |
| --- | --- | --- | --- | --- |
| A neuron can receive both excitatory and inhibitory signals but can usually send only one of the types, excitatory or inhibitory, to all its target cells. | | 11 |  |  |
|  | | | | |
| Comments from the experts: | | | | |
| Agree: |  | | | |
| Neutral: |  | | | |
| Disagree: |  | | | |
|  | | | | |

| **Statement 14** | | Agree | Neutral | Disagree |
| --- | --- | --- | --- | --- |
| Neurons are connected in networks, but each neuron makes synapses with specific target cells, not with every cell around them. Thus, a nerve signal from a given neuron will only pass to a selected group of target cells rather than to all its neighboring cells. | | 11 |  |  |
|  | | | | |
| Comments from the experts: | | | | |
| Agree: |  | | | |
| Neutral: |  | | | |
| Disagree: |  | | | |
|  | | | | |

**When nerve signals travel through networks of neurons**

| **Statement 15** | | | Agree | Neutral | Disagree |
| --- | --- | --- | --- | --- | --- |
| The nerve signals carry information about the external environment and the body. This information can generate our sensations, perceptions, thoughts, feelings and behaviors | | | 10 |  | 1 |
|  | | | | | |
| Comments from the experts: | | | | | |
| Agree: | C | Is it possible to improve the formulation «this info can generate...»? | | | |
| Neutral: |  |  | | | |
| Disagree: | F | There are elements we still do not know | | | |
|  | | | | | |

| **Statement 16** | | | Agree | Neutral | Disagree |
| --- | --- | --- | --- | --- | --- |
| What we sense, perceive, think, feel, and how we behave are mainly determined by two properties of the nervous system:  a) the specific network of neurons through which the nerve signals travel.  - e.g. in sensory parts of the nervous system, this carries information about the *identity* of the stimulus (a particular odor, sound frequency, color, etc.),  b) the time between successive action potentials travelling through the network.  - e.g. in sensory parts of the nervous system, this carries information about the *intensity* of the stimulus. Short time (high frequency) means high intensity and long time (low frequency) means low intensity. | | | 9 |  | 2 |
|  | | | | | |
| Comments from the experts: | | | | | |
| Agree: |  |  | | | |
| Neutral: |  |  | | | |
| Disagree: | E | It is weakly formulated and probably doesn’t deserve a place on the list | | | |
|  | | | | | |

| **Statement 17** | | | Agree | Neutral | Disagree |
| --- | --- | --- | --- | --- | --- |
| Information from different sensory organs, like visual-, auditory-, olfactory-, taste- and tactile information, is carried by nerve signals travelling along neurons to distinct regions of the brain. | | | 11 |  |  |
|  | | | | | |
| Comments from the experts: | | | | | |
| Agree: | N | Replace “several” with “different” | | | |
| Neutral: | C | and, each function depends on several regions..? But okay. | | | |
| Disagree: |  |  | | | |
|  | | | | | |

| **Statement 18** | | Agree | Neutral | Disagree |
| --- | --- | --- | --- | --- |
| The brain has distinct regions for different functions including a variety of sensations, behaviors, language, etc. However, brain regions are interlinked by neurons, and each function depends on several regions. | | 11 |  |  |
|  | | | | |
| Comments from the experts: | | | | |
| Agree: |  | | | |
| Neutral: |  | | | |
| Disagree: |  | | | |
|  | | | | |

| **Statement 19** | | | Agree | Neutral | Disagree |
| --- | --- | --- | --- | --- | --- |
| The network of neurons responsible for our perceptions, emotions and behaviors interact all the time. This means that our *perceptions* influence how we feel and behave, but also that our *behavior* influences what we perceive and how we feel, and finally that our *feelings* influence what we perceive and how we behave. | | | 10 |  | 1 |
|  | | | | | |
| Comments from the experts: | | | | | |
| Agree: |  |  | | | |
| Neutral: |  |  | | | |
| Disagree: | E | Principle 19 would work perfectly well without referring to feelings, which make it all sound a bit pseudoscientific | | | |
|  | | | | | |

| **Statement 20** | | Agree | Neutral | Disagree |
| --- | --- | --- | --- | --- |
| To become *conscious of what* we are sensing, the nerve signals in the sensory neurons must be sufficiently strong (sufficient number and frequency of action potentials) to enter specific networks of neurons in the frontal part of the cerebral cortex. The required strength varies depending on how we feel, what we do and what the stimulus is. | | 11 |  |  |
|  | | | | |
| Comments from the experts: | | | | |
| Agree: |  | | | |
| Neutral: |  | | | |
| Disagree: |  | | | |
|  | | | | |

| **Statement 21** | | | Agree | Neutral | Disagree |
| --- | --- | --- | --- | --- | --- |
| Not all nerve signals from our senses are sufficiently strong to make us *conscious of what* we are sensing. However, the signals can still be strong enough to influence the networks of neurons responsible for our feelings, thoughts and behaviors. Consequently, we cannot always be aware of the reason for what we feel, think and do. | | | 11 |  |  |
|  | | | | | |
| Comments from the experts: | | | | | |
| Agree: | C | Sounds a bit strange, but ok | | | |
| Neutral: |  |  | | | |
| Disagree: |  |  | | | |
|  | | | | | |

**Plasticity in the nervous system**

| **Statement 22** | | Agree | Neutral | Disagree |
| --- | --- | --- | --- | --- |
| Synapses are formed and changed throughout life, and this process is influenced by individual experiences. | | 11 |  |  |
|  | | | | |
| Comments from the experts: | | | | |
| Agree: |  | | | |
| Neutral: |  | | | |
| Disagree: |  | | | |
|  | | | | |

| **Statement 23** | | Agree | Neutral | Disagree |
| --- | --- | --- | --- | --- |
| A synaptic change typically means that the influence a neuron has on its target neurons becomes stronger or weaker. | | 11 |  |  |
|  | | | | |
| Comments from the experts: | | | | |
| Agree: |  | | | |
| Neutral: |  | | | |
| Disagree: |  | | | |
|  | | | | |

| **Statement 24** | | Agree | Neutral | Disagree |
| --- | --- | --- | --- | --- |
| When you learn, each memory is stored as a change in specific synapses in the network of neurons involved in the learning activity. Some synapses may be strengthened, others weakened. | | 11 |  |  |
|  | | | | |
| Comments from the experts: | | | | |
| Agree: |  | | | |
| Neutral: |  | | | |
| Disagree: |  | | | |
|  | | | | |

| **Statement 25** | | Agree | Neutral | Disagree |
| --- | --- | --- | --- | --- |
| Engaging in learning activities enhances brain function and defers age-related decline in brain function. | | 11 |  |  |
|  | | | | |
| Comments from the experts: | | | | |
| Agree: |  | | | |
| Neutral: |  | | | |
| Disagree: |  | | | |
|  | | | | |

| **Statement 26** | | Agree | Neutral | Disagree |
| --- | --- | --- | --- | --- |
| The brain’s ability to change through experience varies over the lifetime and differs between brain regions. Sensory regions are particularly sensitive during early childhood, whereas frontal regions involved in cognitive functions are more sensitive later and the sensitivity lasts longer. Some changes are crucial for normal development, whereas others are detrimental, depending on the experience. | | 11 |  |  |
|  | | | | |
| Comments from the experts: | | | | |
| Agree: |  | | | |
| Neutral: |  | | | |
| Disagree: |  | | | |
|  | | | | |
